# Supplementary material for: Assessing the ecological risk of heavy metal sediment contamination from Port Everglades Florida USA
Source: PeerJ. 2023 Nov 14;11:e16152. doi: 10.7717/peerj.16152 (PMC10655720; doi:10.7717/peerj.16152)
Supplement: Supplemental Information 1 — The left side of the core at 0 cm is the top (surface). Munsell sediment color chart is also shown. [file peerj-11-16152-s001.pdf]

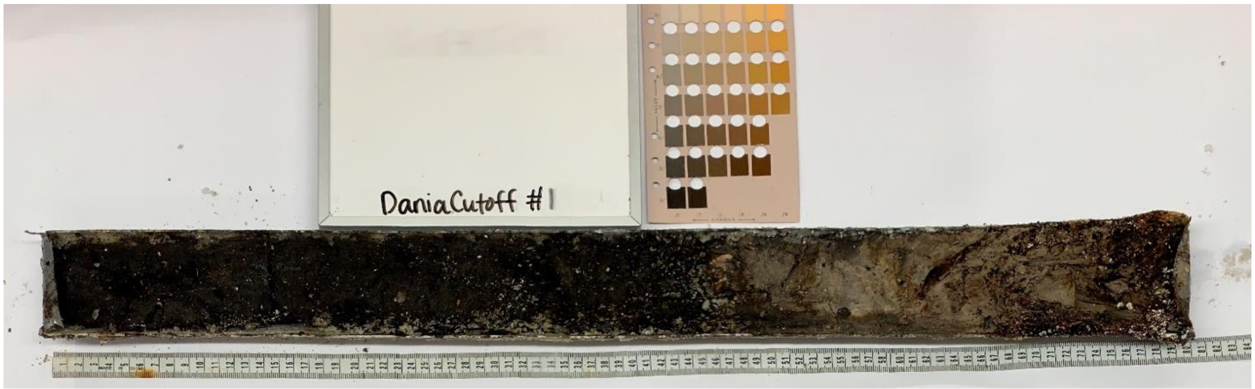
Dania Cutoff #1

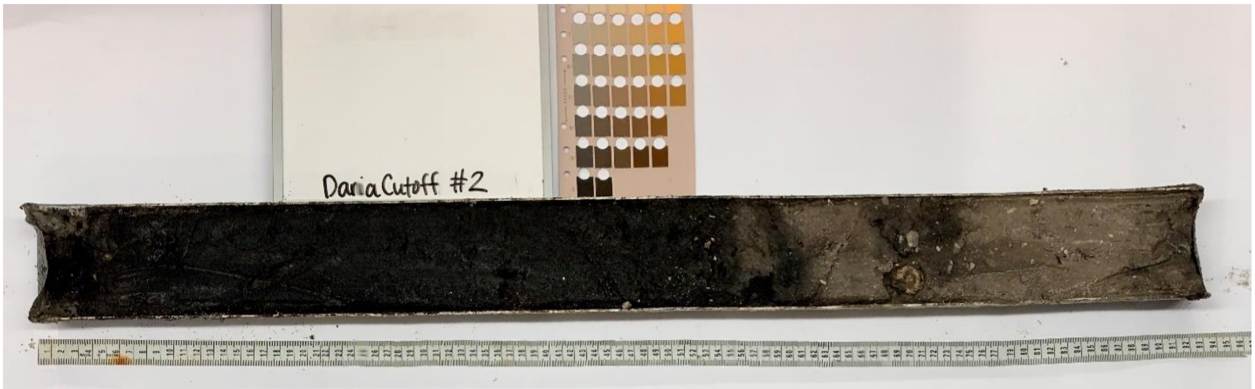
Dania Cutoff #2

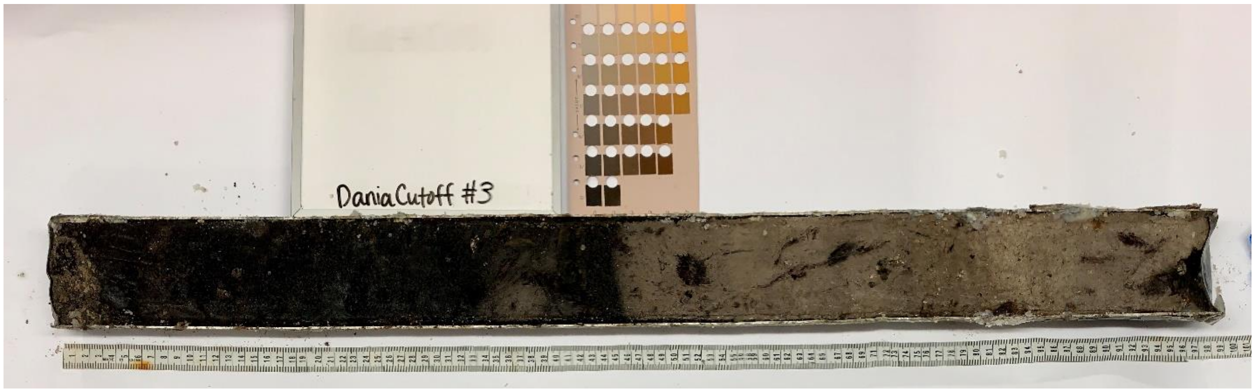
Dania Cutoff #3

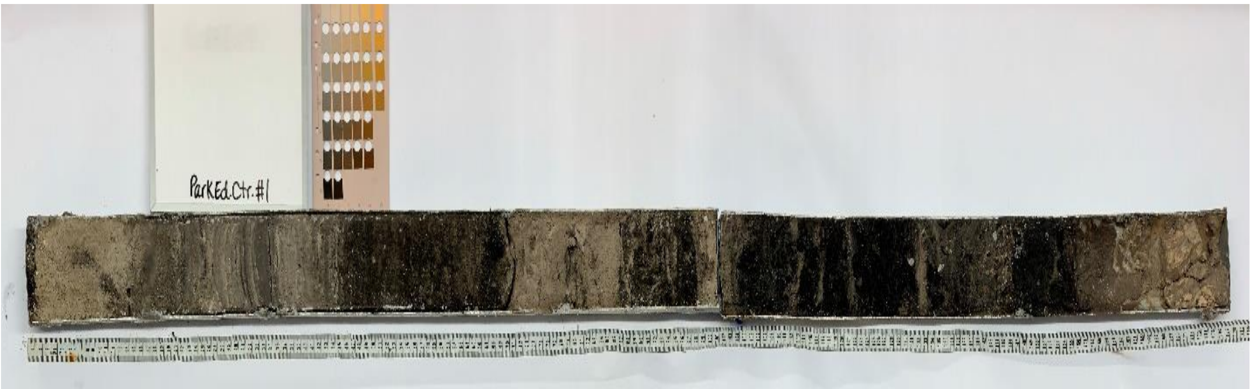
Park Ed. Ctr. #1

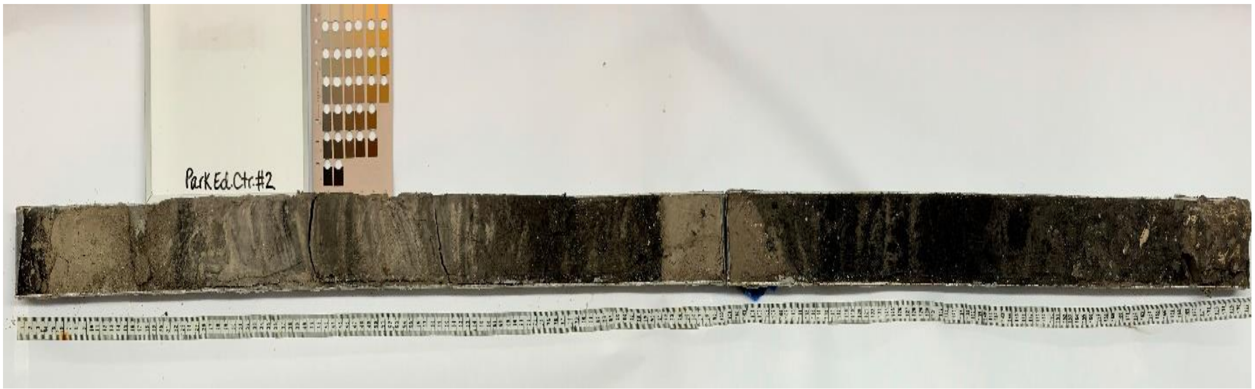
Park Ed. Ctr. #2

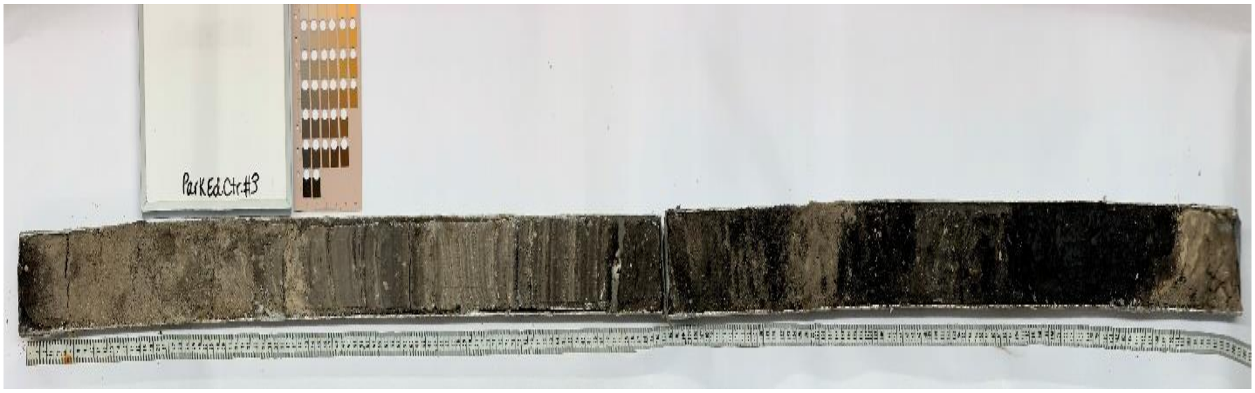
Park Ed. Ctr. #3

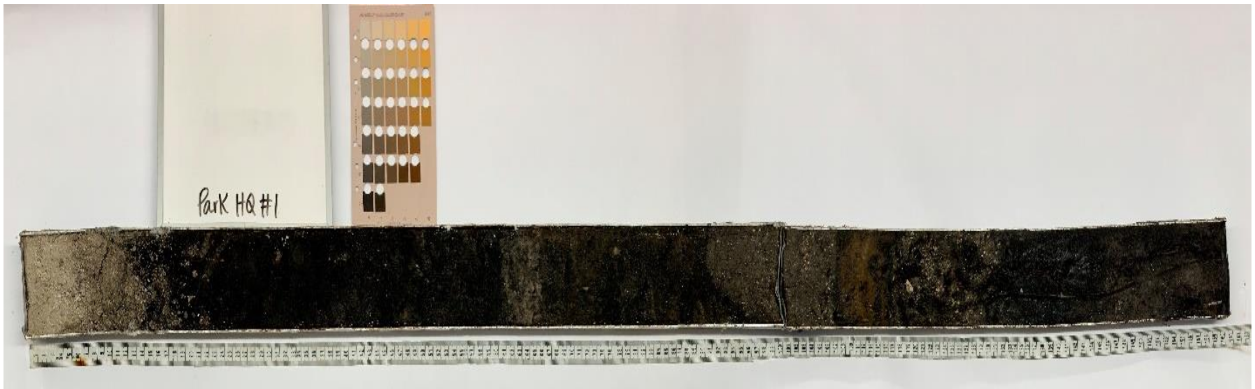
Park HQ #1

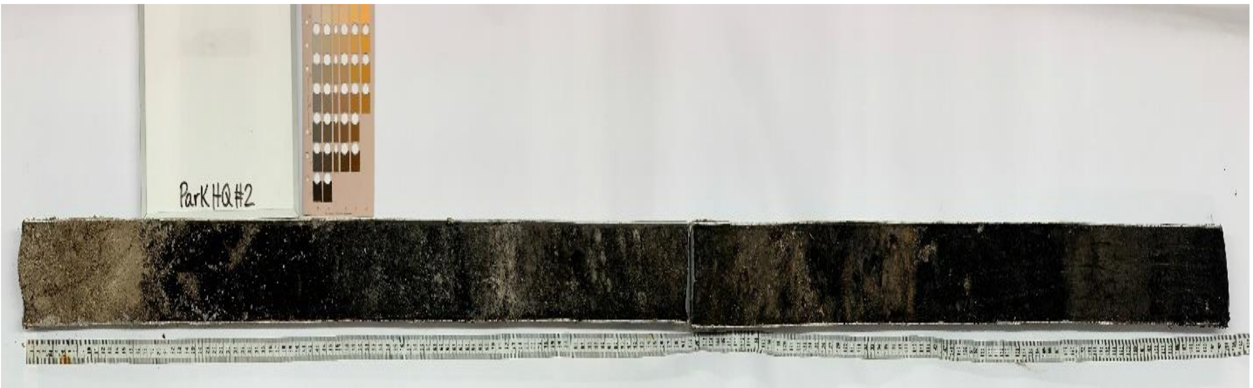
Park HQ #2

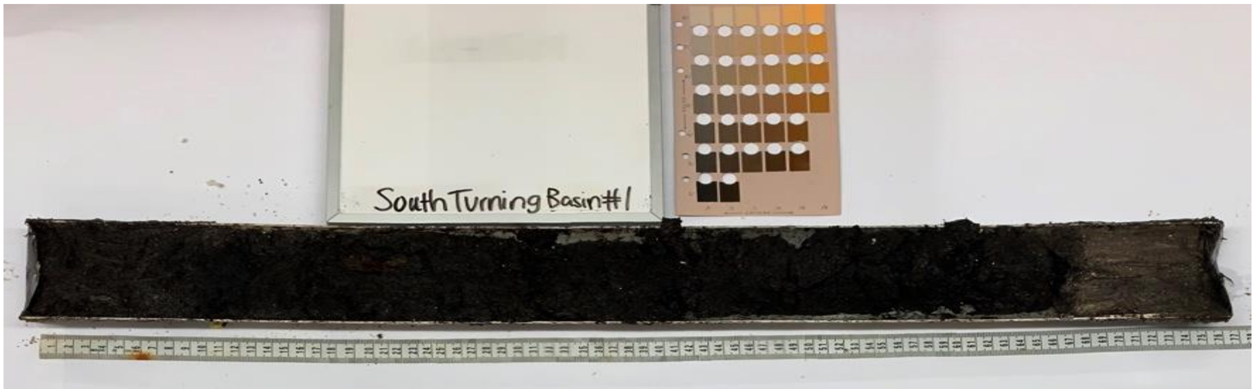
South Turning Basin #1

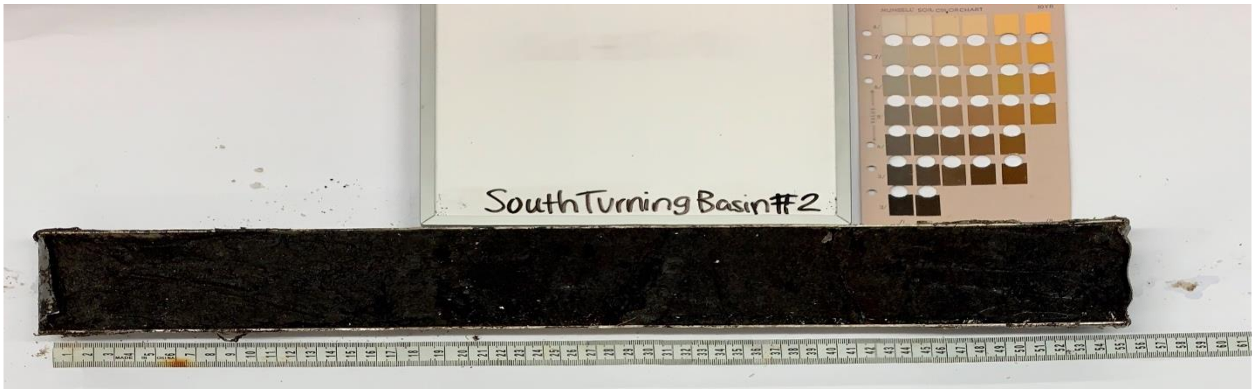
South Turning Basin #2

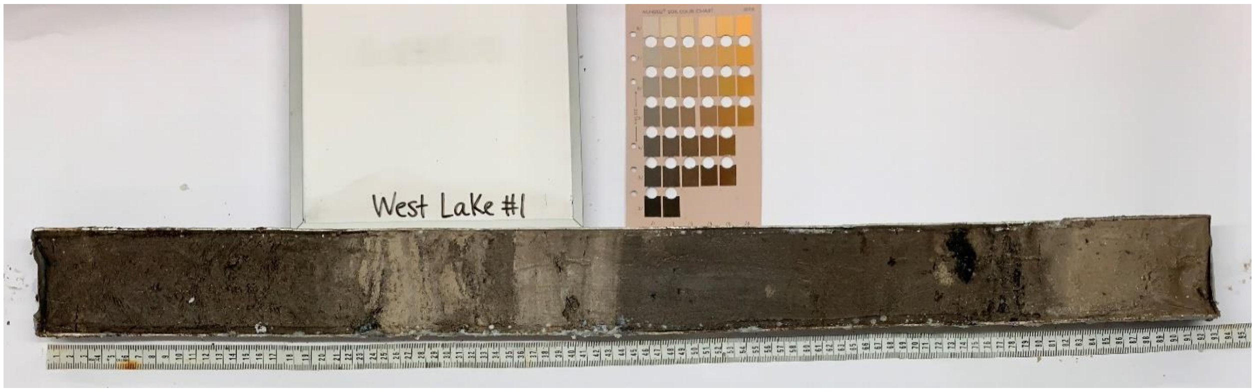
West Lake #1

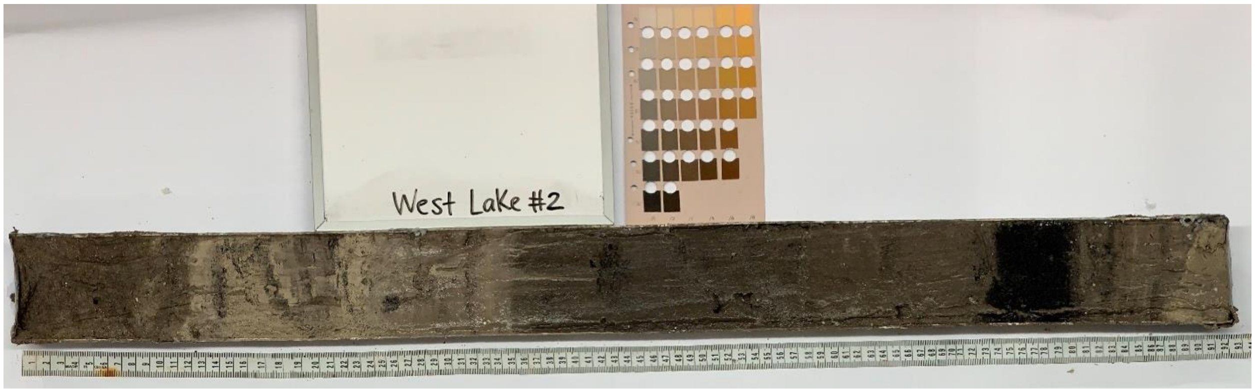

Figure S1. Split sediment cores for Dania Cutoff Canal (DCC), Park Education Center (PEC), Park Headquarters (PHQ), South Turning Basin (STB), and West Lake (WL). The left side of the core at 0 cm is the top (surface). Munsell sediment color chart is also shown.
