# Supplementary material for: Assessing the ecological risk of heavy metal sediment contamination from Port Everglades Florida USA
Source: PeerJ. 2023 Nov 14;11:e16152. doi: 10.7717/peerj.16152 (PMC10655720; doi:10.7717/peerj.16152)
Supplement: Supplemental Information 2 [file peerj-11-16152-s002.docx]

**Table S1**. Inductively coupled plasma mass spectrometer (ICPMS) detection limits (µg/g) for the 14 heavy metals tested.

| Heavy Metals | Detection Limit |
| --- | --- |
| As | 0.00003 |
| Cd | 0.00001 |
| Cr | 0.0001 |
| Co | 0.00002 |
| Cu | 0.005 |
| Pb | 0.0004 |
| Mn | 0.00008 |
| Mo | 0.0001 |
| Hg | <0.00001 |
| Ni | 0.0005 |
| Se | 0.00003 |
| Sn | 0.001 |
| V | 0.00004 |
| Zn | 0.02 |
