# Supplementary material for: Assessing the ecological risk of heavy metal sediment contamination from Port Everglades Florida USA
Source: PeerJ. 2023 Nov 14;11:e16152. doi: 10.7717/peerj.16152 (PMC10655720; doi:10.7717/peerj.16152)
Supplement: Supplemental Information 3 [file peerj-11-16152-s003.docx]

**Table S2**. Recovery % for standard reference material, SRM 2702-inorganics in marine sediment to evaluate reliability of analytical method.

| **Heavy Metal** | **%** |
| --- | --- |
| As | 73 |
| Cd | 89 |
| Cr | 62 |
| Co | 71 |
| Cu | 76 |
| Pb | 79 |
| Mn | 82 |
| Hg | 66 |
| Mo | 48 |
| Ni | 57 |
| Se | 76 |
| Sn | 31 |
| V | 66 |
| Zn | 77 |
