# Supplementary material for: Assessing the ecological risk of heavy metal sediment contamination from Port Everglades Florida USA
Source: PeerJ. 2023 Nov 14;11:e16152. doi: 10.7717/peerj.16152 (PMC10655720; doi:10.7717/peerj.16152)
Supplement: Supplemental Information 4 — NA is not available. [file peerj-11-16152-s004.docx]

**Table S3**. Threshold Effect Level (TEL), Probable Effect Level (PEL), and continental crust values. NA is not available.

| **Heavy metals** | **Sediment Quality Assessment Guidelines**  **(µg/g)** | | **Continental Crust**  **(µg/g)** |
| --- | --- | --- | --- |
|  | **TEL** | **PEL** |  |
| As | 7.24 | 41.6 | 1.5 |
| Cd | 0.676 | 4.21 | 0.098 |
| Cr | 52.3 | 160 | 35 |
| Co | NA | NA | 10 |
| Cu | 18.7 | 108 | 25 |
| Pb | 30.2 | 112 | 20 |
| Mn | NA | NA | 600 |
| Hg | 0.13 | 0.626 | 0.098 |
| Mo | NA | NA | 1.5 |
| Ni | 15.9 | 42.8 | 20 |
| Se | NA | NA | 50 |
| Sn | NA | NA | 5.5 |
| V | NA | NA | 60 |
| Zn | 124 | 271 | 71 |
