# Supplementary material for: Assessing the ecological risk of heavy metal sediment contamination from Port Everglades Florida USA
Source: PeerJ. 2023 Nov 14;11:e16152. doi: 10.7717/peerj.16152 (PMC10655720; doi:10.7717/peerj.16152)
Supplement: Supplemental Information 5 — N/a = end of sediment core. N/d = Not detected. For statistical purposes half of the limit of detection was used for n/d samples. Bold indicate maximum concentration values. [file peerj-11-16152-s005.docx]

| Table S4. Dania Cut-off Canal 1 heavy metal concentrations (µg/g) by sediment core depth (cm), minimum (min), maximum (max), median, arithmetic mean (mean), and geometric mean (geomean). | | | | | | | | | | | | | | |
| --- | --- | --- | --- | --- | --- | --- | --- | --- | --- | --- | --- | --- | --- | --- |
| cm | Mo | Cd | Hg | Pb | V | Cr | Mn | Co | Ni | Zn | Cu | Sn | As | Se |
| 5 | 16.1 | 0.254 | n/d | 6.35 | 32.4 | **34.9** | 76.2 | 0.620 | **11.3** | **102** | **175** | 52.2 | 36.4 | **2.96** |
| 10 | 2.97 | 0.051 | n/d | 3.01 | 7.72 | 7.41 | 18.4 | 0.160 | 2.26 | 73.9 | 151 | 14.8 | 11.4 | 0.659 |
| 15 | 20.1 | 0.171 | n/d | 2.11 | 24.8 | 25.7 | 48.2 | 0.470 | 8.72 | 9.30 | 13.4 | 45.2 | 42.9 | 2.30 |
| 20 | 36.3 | 0.260 | n/d | 2.00 | 31.3 | 22.8 | 52.4 | 0.560 | 8.99 | 13.1 | 4.36 | 54.3 | 66.2 | 2.60 |
| 25 | 36.2 | **0.303** | n/d | 2.36 | 37.4 | 27.8 | 62.2 | 0.620 | 10.1 | 17.3 | 20.3 | **54.5** | **73.2** | 2.59 |
| 30 | 24.9 | 0.192 | n/d | 1.52 | 23.3 | 15.3 | 43.4 | 0.470 | 5.98 | 4.07 | 3.51 | 32.7 | 53.2 | 1.70 |
| 35 | 18.7 | 0.149 | n/d | 2.72 | 24.5 | 14.3 | 42.3 | 0.490 | 5.14 | 12.5 | 9.42 | 24.4 | 36.3 | 1.39 |
| 40 | **45.9** | 0.230 | n/d | 1.71 | **38.9** | 15.4 | 70.5 | 0.760 | 7.29 | 10.0 | 23.4 | 1.03 | 61.2 | 2.01 |
| 45 | 17.9 | 0.080 | n/d | 1.07 | 15.0 | 6.25 | 63.7 | 0.510 | 3.11 | 5.37 | 3.70 | 32.1 | 26.7 | 1.14 |
| 50 | 7.06 | 0.03 | n/d | 0.230 | 5.10 | 1.96 | 130 | 0.340 | 1.23 | 1.48 | 0.532 | 25.3 | 12.6 | 0.494 |
| 55 | 3.72 | 0.011 | n/d | 0.060 | 1.25 | 0.824 | 119 | 0.330 | 0.760 | 1.01 | 0.215 | 16.1 | 13.5 | 0.434 |
| 60 | 4.62 | 0.012 | n/d | 0.089 | 2.31 | 0.788 | 147 | 0.290 | 0.830 | 1.01 | 0.381 | 16.2 | 14.0 | 0.458 |
| 65 | 1.91 | 0.010 | n/d | 0.150 | 0.950 | 0.701 | 144 | 0.260 | 0.523 | 0.807 | 0.288 | 6.13 | 12.3 | 0.319 |
| 70 | 0.878 | 0.011 | n/d | 0.350 | 1.13 | 0.847 | **163** | 0.350 | 0.890 | 75.4 | 55.4 | 1.28 | 17.9 | 0.259 |
| 75 | 2.93 | 0.004 | n/d | **8.21** | 5.92 | 13.6 | 79.7 | **1.81** | 3.57 | 14.6 | 10.3 | 0.054 | 15.0 | 0.681 |
| 80 | n/a | n/a | n/a | n/a | n/a | n/a | n/a | n/a | n/a | n/a | n/a | n/a | n/a | n/a |
| 85 | n/a | n/a | n/a | n/a | n/a | n/a | n/a | n/a | n/a | n/a | n/a | n/a | n/a | n/a |
| 90 | n/a | n/a | n/a | n/a | n/a | n/a | n/a | n/a | n/a | n/a | n/a | n/a | n/a | n/a |
| 95 | n/a | n/a | n/a | n/a | n/a | n/a | n/a | n/a | n/a | n/a | n/a | n/a | n/a | n/a |
| 100 | n/a | n/a | n/a | n/a | n/a | n/a | n/a | n/a | n/a | n/a | n/a | n/a | n/a | n/a |
| 105 | n/a | n/a | n/a | n/a | n/a | n/a | n/a | n/a | n/a | n/a | n/a | n/a | n/a | n/a |
| 110 | n/a | n/a | n/a | n/a | n/a | n/a | n/a | n/a | n/a | n/a | n/a | n/a | n/a | n/a |
| 115 | n/a | n/a | n/a | n/a | n/a | n/a | n/a | n/a | n/a | n/a | n/a | n/a | n/a | n/a |
| 120 | n/a | n/a | n/a | n/a | n/a | n/a | n/a | n/a | n/a | n/a | n/a | n/a | n/a | n/a |
| 125 | n/a | n/a | n/a | n/a | n/a | n/a | n/a | n/a | n/a | n/a | n/a | n/a | n/a | n/a |
| 130 | n/a | n/a | n/a | n/a | n/a | n/a | n/a | n/a | n/a | n/a | n/a | n/a | n/a | n/a |
| 135 | n/a | n/a | n/a | n/a | n/a | n/a | n/a | n/a | n/a | n/a | n/a | n/a | n/a | n/a |
| 140 | n/a | n/a | n/a | n/a | n/a | n/a | n/a | n/a | n/a | n/a | n/a | n/a | n/a | n/a |
| 145 | n/a | n/a | n/a | n/a | n/a | n/a | n/a | n/a | n/a | n/a | n/a | n/a | n/a | n/a |
| 150 | n/a | n/a | n/a | n/a | n/a | n/a | n/a | n/a | n/a | n/a | n/a | n/a | n/a | n/a |
| 155 | n/a | n/a | n/a | n/a | n/a | n/a | n/a | n/a | n/a | n/a | n/a | n/a | n/a | n/a |
| 160 | n/a | n/a | n/a | n/a | n/a | n/a | n/a | n/a | n/a | n/a | n/a | n/a | n/a | n/a |
| 165 | n/a | n/a | n/a | n/a | n/a | n/a | n/a | n/a | n/a | n/a | n/a | n/a | n/a | n/a |
| 170 | n/a | n/a | n/a | n/a | n/a | n/a | n/a | n/a | n/a | n/a | n/a | n/a | n/a | n/a |
| 175 | n/a | n/a | n/a | n/a | n/a | n/a | n/a | n/a | n/a | n/a | n/a | n/a | n/a | n/a |
| 180 | n/a | n/a | n/a | n/a | n/a | n/a | n/a | n/a | n/a | n/a | n/a | n/a | n/a | n/a |
| 185 | n/a | n/a | n/a | n/a | n/a | n/a | n/a | n/a | n/a | n/a | n/a | n/a | n/a | n/a |
| 190 | n/a | n/a | n/a | n/a | n/a | n/a | n/a | n/a | n/a | n/a | n/a | n/a | n/a | n/a |
| 195 | n/a | n/a | n/a | n/a | n/a | n/a | n/a | n/a | n/a | n/a | n/a | n/a | n/a | n/a |
| 200 | n/a | n/a | n/a | n/a | n/a | n/a | n/a | n/a | n/a | n/a | n/a | n/a | n/a | n/a |
| min | 0.878 | 0.004 | n/d | 0.060 | 0.950 | 0.701 | 18.4 | 0.160 | 0.523 | 0.807 | 0.215 | 0.054 | 11.4 | 0.259 |
| max | 45.9 | 0.303 | n/d | 8.21 | 38.9 | 34.9 | 163 | 1.81 | 11.3 | 102 | 175 | 54.5 | 73.2 | 2.96 |
| median | 16.1 | 0.080 | n/d | 1.71 | 15.0 | 13.6 | 70.5 | 0.470 | 3.57 | 10.0 | 9.42 | 24.4 | 26.7 | 1.14 |
| mean | 16.0 | 0.118 | n/d | 2.13 | 16.8 | 12.6 | 84.0 | 0.536 | 4.71 | 22.8 | 31.4 | 25.1 | 32.9 | 1.33 |
| geomean | 9.38 | 0.06 | nd | 1.02 | 9.21 | 6.61 | 70.69 | 0.47 | 3.07 | 8.90 | 6.83 | 10.17 | 27.00 | 1.00 |

N/a = end of sediment core. N/d = Not detected. For statistical purposes half of the limit of detection was used for n/d samples. Bold indicate maximum concentration values.
