# Supplementary material for: Assessing the ecological risk of heavy metal sediment contamination from Port Everglades Florida USA
Source: PeerJ. 2023 Nov 14;11:e16152. doi: 10.7717/peerj.16152 (PMC10655720; doi:10.7717/peerj.16152)
Supplement: Supplemental Information 6 — N/a = end of sediment core. N/d = Not detected. For statistical purposes half of the limit of detection was used for n/d samples. Bold indicate maximum concentration values. [file peerj-11-16152-s006.docx]

| **Table S5**. Dania Cut-off Canal 2 heavy metal concentrations (µg/g) by sediment core depth (cm), minimum (min), maximum (max), median, arithmetic mean (mean), and geometric mean (geomean). | | | | | | | | | | | | | | |
| --- | --- | --- | --- | --- | --- | --- | --- | --- | --- | --- | --- | --- | --- | --- |
| cm | Mo | Cd | Hg | Pb | V | Cr | Mn | Co | Ni | Zn | Cu | Sn | As | Se |
| 5 | 0.476 | 0.010 | n/d | 1.43 | 2.00 | 2.01 | 5.14 | 0.037 | 0.47 | 5.17 | 3.90 | 0.730 | 0.803 | 0.048 |
| 10 | 45.9 | 0.460 | n/d | 4.00 | 41.5 | 56.8 | 47.0 | 0.921 | 18.6 | 10.3 | 11.9 | 90.2 | 44.3 | 4.55 |
| 15 | 44.1 | 0.390 | n/d | 3.62 | 47.2 | **51.8** | 45.2 | 0.812 | 16.8 | 7.82 | 10.4 | **140** | 41.3 | 4.22 |
| 20 | 44.5 | 0.427 | n/d | 4.27 | 73.9 | 46.4 | 75.0 | 0.879 | 15.2 | 28.9 | 40.7 | 1.37 | 56.2 | 3.56 |
| 25 | 58.3 | 0.380 | n/d | 3.88 | 66.7 | 53.5 | 44.7 | 0.922 | **19.5** | 17.5 | 11.5 | 131 | 46.5 | 4.29 |
| 30 | 62.9 | 0.480 | n/d | 3.97 | 53.7 | 50.3 | 60.6 | 0.849 | 16.7 | 10.1 | 10.2 | 1.99 | 72.6 | 4.79 |
| 35 | 52.4 | 0.340 | n/d | 4.15 | 42.4 | 37.7 | 73.7 | 0.827 | 14.0 | 14.3 | 12.7 | 106 | 52.8 | 2.88 |
| 40 | 60.8 | 0.400 | n/d | 3.97 | 46.0 | 34.1 | 63.1 | 0.867 | 13.1 | 19.8 | 10.9 | 1.92 | 80.6 | 2.90 |
| 45 | 26.8 | 0.280 | n/d | 2.63 | 34.7 | 21.8 | 50.5 | 0.693 | 9.39 | 13.8 | 8.94 | 59.2 | 69.6 | 2.42 |
| 50 | **88.9** | **0.51** | n/d | 1.53 | **100** | 23.3 | 63.3 | **1.25** | 11.0 | **242** | 5.74 | 81.9 | **126** | 2.92 |
| 55 | 8.41 | 0.030 | n/d | 0.091 | 4.02 | 1.19 | 103 | 0.328 | 1.05 | 0.979 | 0.356 | 18.4 | 17.6 | 0.565 |
| 60 | 5.50 | 0.010 | n/d | 0.101 | 2.64 | 0.783 | 121 | 0.215 | 0.775 | 1.10 | 0.277 | 20.9 | 12.8 | 0.349 |
| 65 | 6.98 | 0.030 | n/d | 0.271 | 3.63 | 2.60 | 138 | 0.298 | 1.64 | 1.42 | 0.613 | 23.3 | 16.3 | 0.673 |
| 70 | 19.5 | 0.240 | n/d | 2.50 | 31.4 | 24.1 | 75.8 | 0.563 | 9.23 | 5.50 | 4.72 | 69.6 | 55.0 | 2.14 |
| 75 | 0.570 | 0.010 | n/d | 0.036 | 0.619 | 0.259 | 111 | 0.123 | 0.463 | 1.67 | 0.212 | 12.9 | 7.79 | 0.327 |
| 80 | 1.37 | 0.010 | n/d | 0.084 | 1.60 | 0.502 | 120 | 0.296 | 0.542 | 0.829 | 0.238 | 17.9 | 10.3 | 0.294 |
| 85 | 0.777 | n/d | n/d | 0.202 | 1.25 | 0.756 | 99.9 | 0.176 | 0.458 | 0.231 | 0.122 | 7.18 | 14.3 | 0.399 |
| 90 | 1.59 | 0.010 | n/d | 1.38 | 3.06 | 3.77 | **143** | 0.706 | 1.41 | 1.36 | 0.460 | 7.93 | 13.8 | 0.506 |
| 95 | n/a | n/a | n/a | n/a | n/a | n/a | n/a | n/a | n/a | n/a | n/a | n/a | n/a | n/a |
| 100 | n/a | n/a | n/a | n/a | n/a | n/a | n/a | n/a | n/a | n/a | n/a | n/a | n/a | n/a |
| 105 | n/a | n/a | n/a | n/a | n/a | n/a | n/a | n/a | n/a | n/a | n/a | n/a | n/a | n/a |
| 110 | n/a | n/a | n/a | n/a | n/a | n/a | n/a | n/a | n/a | n/a | n/a | n/a | n/a | n/a |
| 115 | n/a | n/a | n/a | n/a | n/a | n/a | n/a | n/a | n/a | n/a | n/a | n/a | n/a | n/a |
| 120 | n/a | n/a | n/a | n/a | n/a | n/a | n/a | n/a | n/a | n/a | n/a | n/a | n/a | n/a |
| 125 | n/a | n/a | n/a | n/a | n/a | n/a | n/a | n/a | n/a | n/a | n/a | n/a | n/a | n/a |
| 130 | n/a | n/a | n/a | n/a | n/a | n/a | n/a | n/a | n/a | n/a | n/a | n/a | n/a | n/a |
| 135 | n/a | n/a | n/a | n/a | n/a | n/a | n/a | n/a | n/a | n/a | n/a | n/a | n/a | n/a |
| 140 | n/a | n/a | n/a | n/a | n/a | n/a | n/a | n/a | n/a | n/a | n/a | n/a | n/a | n/a |
| 145 | n/a | n/a | n/a | n/a | n/a | n/a | n/a | n/a | n/a | n/a | n/a | n/a | n/a | n/a |
| 150 | n/a | n/a | n/a | n/a | n/a | n/a | n/a | n/a | n/a | n/a | n/a | n/a | n/a | n/a |
| 155 | n/a | n/a | n/a | n/a | n/a | n/a | n/a | n/a | n/a | n/a | n/a | n/a | n/a | n/a |
| 160 | n/a | n/a | n/a | n/a | n/a | n/a | n/a | n/a | n/a | n/a | n/a | n/a | n/a | n/a |
| 165 | n/a | n/a | n/a | n/a | n/a | n/a | n/a | n/a | n/a | n/a | n/a | n/a | n/a | n/a |
| 170 | n/a | n/a | n/a | n/a | n/a | n/a | n/a | n/a | n/a | n/a | n/a | n/a | n/a | n/a |
| 175 | n/a | n/a | n/a | n/a | n/a | n/a | n/a | n/a | n/a | n/a | n/a | n/a | n/a | n/a |
| 180 | n/a | n/a | n/a | n/a | n/a | n/a | n/a | n/a | n/a | n/a | n/a | n/a | n/a | n/a |
| 185 | n/a | n/a | n/a | n/a | n/a | n/a | n/a | n/a | n/a | n/a | n/a | n/a | n/a | n/a |
| 190 | n/a | n/a | n/a | n/a | n/a | n/a | n/a | n/a | n/a | n/a | n/a | n/a | n/a | n/a |
| 195 | n/a | n/a | n/a | n/a | n/a | n/a | n/a | n/a | n/a | n/a | n/a | n/a | n/a | n/a |
| 200 | n/a | n/a | n/a | n/a | n/a | n/a | n/a | n/a | n/a | n/a | n/a | n/a | n/a | n/a |
| min | 0.476 | n/d | n/d | 0.036 | 0.619 | 0.259 | 5.14 | 0.037 | 0.458 | 0.231 | 0.122 | 0.730 | 0.803 | 0.048 |
| max | 88.9 | 0.510 | n/d | 4.27 | 100 | 56.8 | 143 | 1.25 | 19.5 | 242 | 40.7 | 140 | 126 | 4.79 |
| median | 23.1 | 0.280 | n/d | 2.01 | 33.1 | 22.5 | 74.4 | 0.700 | 9.31 | 6.66 | 5.23 | 19.6 | 42.8 | 2.28 |
| mean | 29.4 | 0.236 | n/d | 2.12 | 30.9 | 22.9 | 80.0 | 0.598 | 8.36 | 21.3 | 7.43 | 44.1 | 41.0 | 2.10 |
| geomean | 11.5 | 0.116 | --- | 0.948 | 12.7 | 8.02 | 62.8 | 0.437 | 4.01 | 5.78 | 2.68 | 17.5 | 25.0 | 1.16 |

N/a = end of sediment core. N/d = Not detected. For statistical purposes half of the limit of detection was used for n/d samples. Bold indicate maximum concentration values
