# Supplementary material for: Assessing the ecological risk of heavy metal sediment contamination from Port Everglades Florida USA
Source: PeerJ. 2023 Nov 14;11:e16152. doi: 10.7717/peerj.16152 (PMC10655720; doi:10.7717/peerj.16152)
Supplement: Supplemental Information 7 — N/a = end of sediment core. N/d = Not detected. For statistical purposes half of the limit of detection was used for n/d samples. Bold indicate maximum concentration values. [file peerj-11-16152-s007.docx]

| **Table S6**. Dania Cut-off Canal 3 heavy metal concentrations (µg/g) by sediment core depth (cm), minimum (min), maximum (max), median, arithmetic mean (mean), and geometric mean (geomean). | | | | | | | | | | | | | | |
| --- | --- | --- | --- | --- | --- | --- | --- | --- | --- | --- | --- | --- | --- | --- |
| cm | Mo | Cd | Hg | Pb | V | Cr | Mn | Co | Ni | Zn | Cu | Sn | As | Se |
| 5 | 8.30 | 0.098 | n/d | **5.28** | 15.5 | 11.1 | 14.0 | 0.241 | 4.18 | 41.5 | 26.1 | 0.453 | 18.1 | 0.849 |
| 10 | 11.8 | 0.175 | n/d | 4.77 | 22.7 | 9.63 | 23.9 | 0.520 | 5.00 | 40.6 | **27.7** | 0.685 | 21.2 | 0.843 |
| 15 | 137 | 0.019 | n/d | 2.82 | **53.1** | **26.1** | 10.9 | 0.312 | **6.27** | 54.5 | 7.61 | 1.48 | 52.2 | 2.81 |
| 20 | 18.2 | **0.234** | n/d | 2.45 | 23.1 | 7.59 | 28.9 | 0.752 | 4.64 | 24.0 | 11.2 | 0.438 | 10.6 | 0.640 |
| 25 | 38.9 | 0.081 | n/d | 2.88 | 14.8 | 5.26 | 19.7 | 0.315 | 2.39 | 15.2 | 12.3 | 0.504 | 22.0 | 0.599 |
| 30 | 129 | 0.153 | n/d | 2.16 | 31.4 | 6.27 | 57.0 | 0.940 | 3.72 | 19.6 | 11.5 | 1.80 | 51.1 | 1.58 |
| 35 | 245 | 0.028 | n/d | 1.66 | 37.7 | 16.3 | 42.2 | 0.485 | 4.60 | 4.92 | 4.75 | 3.03 | 114 | 2.85 |
| 40 | 26.1 | 0.056 | n/d | 0.305 | 3.87 | 2.53 | **204** | **1.06** | 1.71 | **387** | 0.799 | 1.03 | 54.3 | 1.15 |
| 45 | **384** | 0.040 | n/d | 0.588 | 17.5 | 7.12 | 67.7 | 0.867 | 3.60 | 2.25 | 3.89 | 4.18 | **223** | **3.94** |
| 50 | 8.17 | 0.028 | n/d | 0.080 | 1.55 | 0.692 | 113 | 0.387 | 0.967 | 1.32 | 0.216 | 0.597 | 17.8 | 0.427 |
| 55 | 5.67 | 0.014 | n/d | 0.055 | 0.570 | 0.427 | 86.8 | 0.232 | 0.504 | 0.112 | 0.205 | 0.305 | 16.2 | 0.405 |
| 60 | 0.639 | 0.003 | n/d | 0.017 | 0.160 | 0.155 | 110 | 0.190 | 0.303 | 0.310 | 0.063 | 0.495 | 6.33 | 0.314 |
| 65 | 0.345 | 0.002 | n/d | 0.020 | 0.260 | 0.217 | 103 | 0.195 | 0.416 | 0.360 | 0.128 | 0.964 | 8.12 | 0.311 |
| 70 | 1.51 | 0.009 | n/d | 0.024 | 0.230 | 0.234 | 134 | 0.284 | 0.600 | 0.262 | 0.076 | 0.867 | 8.70 | 0.272 |
| 75 | 0.483 | n/d | n/d | 0.054 | 0.230 | 0.224 | 127 | 0.471 | 0.509 | 0.432 | n/d | 1.18 | 14.0 | 0.238 |
| 80 | 0.219 | 0.002 | n/d | 0.029 | 0.330 | 0.233 | 93.0 | 0.321 | 0.599 | 0.465 | 0.126 | 13.2 | 13.3 | 0.424 |
| 85 | 0.245 | 0.009 | n/d | 0.110 | 0.390 | 0.416 | 156 | 0.306 | 0.670 | 2.22 | 0.324 | 0.720 | 10.1 | 0.371 |
| 90 | 0.235 | 0.001 | n/d | 0.051 | 0.350 | 0.275 | 109 | 0.477 | 0.465 | 0.208 | 0.083 | 10.2 | 12.3 | 0.224 |
| 95 | 2.10 | 0.007 | n/d | 0.077 | 2.55 | 0.634 | 128 | 0.244 | 0.463 | 0.547 | 0.285 | 25.2 | 12.5 | 0.345 |
| 100 | 29.6 | 0.027 | n/d | 0.662 | 4.08 | 2.08 | 161 | 0.553 | 1.18 | 1.52 | 0.583 | **34.4** | 25.6 | 0.580 |
| 105 | n/a | n/a | n/a | n/a | n/a | n/a | n/a | n/a | n/a | n/a | n/a | n/a | n/a | n/a |
| 110 | n/a | n/a | n/a | n/a | n/a | n/a | n/a | n/a | n/a | n/a | n/a | n/a | n/a | n/a |
| 115 | n/a | n/a | n/a | n/a | n/a | n/a | n/a | n/a | n/a | n/a | n/a | n/a | n/a | n/a |
| 120 | n/a | n/a | n/a | n/a | n/a | n/a | n/a | n/a | n/a | n/a | n/a | n/a | n/a | n/a |
| 125 | n/a | n/a | n/a | n/a | n/a | n/a | n/a | n/a | n/a | n/a | n/a | n/a | n/a | n/a |
| 130 | n/a | n/a | n/a | n/a | n/a | n/a | n/a | n/a | n/a | n/a | n/a | n/a | n/a | n/a |
| 135 | n/a | n/a | n/a | n/a | n/a | n/a | n/a | n/a | n/a | n/a | n/a | n/a | n/a | n/a |
| 140 | n/a | n/a | n/a | n/a | n/a | n/a | n/a | n/a | n/a | n/a | n/a | n/a | n/a | n/a |
| 145 | n/a | n/a | n/a | n/a | n/a | n/a | n/a | n/a | n/a | n/a | n/a | n/a | n/a | n/a |
| 150 | n/a | n/a | n/a | n/a | n/a | n/a | n/a | n/a | n/a | n/a | n/a | n/a | n/a | n/a |
| 155 | n/a | n/a | n/a | n/a | n/a | n/a | n/a | n/a | n/a | n/a | n/a | n/a | n/a | n/a |
| 160 | n/a | n/a | n/a | n/a | n/a | n/a | n/a | n/a | n/a | n/a | n/a | n/a | n/a | n/a |
| 165 | n/a | n/a | n/a | n/a | n/a | n/a | n/a | n/a | n/a | n/a | n/a | n/a | n/a | n/a |
| 170 | n/a | n/a | n/a | n/a | n/a | n/a | n/a | n/a | n/a | n/a | n/a | n/a | n/a | n/a |
| 175 | n/a | n/a | n/a | n/a | n/a | n/a | n/a | n/a | n/a | n/a | n/a | n/a | n/a | n/a |
| 180 | n/a | n/a | n/a | n/a | n/a | n/a | n/a | n/a | n/a | n/a | n/a | n/a | n/a | n/a |
| 185 | n/a | n/a | n/a | n/a | n/a | n/a | n/a | n/a | n/a | n/a | n/a | n/a | n/a | n/a |
| 190 | n/a | n/a | n/a | n/a | n/a | n/a | n/a | n/a | n/a | n/a | n/a | n/a | n/a | n/a |
| 195 | n/a | n/a | n/a | n/a | n/a | n/a | n/a | n/a | n/a | n/a | n/a | n/a | n/a | n/a |
| 200 | n/a | n/a | n/a | n/a | n/a | n/a | n/a | n/a | n/a | n/a | n/a | n/a | n/a | n/a |
| min | 0.219 | n/d | 0.00 | 0.017 | 0.160 | 0.155 | 10.9 | 0.190 | 0.303 | 0.112 | n/d | 0.305 | 6.33 | 0.224 |
| max | 384 | 0.234 | 0.00 | 5.28 | 53.1 | 26.1 | 204 | 1.06 | 6.27 | 387 | 27.7 | 34.4 | 223 | 3.94 |
| median | 8.23 | 0.027 | n/d | 0.208 | 3.21 | 1.39 | 97.8 | 0.354 | 1.07 | 1.87 | 0.583 | 0.996 | 17.0 | 0.503 |
| mean | 52.4 | 0.052 | n/d | 1.20 | 11.5 | 4.88 | 89.4 | 0.458 | 2.14 | 29.9 | 5.68 | 5.09 | 35.6 | 0.959 |
| geomean | 7.36 | 0.023 | --- | 0.289 | 2.92 | 1.62 | 67.6 | 0.404 | 1.35 | 3.27 | 1.27 | 1.77 | 22.5 | 0.663 |

N/a = end of sediment core. N/d = Not detected. For statistical purposes half of the limit of detection was used for n/d samples. Bold indicate maximum concentration values.
