# Supplementary material for: Assessing the ecological risk of heavy metal sediment contamination from Port Everglades Florida USA
Source: PeerJ. 2023 Nov 14;11:e16152. doi: 10.7717/peerj.16152 (PMC10655720; doi:10.7717/peerj.16152)
Supplement: Supplemental Information 8 — N/a = end of sediment core. N/d = Not detected. For statistical purposes half of the limit of detection was used for n/d samples. Bold indicate maximum concentration values. [file peerj-11-16152-s008.docx]

| Table S7. Park Education Center 1 heavy metal concentrations (µg/g) by sediment core depth (cm), minimum (min), maximum (max), median, arithmetic mean (mean), and geometric mean (geomean). | | | | | | | | | | | | | | |
| --- | --- | --- | --- | --- | --- | --- | --- | --- | --- | --- | --- | --- | --- | --- |
| cm | Mo | Cd | Hg | Pb | V | Cr | Mn | Co | Ni | Zn | Cu | Sn | As | Se |
| 5 | 0.065 | 0.027 | 0.0 | 3.22 | 3.14 | 5.30 | 11.2 | 0.139 | 1.25 | 17.3 | 12.5 | 0.110 | 1.34 | 0.125 |
| 10 | 0.125 | 0.028 | 0.0 | 1.60 | 2.34 | 4.22 | 5.83 | 0.096 | 0.696 | 8.60 | 5.00 | 0.046 | 0.641 | 0.059 |
| 15 | 0.743 | 0.052 | 0.0 | 3.61 | 6.11 | 6.29 | 7.89 | 0.145 | 1.94 | 25.7 | 15.7 | 0.130 | 2.01 | 0.155 |
| 20 | 0.206 | 0.032 | 0.0 | 1.29 | 2.82 | 3.55 | 5.56 | 0.100 | 0.725 | 10.6 | 3.78 | 0.035 | 0.775 | 0.063 |
| 25 | 1.04 | 0.048 | 0.0 | 3.87 | 10.1 | 7.93 | 9.69 | 0.225 | 2.84 | 17.2 | 13.2 | 0.122 | 2.81 | 0.171 |
| 30 | 1.18 | 0.032 | 0.0 | 1.92 | 9.81 | 9.74 | 10.4 | 0.288 | 2.38 | 6.88 | 5.05 | 0.063 | 3.93 | 0.269 |
| 35 | 1.20 | 0.046 | 0.0 | 2.06 | 11.5 | 16.6 | 17.2 | 0.447 | 3.00 | 2.80 | 1.89 | n/d | 2.94 | 0.315 |
| 40 | 1.51 | 0.052 | 0.01 | 4.16 | 10.5 | 16.0 | 22.0 | 0.532 | 3.60 | 5.19 | 3.60 | 0.051 | 4.54 | 0.299 |
| 45 | 0.444 | 0.026 | 0.0 | 1.50 | 7.00 | 12.1 | 11.8 | 0.334 | 2.06 | 3.65 | 1.12 | n/d | 2.97 | 0.344 |
| 50 | 0.965 | 0.051 | 0.0 | 2.76 | 10.1 | 13.2 | 20.3 | 0.532 | 3.14 | 9.83 | 2.29 | 0.010 | 4.42 | 0.328 |
| 55 | 3.14 | 0.344 | 0.36 | **20.1** | **55.0** | 14.4 | 30.4 | 0.747 | **16.4** | **33.3** | **28.1** | 0.400 | 12.1 | 0.614 |
| 60 | 3.86 | 0.334 | 0.25 | 13.7 | 34.2 | 11.3 | 23.6 | 0.536 | 10.4 | 29.9 | 22.7 | 0.114 | 12.1 | 0.646 |
| 65 | 2.85 | 0.220 | 0.29 | 10.3 | 16.0 | 6.70 | 14.5 | 0.284 | 4.53 | 19.0 | 15.2 | 0.220 | 6.16 | 0.379 |
| 70 | 2.79 | 0.242 | 0.16 | 8.50 | 11.6 | 6.13 | 15.8 | 0.233 | 2.96 | 16.2 | 10.5 | 0.189 | 5.62 | 0.275 |
| 75 | 0.886 | 0.094 | 0.02 | 3.39 | 3.47 | 2.43 | 7.36 | 0.093 | 1.15 | 6.66 | 3.56 | 0.036 | 2.10 | 0.090 |
| 80 | 0.808 | 0.027 | 0.0 | 1.21 | 1.28 | 1.30 | 4.79 | 0.055 | 0.399 | 3.10 | 1.05 | 0.017 | 1.13 | 0.002 |
| 85 | 1.98 | 0.029 | 0.01 | 2.40 | 4.50 | 2.88 | 11.3 | 0.134 | 0.897 | 6.22 | 3.23 | 0.072 | 3.24 | 0.155 |
| 90 | 0.484 | 0.011 | 0.072 | 0.971 | 0.654 | 1.23 | 5.34 | 0.048 | 0.567 | 4.07 | 0.879 | 0.006 | 0.850 | 0.014 |
| 95 | 5.14 | 0.038 | 0.0 | 4.46 | 7.10 | 4.98 | 22.2 | 0.273 | 1.63 | 8.37 | 6.58 | 0.283 | 5.76 | 0.280 |
| 100 | 9.19 | 0.13 | 0.0 | 9.01 | 24.0 | 12.0 | 34.2 | 0.496 | 5.05 | 21.3 | 15.5 | 0.349 | 11.1 | 0.759 |
| 105 | 1.32 | 0.02 | 0.0 | 2.61 | 3.72 | 2.96 | 12.4 | 0.138 | 1.01 | 4.30 | 3.85 | 0.045 | 2.80 | 0.147 |
| 110 | 3.93 | 0.06 | 0.0 | 5.38 | 10.3 | 5.84 | 20.5 | 0.233 | 3.12 | 13.4 | 3.89 | 0.371 | 6.99 | 0.476 |
| 115 | 4.37 | 0.088 | **0.736** | 3.74 | 15.0 | 9.44 | 35.8 | 0.366 | 3.68 | 10.5 | 4.13 | 0.189 | 11.6 | 0.817 |
| 120 | 8.14 | 0.1 | n/d | 3.52 | 15.9 | 7.53 | 26.8 | 0.360 | 2.79 | 8.59 | 3.11 | 0.183 | 15.4 | 0.590 |
| 125 | 8.92 | 0.079 | n/d | 5.66 | 15.2 | 8.45 | 32.2 | 0.305 | 3.27 | 9.06 | 3.78 | 0.055 | 13.5 | 0.767 |
| 130 | **43.8** | **0.916** | n/d | 3.99 | 50.7 | **21.0** | 20.4 | 0.674 | 11.3 | 6.79 | 4.97 | n/d | **32.1** | **2.26** |
| 135 | 14.1 | 0.11 | n/d | 3.98 | 15.3 | 6.11 | 23.3 | 0.328 | 2.81 | 5.26 | 2.84 | 0.165 | 15.8 | 0.666 |
| 140 | 2.91 | 0.03 | n/d | 2.29 | 7.20 | 4.16 | 18.1 | 0.255 | 1.51 | 4.73 | 1.65 | 0.122 | 7.32 | 0.321 |
| 145 | 2.72 | 0.03 | 0.003 | 1.81 | 5.24 | 3.42 | 17.4 | 0.143 | 0.990 | 3.58 | 1.31 | 0.086 | 3.82 | 0.231 |
| 150 | 0.789 | 0.01 | 0.005 | 1.28 | 3.67 | 2.75 | 6.89 | 0.162 | 0.604 | 1.55 | 0.729 | 0.004 | 1.49 | 0.155 |
| 155 | 0.772 | 0.01 | 0.055 | 3.39 | 8.76 | 7.52 | 9.71 | 0.517 | 1.67 | 1.06 | 2.09 | 0.104 | 0.880 | 0.347 |
| 160 | 2.28 | 0.044 | 0.116 | 2.10 | 33.4 | 7.64 | 30.5 | 0.752 | 2.45 | 2.81 | 2.03 | 0.375 | 4.01 | 0.408 |
| 165 | 1.50 | 0.04 | 0.048 | 1.89 | 22.3 | 5.64 | **47.1** | **0.882** | 2.47 | 2.60 | 3.66 | 0.144 | 2.87 | 0.290 |
| 170 | 1.21 | 0.03 | n/d | 1.78 | 27.7 | 5.85 | 41.1 | 0.668 | 2.76 | 11.3 | 2.92 | **12.2** | 2.05 | 0.145 |
| 175 | 0.425 | 0.03 | n/d | 1.39 | 9.92 | 4.45 | 27.3 | 0.419 | 1.63 | 1.24 | 1.55 | 0.558 | 1.11 | 0.122 |
| 180 | 0.336 | 0.02 | n/d | 1.49 | 8.77 | 4.86 | 28.5 | 0.392 | 1.77 | 2.35 | 1.34 | 0.168 | 0.937 | 0.099 |
| 185 | n/a | n/a | n/a | n/a | n/a | n/a | n/a | n/a | n/a | n/a | n/a | n/a | n/a | n/a |
| 190 | n/a | n/a | n/a | n/a | n/a | n/a | n/a | n/a | n/a | n/a | n/a | n/a | n/a | n/a |
| 195 | n/a | n/a | n/a | n/a | n/a | n/a | n/a | n/a | n/a | n/a | n/a | n/a | n/a | n/a |
| 200 | n/a | n/a | n/a | n/a | n/a | n/a | n/a | n/a | n/a | n/a | n/a | n/a | n/a | n/a |
| min | 0.065 | 0.011 | n/d | 0.971 | 0.654 | 1.23 | 4.79 | 0.048 | 0.399 | 1.06 | 0.729 | 0.004 | 0.641 | 0.002 |
| max | 43.8 | 0.916 | 0.736 | 20.1 | 55.0 | 21.0 | 47.1 | 0.882 | 16.4 | 33.3 | 28.1 | 12.2 | 32.1 | 2.26 |
| median | 1.41 | 0.041 | 0.002 | 2.99 | 9.99 | 6.12 | 17.8 | 0.296 | 2.42 | 6.84 | 3.63 | 0.122 | 3.53 | 0.285 |
| mean | 3.78 | 0.096 | 0.077 | 4.07 | 13.5 | 7.38 | 19.2 | 0.343 | 3.04 | 9.58 | 5.98 | 0.516 | 5.81 | 0.366 |
| geomean | 1.60 | 0.054 | 0.00038 | 3.12 | 8.90 | 6.05 | 16.1 | 0.270 | 2.17 | 6.79 | 3.87 | 0.115 | 3.69 | 0.215 |

N/a = end of sediment core. N/d = Not detected. For statistical purposes half of the limit of detection was used for n/d samples. Bold indicate maximum concentration values.
