# Supplementary material for: Assessing the ecological risk of heavy metal sediment contamination from Port Everglades Florida USA
Source: PeerJ. 2023 Nov 14;11:e16152. doi: 10.7717/peerj.16152 (PMC10655720; doi:10.7717/peerj.16152)
Supplement: Supplemental Information 9 — N/a = end of sediment core. N/d = Not detected. For statistical purposes half of the limit of detection was used for n/d samples. Bold indicate maximum concentration values. [file peerj-11-16152-s009.docx]

| Table S8. Park Education Center 2 heavy metal concentrations (µg/g) by sediment core depth (cm), minimum (min), maximum (max), median, arithmetic mean (mean), and geometric mean (geomean). | | | | | | | | | | | | | | |
| --- | --- | --- | --- | --- | --- | --- | --- | --- | --- | --- | --- | --- | --- | --- |
| cm | Mo | Cd | Hg | Pb | V | Cr | Mn | Co | Ni | Zn | Cu | Sn | As | Se |
| 5 | 4.94 | **0.772** | 0.238 | **73.8** | 59.0 | **46.6** | **73.3** | **2.22** | **29.3** | **416** | **215** | **3.27** | 18.9 | 1.35 |
| 10 | 0.234 | 0.053 | n/d | 4.82 | 5.16 | 7.78 | 14.9 | 0.248 | 2.20 | 25.7 | 22.2 | 0.572 | 1.56 | 0.161 |
| 15 | 0.114 | 0.030 | n/d | 1.84 | 2.24 | 4.30 | 6.63 | 0.112 | 0.990 | 12.3 | 4.76 | 1.12 | 0.61 | 0.100 |
| 20 | 0.104 | 0.041 | n/d | 1.37 | 2.41 | 3.39 | 6.45 | 0.131 | 0.800 | 6.23 | 2.96 | 0.687 | 0.753 | 0.090 |
| 25 | 1.08 | 0.064 | n/d | 3.64 | 13.8 | 9.60 | 11.6 | 0.320 | 3.65 | 9.81 | 7.22 | 1.08 | 2.61 | 0.224 |
| 30 | 1.37 | 0.074 | n/d | 2.45 | 12.0 | 12.9 | 18.9 | 0.389 | 3.34 | 7.55 | 5.81 | 0.462 | 3.21 | 0.199 |
| 35 | 1.37 | 0.055 | n/d | 2.42 | 12.0 | 19.7 | 17.0 | 0.527 | 3.40 | 2.79 | 1.80 | 0.302 | 2.45 | 0.230 |
| 40 | 0.690 | 0.055 | n/d | 2.17 | 11.7 | 18.9 | 16.7 | 0.435 | 3.28 | 3.35 | 1.55 | 0.541 | 2.18 | 0.130 |
| 45 | 1.50 | 0.053 | n/d | 3.47 | 10.8 | 17.6 | 24.6 | 0.579 | 3.41 | 3.88 | 2.38 | 0.293 | 4.64 | 0.357 |
| 50 | 2.06 | 0.073 | n/d | 3.67 | 11.5 | 18.9 | 23.6 | 0.591 | 4.19 | 6.11 | 3.42 | 0.540 | 4.21 | 0.137 |
| 55 | 1.68 | 0.046 | n/d | 2.63 | 12.3 | 17.9 | 18.6 | 0.543 | 3.20 | 2.81 | 1.83 | 0.328 | 3.43 | 0.306 |
| 60 | 1.05 | 0.033 | n/d | 2.06 | 7.81 | 11.8 | 14.0 | 0.367 | 2.56 | 4.33 | 1.62 | 0.222 | 3.59 | 0.348 |
| 65 | 0.976 | 0.054 | 0.009 | 2.62 | 10.8 | 16.4 | 18.3 | 0.543 | 3.14 | 3.05 | 2.06 | 0.255 | 3.29 | 0.297 |
| 70 | 3.29 | 0.202 | 0.260 | 9.56 | 34.2 | 13.4 | 25.3 | 0.589 | 10.6 | 16.6 | 13.8 | 1.02 | 8.94 | 0.613 |
| 75 | 5.50 | 0.334 | 0.332 | 14.0 | 44.5 | 11.4 | 21.6 | 0.586 | 12.1 | 25.0 | 24.1 | 1.10 | 10.4 | 0.616 |
| 80 | 7.27 | 0.326 | **0.632** | 18.0 | 31.4 | 10.3 | 22.5 | 0.461 | 10.1 | 28.2 | 25.8 | 1.10 | 8.85 | 0.623 |
| 85 | 4.09 | 0.275 | 0.205 | 10.1 | 15.2 | 7.17 | 16.4 | 0.276 | 3.77 | 18.5 | 15.5 | 0.668 | 8.08 | 0.530 |
| 90 | 8.71 | 0.436 | 0.488 | 15.6 | 15.4 | 9.15 | 26.1 | 0.363 | 3.72 | 25.6 | 16.0 | 0.881 | 8.24 | 0.602 |
| 95 | 0.548 | 0.069 | n/d | 17.6 | 1.01 | 1.84 | 12.7 | 0.104 | 0.614 | 7.38 | 2.64 | 1.12 | 2.19 | 0.116 |
| 100 | 0.512 | 0.036 | n/d | 1.79 | 1.23 | 1.39 | 6.57 | 0.059 | 0.423 | 3.97 | 1.40 | 0.702 | 1.09 | 0.028 |
| 105 | 0.597 | 0.025 | n/d | 1.31 | 0.944 | 1.39 | 5.73 | 0.058 | 0.423 | 4.26 | 1.22 | 1.12 | 0.804 | 0.053 |
| 110 | 4.17 | 0.041 | 0.015 | 5.37 | 14.7 | 5.71 | 31.4 | 0.280 | 1.70 | 8.98 | 9.39 | 0.429 | 5.10 | 0.277 |
| 115 | 6.33 | 0.071 | 0.021 | 3.25 | 81.1 | 7.43 | 30.7 | 0.253 | 1.94 | 14.6 | 3.65 | 1.19 | 11.5 | 0.525 |
| 120 | 6.64 | 0.062 | n/d | 2.88 | 63.8 | 7.36 | 28.1 | 0.234 | 1.97 | 6.49 | 3.45 | 0.782 | 8.09 | 0.512 |
| 125 | 8.30 | 0.078 | n/d | 7.80 | 49.4 | 10.1 | 41.9 | 0.454 | 3.25 | 9.83 | 6.89 | 1.17 | 10.6 | 0.716 |
| 130 | **89.8** | 0.237 | n/d | 1.60 | 106 | 13.1 | 53.4 | 2.06 | 4.93 | 4.04 | 2.41 | 3.19 | **62.4** | **1.59** |
| 135 | 30.2 | 0.102 | 0.075 | 3.46 | **176** | 16.8 | 48.9 | 0.516 | 5.13 | 6.65 | 3.05 | 2.44 | 22.0 | 1.39 |
| 140 | 6.99 | 0.065 | n/d | 6.57 | 110 | 10.1 | 45.6 | 0.366 | 2.82 | 7.37 | 4.04 | 0.824 | 8.27 | 0.548 |
| 145 | 3.13 | 0.061 | n/d | 7.99 | 15.7 | 7.87 | 32.3 | 0.354 | 2.45 | 10.0 | 5.24 | 0.768 | 6.75 | 0.438 |
| 150 | 8.70 | 0.084 | n/d | 4.25 | 43.6 | 10.7 | 30.9 | 0.369 | 2.96 | 6.13 | 4.09 | 0.802 | 12.2 | 0.732 |
| 155 | 0.475 | 0.011 | n/d | 0.275 | 0.927 | 0.991 | 2.72 | 0.031 | 0.395 | 0.608 | 0.054 | 0.548 | 1.25 | 0.056 |
| 160 | 0.242 | 0.005 | n/d | 0.239 | 0.704 | 0.669 | 1.96 | 0.030 | 0.232 | 0.709 | 0.004 | 0.495 | 0.799 | n/d |
| 165 | 0.769 | 0.013 | n/d | 0.436 | 1.80 | 1.34 | 4.23 | 0.056 | 0.443 | 0.818 | 0.250 | 0.133 | 1.45 | 0.049 |
| 170 | 2.65 | 0.025 | 0.031 | 1.03 | 9.88 | 2.93 | 18.1 | 0.328 | 1.16 | 1.20 | 1.10 | 0.282 | 3.35 | 0.104 |
| 175 | 1.74 | 0.044 | n/d | 1.67 | 15.9 | 5.44 | 27.4 | 0.450 | 1.92 | 2.91 | 2.34 | n/d | 3.86 | 0.306 |
| 180 | n/a | n/a | n/a | n/a | n/a | n/a | n/a | n/a | n/a | n/a | n/a | n/a | n/a | n/a |
| 185 | n/a | n/a | n/a | n/a | n/a | n/a | n/a | n/a | n/a | n/a | n/a | n/a | n/a | n/a |
| 190 | n/a | n/a | n/a | n/a | n/a | n/a | n/a | n/a | n/a | n/a | n/a | n/a | n/a | n/a |
| 195 | n/a | n/a | n/a | n/a | n/a | n/a | n/a | n/a | n/a | n/a | n/a | n/a | n/a | n/a |
| 200 | n/a | n/a | n/a | n/a | n/a | n/a | n/a | n/a | n/a | n/a | n/a | n/a | n/a | n/a |
| min | 0.104 | 0.005 | n/d | 0.239 | 0.704 | 0.669 | 1.96 | 0.030 | 0.232 | 0.608 | n/d | 0.133 | 0.607 | 0.028 |
| max | 89.8 | 0.772 | 0.632 | 73.8 | 176 | 46.6 | 73.3 | 2.22 | 29.3 | 416 | 215 | 3.27 | 62.4 | 1.59 |
| median | 1.74 | 0.061 | 0.205 | 3.25 | 12.3 | 9.60 | 18.9 | 0.366 | 2.96 | 6.49 | 3.42 | 0.735 | 3.86 | 0.306 |
| mean | 6.22 | 0.114 | 0.209 | 6.90 | 28.7 | 10.4 | 22.8 | 0.437 | 3.90 | 20.4 | 12.0 | 0.895 | 7.36 | 0.422 |
| geomean | 1.98 | 0.0653 | 0.0986 | 3.37 | 12.0 | 7.17 | 17.5 | 0.289 | 2.31 | 6.58 | 3.15 | 0.694 | 4.15 | 0.276 |

N/a = end of sediment core. N/d = Not detected. For statistical purposes half of the limit of detection was used for n/d samples. Bold indicate maximum concentration values.
