# Supplementary material for: Assessing the ecological risk of heavy metal sediment contamination from Port Everglades Florida USA
Source: PeerJ. 2023 Nov 14;11:e16152. doi: 10.7717/peerj.16152 (PMC10655720; doi:10.7717/peerj.16152)
Supplement: Supplemental Information 10 — N/a = end of sediment core. N/d = Not detected. For statistical purposes half of the limit of detection was used for n/d samples. Bold indicate maximum concentration values. [file peerj-11-16152-s010.docx]

| **Table S9**. Park Education Center 3 heavy metal concentrations (µg/g) by sediment core depth (cm), minimum (min), maximum (max), median, arithmetic mean (mean), and geometric mean (geomean). | | | | | | | | | | | | | | | |
| --- | --- | --- | --- | --- | --- | --- | --- | --- | --- | --- | --- | --- | --- | --- | --- |
| cm | Mo | Cd | Hg | Pb | V | Cr | Mn | Co | Ni | Zn | Cu | Sn | As | Se |  |
| 5 | 10.1 | **0.395** | 0.252 | **35.9** | 100 | **41.1** | **98.1** | 1.23 | **22.2** | 225 | **210** | 14.6 | 22.2 | 1.25 |  |
| 10 | 1.50 | 0.077 | n/d | 4.24 | 20.2 | 13.4 | 18.8 | 0.399 | 5.00 | 14.6 | 10.5 | 11.7 | 3.75 | 0.242 |  |
| 15 | 0.260 | 0.037 | n/d | 1.65 | 5.90 | 5.41 | 8.06 | 0.177 | 1.67 | 5.61 | 3.16 | 9.83 | 1.23 | 0.050 |  |
| 20 | 0.136 | 0.031 | n/d | 1.73 | 4.25 | 4.17 | 5.84 | 0.128 | 1.13 | 7.11 | 4.61 | **15.6** | 1.00 | n/d |  |
| 25 | 0.122 | 0.035 | n/d | 1.69 | 4.34 | 4.31 | 5.75 | 0.116 | 1.16 | 7.50 | 4.81 | 11.7 | 1.04 | 0.045 |  |
| 30 | 1.28 | 0.068 | n/d | 3.75 | 15.3 | 11.4 | 13.3 | 0.354 | 3.99 | 15.7 | 11.3 | 8.68 | 3.56 | 0.275 |  |
| 35 | 0.193 | 0.034 | n/d | 1.59 | 4.59 | 4.44 | 6.47 | 0.125 | 1.31 | **603** | 4.36 | 11.0 | 0.979 | 0.581 |  |
| 40 | 2.08 | 0.050 | n/d | 2.31 | 11.7 | 13.2 | 15.3 | 0.450 | 2.92 | 4.05 | 3.11 | 4.06 | 4.12 | 0.281 |  |
| 45 | 0.588 | 0.033 | n/d | 1.00 | 6.13 | 11.0 | 11.2 | 0.296 | 1.74 | 10.8 | 0.792 | 2.74 | 2.25 | 0.303 |  |
| 50 | 1.03 | 0.029 | n/d | 1.57 | 9.66 | 13.8 | 13.6 | 0.408 | 2.36 | 1.60 | 1.43 | 2.23 | 3.78 | 0.502 |  |
| 55 | 1.30 | 0.048 | n/d | 2.16 | 10.4 | 14.4 | 16.1 | 0.394 | 2.60 | 1.60 | 1.42 | 2.71 | 2.49 | 0.307 |  |
| 60 | 1.27 | 0.046 | n/d | 3.01 | 10.8 | 19.4 | 25.5 | 0.551 | 3.36 | 3.05 | 2.01 | 4.34 | 3.78 | 0.34 |  |
| 65 | 1.83 | 0.075 | 0.114 | 6.91 | 13.0 | 19.9 | 27.7 | 0.624 | 4.60 | 14.3 | 6.95 | 4.00 | 4.33 | 0.478 |  |
| 70 | 1.90 | 0.068 | n/d | 3.08 | 10.9 | 17.1 | 22.3 | 0.574 | 3.45 | 3.01 | 2.74 | 3.11 | 3.97 | 0.346 |  |
| 75 | 2.01 | 0.049 | n/d | 2.35 | 10.5 | 14.5 | 16.8 | 0.502 | 3.02 | 2.79 | 1.94 | 9.88 | 3.09 | 0.233 |  |
| 80 | 1.77 | 0.058 | n/d | 3.28 | 12.1 | 18.9 | 18.7 | 0.551 | 3.24 | 3.18 | 1.97 | 3.80 | 3.32 | 0.325 |  |
| 85 | 2.42 | 0.052 | n/d | 3.09 | 11.5 | 16.2 | 20.5 | 0.560 | 3.35 | 2.89 | 2.34 | 4.31 | 4.01 | 0.388 |  |
| 90 | 3.76 | 0.063 | n/d | 3.64 | 17.2 | 19.7 | 26.1 | 0.899 | 5.37 | 3.49 | 2.85 | 7.37 | 6.55 | 0.588 |  |
| 95 | 4.24 | 0.062 | n/d | 3.54 | 15.1 | 17.1 | 26.7 | 0.908 | 4.02 | 4.20 | 2.54 | 8.89 | 6.60 | 0.400 |  |
| 100 | 4.51 | 0.106 | 0.115 | 5.11 | 28.6 | 13.5 | 21.2 | 0.567 | 6.88 | 5.69 | 6.29 | 6.73 | 8.32 | 0.473 |  |
| 105 | 7.17 | 0.246 | 0.290 | 13.2 | 38.8 | 12.0 | 21.3 | 0.527 | 10.1 | 23.1 | 22.4 | 5.96 | 14.4 | 0.757 |  |
| 110 | 6.65 | 0.286 | 0.202 | 10.1 | 16.6 | 7.55 | 15.4 | 0.292 | 4.10 | 19.2 | 13.7 | 5.72 | 7.14 | 0.470 |  |
| 115 | 0.750 | 0.046 | n/d | 2.15 | 2.32 | 1.77 | 6.02 | 0.058 | 0.654 | 3.82 | 2.18 | 8.22 | 1.50 | 0.062 |  |
| 120 | 4.22 | 0.122 | 0.139 | 4.21 | 7.53 | 4.07 | 12.0 | 0.181 | 1.53 | 10.4 | 4.45 | 6.33 | 5.88 | 0.264 |  |
| 125 | 8.33 | 0.126 | **0.466** | 6.73 | 16.3 | 7.50 | 26.1 | 0.334 | 3.01 | 22.6 | 8.82 | 6.17 | 12.0 | 0.502 |  |
| 130 | 0.793 | 0.019 | n/d | 3.14 | 1.17 | 1.65 | 5.73 | 0.057 | 0.450 | 5.60 | 1.29 | 7.34 | 1.21 | 0.003 |  |
| 135 | 2.04 | 0.039 | n/d | 2.70 | 10.7 | 3.72 | 15.3 | 0.136 | 1.21 | 4.31 | 2.65 | 6.51 | 3.34 | 0.254 |  |
| 140 | 10.2 | 0.102 | n/d | 5.49 | 37.4 | 9.90 | 28.8 | 0.380 | 3.06 | 8.02 | 5.10 | 10.5 | 10.9 | 0.923 |  |
| 145 | **30.1** | 0.162 | n/d | 4.55 | 80.7 | 16.1 | 59.2 | 0.516 | 4.68 | 9.23 | 4.64 | 15.6 | 27.8 | 1.27 |  |
| 150 | 2.37 | 0.046 | 0.045 | 6.57 | 9.14 | 5.77 | 24.2 | 0.298 | 2.14 | 8.06 | 4.01 | 0.574 | 5.29 | 0.39 |  |
| 155 | 0.458 | 0.009 | n/d | 1.21 | 1.67 | 1.73 | 7.63 | 0.061 | 0.495 | 1.49 | 0.671 | 0.857 | 1.41 | n/d |  |
| 160 | 15.6 | 0.069 | n/d | 1.91 | 38.2 | 14.5 | 17.0 | 2.06 | 17.0 | 4.95 | 5.02 | 1.72 | 18.9 | 1.09 |  |
| 165 | 13.8 | 0.084 | n/d | 1.87 | 45.7 | 15.7 | 11.2 | 3.52 | 17.0 | 1.50 | 5.03 | 1.18 | 15.4 | 1.23 |  |
| 170 | 7.84 | 0.096 | n/d | 3.07 | 41.6 | 15.8 | 10.6 | 2.41 | 13.5 | 1.89 | 4.76 | 0.121 | 10.5 | 1.16 |  |
| 175 | 6.81 | 0.085 | 0.007 | 3.24 | 39.7 | 13.3 | 8.60 | 1.45 | 9.57 | 3.03 | 4.05 | 0.227 | 7.43 | 1.10 |  |
| 180 | 8.09 | 0.072 | n/d | 3.19 | 46.2 | 16.1 | 10.1 | 2.57 | 13.3 | 4.26 | 4.18 | 0.100 | 10.7 | 1.13 |  |
| 185 | 6.99 | 0.089 | n/d | 3.84 | 41.7 | 16.1 | 10.1 | 2.13 | 14.7 | 2.60 | 4.07 | 0.215 | 9.72 | 0.940 |  |
| 190 | 22.1 | 0.220 | n/d | 4.78 | **124** | 32.7 | 15.2 | **6.84** | 20.0 | 1.91 | 3.69 | 0.497 | **31.5** | **1.67** |  |
| 195 | 8.32 | 0.048 | n/d | 2.12 | 24.2 | 6.39 | 21.0 | 1.06 | 3.69 | 1.76 | 1.92 | 0.264 | 6.81 | 0.347 |  |
| 200 | 0.426 | 0.008 | n/d | 1.51 | 4.94 | 3.42 | 16.6 | 0.352 | 1.28 | 2.02 | 1.07 | 0.487 | 1.14 | 0.108 |  |
| min | 0.122 | 0.008 | n/d | 1.00 | 1.17 | 1.65 | 5.73 | 0.057 | 0.450 | 1.49 | 0.671 | 0.100 | 0.979 | n/d |  |
| max | 30.1 | 0.395 | 0.466 | 35.9 | 124 | 41.1 | 98.1 | 6.84 | 22.2 | 603 | 210 | 15.6 | 31.5 | 1.67 |  |
| median | 2.23 | 0.062 | 0.139 | 3.11 | 12.5 | 13.3 | 15.7 | 0.476 | 3.35 | 4.29 | 4.03 | 5.03 | 4.22 | 0.397 |  |
| mean | 5.13 | 0.085 | 0.181 | 4.43 | 23.8 | 12.5 | 19.0 | 0.876 | 5.62 | 27.0 | 9.73 | 5.65 | 7.33 | 0.555 |  |
| geomean | 2.43 | 0.0623 | 0.116 | 3.25 | 14.3 | 9.80 | 15.5 | 0.472 | 3.52 | 5.92 | 3.83 | 3.05 | 4.80 | 0.368 |  |

N/a = end of sediment core. N/d = Not detected. For statistical purposes half of the limit of detection was used for n/d samples. Bold indicate maximum concentration values.
