# Supplementary material for: Assessing the ecological risk of heavy metal sediment contamination from Port Everglades Florida USA
Source: PeerJ. 2023 Nov 14;11:e16152. doi: 10.7717/peerj.16152 (PMC10655720; doi:10.7717/peerj.16152)
Supplement: Supplemental Information 11 — N/a = end of sediment core. N/d = Not detected. For statistical purposes half of the limit of detection was used for n/d samples. Bold indicate maximum concentration values. [file peerj-11-16152-s011.docx]

| Table S10. Park Headquarters 1 heavy metal concentrations (µg/g) by sediment core depth (cm), minimum (min), maximum (max), median, arithmetic mean (mean), and geometric mean (geomean). | | | | | | | | | | | | | | |
| --- | --- | --- | --- | --- | --- | --- | --- | --- | --- | --- | --- | --- | --- | --- |
| cm | Mo | Cd | Hg | Pb | V | Cr | Mn | Co | Ni | Zn | Cu | Sn | As | Se |
| 5 | 0.394 | 0.015 | n/d | 1.50 | 1.55 | 2.53 | 7.86 | 0.051 | 0.360 | 6.65 | 2.77 | 0.734 | 0.958 | 0.015 |
| 10 | 3.42 | 0.039 | n/d | 1.46 | 4.34 | 4.09 | 10.6 | 0.133 | 1.23 | 75.5 | 2.97 | 0.820 | 2.86 | 0.239 |
| 15 | 40.4 | 0.101 | n/d | 2.20 | 36.1 | 13.3 | 11.4 | 0.166 | 4.73 | 16.5 | 6.96 | 3.15 | 11.3 | 0.952 |
| 20 | 45.0 | 0.129 | n/d | 1.52 | 32.5 | 12.1 | 8.42 | 0.182 | 4.38 | 5.97 | 2.93 | 3.89 | 11.4 | 1.36 |
| 25 | 63.1 | 0.099 | n/d | 1.21 | 48.3 | 19.5 | 5.53 | 0.190 | 5.51 | 4.01 | 2.29 | 2.66 | 21.8 | 1.35 |
| 30 | **270** | 0.181 | n/d | 2.43 | **67.2** | **32.0** | 14.0 | 0.329 | 8.94 | 6.31 | 7.50 | 2.77 | **75.0** | **3.69** |
| 35 | 133 | **0.201** | n/d | **3.73** | 47.2 | 28.3 | 16.9 | 0.341 | 7.70 | 8.44 | **7.95** | 2.29 | 40.6 | 3.10 |
| 40 | 37.0 | 0.062 | n/d | 1.14 | 18.6 | 13.0 | 8.12 | 0.132 | 3.93 | 5.55 | 2.89 | 1.18 | 10.8 | 1.17 |
| 45 | 43.2 | 0.062 | n/d | 1.51 | 24.4 | 15.9 | 8.34 | 0.163 | 3.88 | 4.27 | 3.39 | 1.59 | 15.1 | 1.51 |
| 50 | 20.6 | 0.023 | n/d | 0.861 | 17.2 | 8.72 | 5.88 | 0.206 | 2.82 | 7.99 | 1.90 | 1.01 | 11.9 | 0.785 |
| 55 | 28.2 | 0.034 | n/d | 0.937 | 25.2 | 10.6 | 5.93 | 0.106 | 2.62 | 1.90 | 2.42 | 1.18 | 15.4 | 0.893 |
| 60 | 7.05 | 0.013 | n/d | 0.398 | 5.82 | 4.31 | 2.71 | 0.040 | 1.02 | 0.895 | 0.741 | 0.600 | 6.04 | 0.363 |
| 65 | 5.34 | 0.017 | n/d | 0.404 | 4.56 | 3.91 | 2.35 | 0.030 | 0.906 | 2.86 | 0.945 | 0.574 | 5.48 | 0.256 |
| 70 | 5.75 | 0.057 | n/d | 0.834 | 8.40 | 6.25 | 7.86 | 0.103 | 1.92 | 1.65 | 1.37 | 0.516 | 7.85 | 0.366 |
| 75 | 8.38 | 0.070 | n/d | 0.685 | 11.3 | 8.77 | 7.83 | 0.130 | 2.80 | 1.33 | 1.22 | 0.530 | 10.1 | 0.618 |
| 80 | 7.10 | 0.106 | n/d | 0.818 | 9.17 | 8.47 | 9.27 | 0.148 | 2.66 | 39.3 | 1.48 | 0.190 | 10.9 | 0.487 |
| 85 | 7.92 | 0.113 | n/d | 0.772 | 10.5 | 8.65 | 8.72 | 0.135 | 2.71 | 0.852 | 1.11 | 0.386 | 11.5 | 0.515 |
| 90 | 5.17 | 0.067 | n/d | 0.671 | 7.73 | 6.60 | 9.85 | 0.105 | 1.76 | 1.74 | 0.791 | 0.458 | 11.6 | 0.381 |
| 95 | 4.79 | 0.046 | n/d | 0.447 | 4.96 | 4.69 | 6.09 | 0.072 | 1.10 | 0.741 | 0.404 | 0.379 | 9.29 | 0.269 |
| 100 | 3.73 | 0.041 | n/d | 0.805 | 4.25 | 3.68 | 4.58 | 0.068 | 1.03 | 3.84 | 0.731 | 0.538 | 6.98 | 0.143 |
| 105 | 10.8 | 0.107 | n/d | 1.08 | 14.9 | 11.0 | 12.9 | 0.156 | 2.56 | 1.72 | 1.07 | 1.07 | 17.5 | 0.504 |
| 110 | 15.6 | 0.053 | n/d | 0.454 | 10.9 | 5.96 | 7.24 | 0.113 | 1.54 | 1.41 | 0.590 | 0.431 | 14.5 | 0.354 |
| 115 | 13.0 | 0.113 | n/d | 0.719 | 18.0 | 9.47 | 9.69 | 0.162 | 2.94 | 1.28 | 0.891 | 0.466 | 19.1 | 0.605 |
| 120 | 9.89 | 0.052 | n/d | 0.512 | 14.5 | 6.55 | 8.21 | 0.111 | 1.47 | 1.34 | 0.494 | 0.544 | 18.4 | 0.442 |
| 125 | 9.10 | 0.052 | n/d | 0.609 | 7.49 | 5.05 | 7.72 | 0.121 | 1.33 | 0.815 | 0.415 | 0.384 | 13.7 | 0.263 |
| 130 | 6.97 | 0.035 | n/d | 0.423 | 10.0 | 4.99 | 7.22 | 0.091 | 1.17 | 1.20 | 0.260 | 0.724 | 8.26 | 0.335 |
| 135 | 28.0 | n/d | n/d | 0.409 | 47.3 | 20.6 | 16.9 | 0.709 | 4.92 | **241** | 0.787 | **7.21** | 16.6 | 1.90 |
| 140 | 48.7 | 0.063 | n/d | 0.319 | 55.7 | 19.3 | 17.6 | 0.602 | 4.57 | 1.71 | 0.857 | 3.35 | 22.4 | 1.53 |
| 145 | 19.4 | 0.028 | n/d | 0.108 | 25.7 | 11.1 | **37.2** | 0.998 | 3.23 | 1.91 | 0.669 | 2.68 | 18.1 | 1.02 |
| 150 | 11.7 | 0.018 | n/d | 0.148 | 13.0 | 7.34 | 30.2 | **7.40** | 11.5 | 7.31 | 0.626 | 2.38 | 16.9 | 1.05 |
| 155 | 6.92 | 0.001 | n/d | 0.199 | 5.95 | 3.38 | 30.0 | 3.27 | **12.1** | 2.35 | 0.192 | 2.58 | 7.58 | 0.432 |
| 160 | n/a | n/a | n/a | n/a | n/a | n/a | n/a | n/a | n/a | n/a | n/a | n/a | n/a | n/a |
| 165 | n/a | n/a | n/a | n/a | n/a | n/a | n/a | n/a | n/a | n/a | n/a | n/a | n/a | n/a |
| 170 | n/a | n/a | n/a | n/a | n/a | n/a | n/a | n/a | n/a | n/a | n/a | n/a | n/a | n/a |
| 175 | n/a | n/a | n/a | n/a | n/a | n/a | n/a | n/a | n/a | n/a | n/a | n/a | n/a | n/a |
| 180 | n/a | n/a | n/a | n/a | n/a | n/a | n/a | n/a | n/a | n/a | n/a | n/a | n/a | n/a |
| 185 | n/a | n/a | n/a | n/a | n/a | n/a | n/a | n/a | n/a | n/a | n/a | n/a | n/a | n/a |
| 190 | n/a | n/a | n/a | n/a | n/a | n/a | n/a | n/a | n/a | n/a | n/a | n/a | n/a | n/a |
| 195 | n/a | n/a | n/a | n/a | n/a | n/a | n/a | n/a | n/a | n/a | n/a | n/a | n/a | n/a |
| 200 | n/a | n/a | n/a | n/a | n/a | n/a | n/a | n/a | n/a | n/a | n/a | n/a | n/a | n/a |
| min | 0.394 | n/d | n/d | 0.108 | 1.55 | 2.53 | 2.35 | 0.030 | 0.360 | 0.741 | 0.192 | 0.190 | 0.958 | 0.015 |
| max | 270 | 0.201 | n/d | 3.73 | 67.2 | 32.0 | 37.2 | 7.40 | 12.1 | 241 | 7.95 | 7.21 | 75.0 | 3.69 |
| median | 10.8 | 0.055 | n/d | 0.772 | 13.0 | 8.65 | 8.34 | 0.135 | 2.71 | 2.35 | 1.07 | 0.820 | 11.6 | 0.515 |
| mean | 29.7 | 0.067 | n/d | 0.945 | 19.8 | 10.3 | 11.2 | 0.534 | 3.53 | 14.8 | 1.92 | 1.52 | 15.2 | 0.868 |
| geomean | 13.6 | 0.0476 | --- | 0.715 | 13.7 | 8.42 | 9.25 | 0.182 | 2.60 | 3.61 | 1.24 | 1.03 | 11.8 | 0.577 |

N/a = end of sediment core. N/d = Not detected. For statistical purposes half of the limit of detection was used for n/d samples. Bold indicate maximum concentration values.
