# Supplementary material for: Assessing the ecological risk of heavy metal sediment contamination from Port Everglades Florida USA
Source: PeerJ. 2023 Nov 14;11:e16152. doi: 10.7717/peerj.16152 (PMC10655720; doi:10.7717/peerj.16152)
Supplement: Supplemental Information 12 — N/a = end of sediment core. N/d = Not detected. For statistical purposes half of the limit of detection was used for n/d samples. Bold indicate maximum concentration values. [file peerj-11-16152-s012.docx]

| **Table S11**. Park Headquarters 2 heavy metal concentrations (µg/g) by sediment core depth (cm), minimum (min), maximum (max), median, arithmetic mean (mean), and geometric mean (geomean). | | | | | | | | | | | | | | |
| --- | --- | --- | --- | --- | --- | --- | --- | --- | --- | --- | --- | --- | --- | --- |
| cm | Mo | Cd | Hg | Pb | V | Cr | Mn | Co | Ni | Zn | Cu | Sn | As | Se |
| 5 | 0.081 | 0.013 | n/d | 1.87 | 2.12 | 2.27 | 13.1 | 0.082 | 0.656 | 8.22 | 3.71 | 0.960 | 0.933 | 0.049 |
| 10 | 0.170 | 0.006 | n/d | 1.93 | 2.09 | 1.93 | 10.5 | 0.059 | 0.529 | 7.48 | 4.38 | 1.05 | 1.05 | 0.014 |
| 15 | n/d | 0.015 | n/d | 1.61 | 1.85 | 2.75 | 9.67 | 0.054 | 0.500 | 7.59 | 3.46 | 1.37 | 0.769 | n/d |
| 20 | 33.4 | 0.159 | n/d | **4.21** | 34.7 | 21.5 | 18.8 | 0.370 | 8.77 | 9.27 | **5.28** | 1.96 | 14.4 | 2.07 |
| 25 | 65.9 | 0.490 | n/d | 2.97 | 44.2 | 20.4 | 21.7 | 0.516 | 11.8 | 7.02 | 5.11 | 1.67 | 24.4 | 2.09 |
| 30 | 54.1 | 0.555 | n/d | 2.19 | 31.3 | 12.2 | 17.4 | 0.366 | 5.41 | 6.85 | 4.01 | 1.25 | 17.8 | 1.27 |
| 35 | 96.3 | **0.697** | n/d | 2.69 | **70.8** | **42.8** | 14.9 | 0.589 | **18.6** | 3.14 | 3.01 | 1.99 | 26.3 | **2.63** |
| 40 | 35.2 | 0.168 | n/d | 0.793 | 15.7 | 9.07 | 5.54 | 0.136 | 3.44 | 0.605 | 1.68 | 0.783 | 11.1 | 0.863 |
| 45 | **119** | 0.075 | n/d | 0.960 | 30.7 | 13.7 | 7.61 | 0.220 | 4.00 | 4.44 | 2.54 | 1.73 | **32.5** | 1.60 |
| 50 | 25.0 | 0.151 | n/d | 0.694 | 16.2 | 9.93 | 6.18 | 0.153 | 3.86 | 1.48 | 1.92 | 0.694 | 11.4 | 0.882 |
| 55 | 21.5 | 0.173 | n/d | 0.630 | 22.9 | 9.84 | 6.43 | 0.208 | 4.42 | 1.33 | 1.88 | 0.729 | 11.0 | 0.831 |
| 60 | 15.4 | 0.065 | n/d | 0.539 | 15.7 | 6.92 | 3.78 | 0.094 | 2.23 | 0.866 | 1.47 | 0.572 | 9.53 | 0.631 |
| 65 | 14.5 | 0.135 | n/d | 0.745 | 19.5 | 10.4 | 6.15 | 0.151 | 4.08 | 3.59 | 1.95 | 0.658 | 10.6 | 0.842 |
| 70 | 5.50 | 0.011 | n/d | 0.350 | 5.60 | 3.67 | 1.61 | 0.024 | 0.716 | 0.537 | 0.560 | 0.869 | 5.60 | 0.317 |
| 75 | 6.92 | 0.003 | n/d | 0.512 | 3.88 | 4.39 | 2.26 | 0.029 | 0.734 | 0.710 | 0.633 | 0.674 | 5.58 | 0.323 |
| 80 | 13.1 | 0.019 | n/d | 1.08 | 8.77 | 10.7 | 5.42 | 0.075 | 1.44 | 1.20 | 1.53 | 1.02 | 14.2 | 0.809 |
| 85 | 10.6 | 0.030 | n/d | 1.03 | 8.58 | 10.5 | 3.86 | 0.108 | 1.59 | 2.97 | 1.96 | 0.814 | 12.7 | 0.659 |
| 90 | 16.9 | 0.038 | n/d | 1.37 | 15.6 | 17.9 | 9.57 | 0.144 | 2.95 | 5.69 | 2.97 | 0.616 | 20.0 | 0.933 |
| 95 | 8.98 | 0.010 | n/d | 0.815 | 6.27 | 8.59 | 3.38 | 0.059 | 1.11 | 2.49 | 1.67 | 0.267 | 13.5 | 0.555 |
| 100 | 6.95 | 0.004 | n/d | 0.652 | 5.07 | 6.68 | 2.28 | 0.044 | 0.804 | 0.278 | 1.09 | 0.215 | 11.6 | 0.373 |
| 105 | 7.27 | 0.007 | n/d | 0.596 | 6.25 | 6.66 | 2.54 | 0.045 | 0.698 | 0.935 | 0.707 | 0.567 | 11.1 | 0.351 |
| 110 | 13.2 | 0.188 | n/d | 0.990 | 10.9 | 10.2 | 22.8 | 0.293 | 4.80 | 2.15 | 1.14 | 0.867 | 19.5 | 0.509 |
| 115 | 9.38 | 0.070 | n/d | 0.734 | 7.17 | 7.60 | 11.7 | 0.113 | 1.96 | 2.05 | 0.745 | 0.874 | 13.9 | 0.366 |
| 120 | 37.5 | 0.039 | n/d | 1.05 | 18.6 | 11.4 | 4.94 | 0.087 | 1.87 | 0.816 | 1.46 | 0.442 | 30.1 | 0.808 |
| 125 | 25.1 | 0.054 | n/d | 0.810 | 25.6 | 11.4 | 5.14 | 0.102 | 1.95 | 2.27 | 1.29 | 0.344 | 30.4 | 0.887 |
| 130 | 27.0 | 0.075 | n/d | 0.889 | 15.2 | 12.4 | 8.25 | 0.146 | 2.33 | 1.31 | 1.17 | 0.594 | 22.6 | 0.613 |
| 135 | 21.3 | 0.068 | n/d | 1.17 | 10.4 | 9.58 | 7.77 | 0.131 | 1.85 | 0.919 | 0.698 | 0.722 | 23.9 | 0.431 |
| 140 | 40.3 | 0.030 | n/d | 0.335 | 52.0 | 21.7 | 34.1 | 0.833 | 4.55 | 0.399 | 0.962 | 2.62 | 20.1 | 1.61 |
| 145 | 33.3 | 0.048 | n/d | 0.451 | 38.9 | 19.4 | 21.8 | 0.920 | 6.20 | **16.2** | 1.17 | 3.40 | 20.6 | 1.86 |
| 150 | 7.35 | 0.041 | n/d | 0.357 | 10.2 | 6.18 | 7.00 | 0.396 | 2.65 | 2.69 | 0.667 | 1.39 | 8.27 | 0.678 |
| 155 | 23.5 | 0.037 | n/d | 0.245 | 24.9 | 14.0 | 14.0 | 1.84 | 6.22 | 4.09 | 1.02 | **4.71** | 15.6 | 2.00 |
| 160 | 20.2 | 0.061 | n/d | 0.290 | 15.8 | 23.8 | 27.3 | **2.26** | 8.23 | 1.36 | 1.65 | 3.23 | 19.5 | 1.85 |
| 165 | 21.2 | 0.017 | n/d | 0.077 | 12.8 | 9.79 | 16.7 | 1.40 | 10.5 | 0.320 | 1.24 | 4.59 | 12.8 | 1.68 |
| 170 | 3.06 | 0.003 | n/d | 0.032 | 2.38 | 2.22 | **43.5** | 0.321 | 3.29 | 0.313 | 0.310 | 1.28 | 5.01 | 0.516 |
| 175 | 1.78 | n/d | n/d | 0.036 | 1.61 | 1.45 | 33.1 | 0.219 | 2.57 | 0.161 | 0.101 | 0.853 | 5.22 | 0.375 |
| 180 | 1.63 | 0.001 | n/d | 0.055 | 1.95 | 1.68 | 34.0 | 0.322 | 3.65 | 0.147 | 0.313 | 1.01 | 3.96 | 0.273 |
| 185 | 2.98 | 0.008 | n/d | 0.255 | 2.88 | 2.66 | 28.5 | 0.447 | 7.72 | 0.320 | 0.781 | 2.13 | 4.99 | 0.333 |
| 190 | 4.52 | 0.001 | n/d | 0.848 | 4.58 | 4.10 | 14.3 | 0.237 | 9.56 | 3.33 | 0.975 | 1.71 | 4.14 | 0.565 |
| 195 | n/a | n/a | n/a | n/a | n/a | n/a | n/a | n/a | n/a | n/a | n/a | n/a | n/a | n/a |
| 200 | n/a | n/a | n/a | n/a | n/a | n/a | n/a | n/a | n/a | n/a | n/a | n/a | n/a | n/a |
| min | n/d | n/d | n/d | 0.032 | 1.61 | 1.45 | 1.61 | 0.024 | 0.500 | 0.147 | 0.101 | 0.215 | 0.769 | n/d |
| max | 119 | 0.697 | n/d | 4.21 | 70.8 | 42.8 | 43.5 | 2.26 | 18.6 | 16.2 | 5.28 | 4.71 | 32.5 | 2.63 |
| median | 15.4 | 0.039 | n/d | 0.769 | 11.9 | 9.81 | 9.62 | 0.152 | 3.12 | 1.77 | 1.37 | 0.917 | 12.7 | 0.678 |
| mean | 11.62 | 0.0330 | nd | 0.620 | 10.4 | 7.97 | 9.61 | 0.189 | 2.81 | 1.63 | 1.33 | 1.04 | 10.4 | 0.644 |
| geomean | 11.62 | 0.0330 | --- | 0.620 | 10.4 | 7.97 | 9.61 | 0.189 | 2.81 | 1.63 | 1.33 | 1.04 | 10.4 | 0.644 |

N/a = end of sediment core. N/d = Not detected. For statistical purposes half of the limit of detection was used for n/d samples. Bold indicate maximum concentration values.
