# Supplementary material for: Assessing the ecological risk of heavy metal sediment contamination from Port Everglades Florida USA
Source: PeerJ. 2023 Nov 14;11:e16152. doi: 10.7717/peerj.16152 (PMC10655720; doi:10.7717/peerj.16152)
Supplement: Supplemental Information 13 — N/a = end of sediment core. N/d = Not detected. For statistical purposes half of the limit of detection was used for n/d samples. Bold indicate maximum concentration values. [file peerj-11-16152-s013.docx]

| **Table S12**. South Turning Basin 1 heavy metal concentrations (µg/g) by sediment core depth (cm), minimum (min), maximum (max), median, arithmetic mean (mean), and geometric mean (geomean). | | | | | | | | | | | | | | | |
| --- | --- | --- | --- | --- | --- | --- | --- | --- | --- | --- | --- | --- | --- | --- | --- |
| cm | Mo | Cd | Hg | Pb | V | Cr | Mn | Co | Ni | Zn | Cu | Sn | As | Se |  |
| 5 | 8.25 | 0.173 | n/d | 19.2 | 25.8 | 15.0 | 28.8 | 0.547 | 9.43 | 134 | 110 | 2.73 | 21.5 | 1.23 |  |
| 10 | **88.5** | 0.250 | n/d | 8.06 | 45.7 | 15.8 | 23.3 | 0.846 | 7.74 | 55.7 | 42.2 | 3.77 | **59.9** | 1.93 |  |
| 15 | 17.2 | 0.330 | **0.058** | **27.1** | 40.1 | **19.8** | 45.4 | 0.890 | 15.4 | 151 | 127 | 3.01 | 21.5 | 1.69 |  |
| 20 | 14.9 | **0.335** | 0.025 | 27.0 | 37.7 | 19.2 | 57.4 | 0.909 | 15.4 | 180 | **129** | 4.03 | 20.9 | 1.41 |  |
| 25 | 16.4 | 0.297 | n/d | 21.7 | **51.7** | 17.6 | 62.3 | 1.05 | **15.9** | **190** | 104 | 4.16 | 22.8 | 1.82 |  |
| 30 | 31.9 | 0.285 | 0.056 | 16.5 | 43.3 | 13.4 | 59.9 | 0.980 | 12.6 | 91.1 | 67.2 | **5.04** | 37.7 | 1.86 |  |
| 35 | 25.4 | 0.145 | n/d | 9.47 | 29.0 | 11.2 | 45.4 | 1.06 | 8.53 | 61.3 | 48.4 | 3.15 | 31.7 | 1.65 |  |
| 40 | 26.0 | 0.164 | n/d | 9.57 | 31.1 | 17.2 | 38.7 | 0.784 | 10.2 | 49.9 | 39.3 | 3.00 | 21.3 | 1.61 |  |
| 45 | 25.0 | 0.071 | n/d | 5.48 | 28.2 | 17.6 | 29.6 | 0.986 | 10.4 | 23.3 | 18.4 | 3.89 | 16.6 | 1.81 |  |
| 50 | 44.8 | 0.126 | n/d | 1.71 | 32.6 | 14.1 | 18.7 | **1.50** | 8.29 | 5.95 | 2.36 | 4.73 | 18.7 | **2.05** |  |
| 55 | 29.7 | 0.065 | n/d | 1.79 | 33.8 | 9.05 | 11.3 | 1.43 | 6.31 | 5.81 | 5.70 | 4.02 | 15.7 | 1.50 |  |
| 60 | 17.7 | 0.166 | n/d | 9.11 | 29.6 | 12.5 | 63.9 | 0.995 | 8.32 | 45.4 | 45.3 | 2.26 | 21.3 | 1.40 |  |
| 65 | 4.85 | 0.044 | n/d | 1.17 | 7.40 | 4.49 | 63.3 | 0.475 | 1.90 | 9.60 | 4.10 | 1.00 | 14.6 | 0.800 |  |
| 70 | 0.074 | 0.004 | n/d | 1.01 | 2.83 | 2.98 | **102** | 0.212 | 1.30 | 0.627 | 0.690 | 0.180 | 3.01 | 0.377 |  |
| 75 | 0.453 | 0.013 | n/d | 1.70 | 7.71 | 4.73 | 89.5 | 0.512 | 2.22 | 2.06 | 1.14 | 0.082 | 4.78 | 0.407 |  |
| 80 | n/a | n/a | n/a | n/a | n/a | n/a | n/a | n/a | n/a | n/a | n/a | n/a | n/a | n/a |  |
| 85 | n/a | n/a | n/a | n/a | n/a | n/a | n/a | n/a | n/a | n/a | n/a | n/a | n/a | n/a |  |
| 90 | n/a | n/a | n/a | n/a | n/a | n/a | n/a | n/a | n/a | n/a | n/a | n/a | n/a | n/a |  |
| 95 | n/a | n/a | n/a | n/a | n/a | n/a | n/a | n/a | n/a | n/a | n/a | n/a | n/a | n/a |  |
| 100 | n/a | n/a | n/a | n/a | n/a | n/a | n/a | n/a | n/a | n/a | n/a | n/a | n/a | n/a |  |
| 105 | n/a | n/a | n/a | n/a | n/a | n/a | n/a | n/a | n/a | n/a | n/a | n/a | n/a | n/a |  |
| 110 | n/a | n/a | n/a | n/a | n/a | n/a | n/a | n/a | n/a | n/a | n/a | n/a | n/a | n/a |  |
| 115 | n/a | n/a | n/a | n/a | n/a | n/a | n/a | n/a | n/a | n/a | n/a | n/a | n/a | n/a |  |
| 120 | n/a | n/a | n/a | n/a | n/a | n/a | n/a | n/a | n/a | n/a | n/a | n/a | n/a | n/a |  |
| 125 | n/a | n/a | n/a | n/a | n/a | n/a | n/a | n/a | n/a | n/a | n/a | n/a | n/a | n/a |  |
| 130 | n/a | n/a | n/a | n/a | n/a | n/a | n/a | n/a | n/a | n/a | n/a | n/a | n/a | n/a |  |
| 135 | n/a | n/a | n/a | n/a | n/a | n/a | n/a | n/a | n/a | n/a | n/a | n/a | n/a | n/a |  |
| 140 | n/a | n/a | n/a | n/a | n/a | n/a | n/a | n/a | n/a | n/a | n/a | n/a | n/a | n/a |  |
| 145 | n/a | n/a | n/a | n/a | n/a | n/a | n/a | n/a | n/a | n/a | n/a | n/a | n/a | n/a |  |
| 150 | n/a | n/a | n/a | n/a | n/a | n/a | n/a | n/a | n/a | n/a | n/a | n/a | n/a | n/a |  |
| 155 | n/a | n/a | n/a | n/a | n/a | n/a | n/a | n/a | n/a | n/a | n/a | n/a | n/a | n/a |  |
| 160 | n/a | n/a | n/a | n/a | n/a | n/a | n/a | n/a | n/a | n/a | n/a | n/a | n/a | n/a |  |
| 165 | n/a | n/a | n/a | n/a | n/a | n/a | n/a | n/a | n/a | n/a | n/a | n/a | n/a | n/a |  |
| 170 | n/a | n/a | n/a | n/a | n/a | n/a | n/a | n/a | n/a | n/a | n/a | n/a | n/a | n/a |  |
| 175 | n/a | n/a | n/a | n/a | n/a | n/a | n/a | n/a | n/a | n/a | n/a | n/a | n/a | n/a |  |
| 180 | n/a | n/a | n/a | n/a | n/a | n/a | n/a | n/a | n/a | n/a | n/a | n/a | n/a | n/a |  |
| 185 | n/a | n/a | n/a | n/a | n/a | n/a | n/a | n/a | n/a | n/a | n/a | n/a | n/a | n/a |  |
| 190 | n/a | n/a | n/a | n/a | n/a | n/a | n/a | n/a | n/a | n/a | n/a | n/a | n/a | n/a |  |
| 195 | n/a | n/a | n/a | n/a | n/a | n/a | n/a | n/a | n/a | n/a | n/a | n/a | n/a | n/a |  |
| 200 | n/a | n/a | n/a | n/a | n/a | n/a | n/a | n/a | n/a | n/a | n/a | n/a | n/a | n/a |  |
| min | 0.074 | 0.004 | n/d | 1.01 | 2.83 | 2.98 | 11.3 | 0.212 | 1.30 | 0.627 | 0.690 | 0.082 | 3.01 | 0.377 |  |
| max | 88.5 | 0.335 | 0.058 | 27.1 | 51.7 | 19.8 | 102 | 1.50 | 15.9 | 190 | 129 | 5.04 | 59.9 | 2.05 |  |
| median | 17.7 | 0.164 | 0.056 | 9.11 | 31.1 | 14.1 | 45.4 | 0.909 | 8.53 | 49.9 | 42.2 | 3.15 | 21.3 | 1.61 |  |
| mean | 23.4 | 0.164 | 0.047 | 10.7 | 29.8 | 13.0 | 49.3 | 0.879 | 8.93 | 67.0 | 49.6 | 3.00 | 22.1 | 1.44 |  |
| geomean | 11.3 | 0.105 | 0.0433 | 6.36 | 24.1 | 11.4 | 42.5 | 0.799 | 7.22 | 27.9 | 20.1 | 2.08 | 18.1 | 1.30 |  |

N/a = end of sediment core. N/d = Not detected. For statistical purposes half of the limit of detection was used for n/d samples. Bold indicate maximum concentration values.
