# Supplementary material for: Assessing the ecological risk of heavy metal sediment contamination from Port Everglades Florida USA
Source: PeerJ. 2023 Nov 14;11:e16152. doi: 10.7717/peerj.16152 (PMC10655720; doi:10.7717/peerj.16152)
Supplement: Supplemental Information 14 — N/a = end of sediment core. N/d = Not detected. For statistical purposes half of the limit of detection was used for n/d samples. Bold indicate maximum concentration values. [file peerj-11-16152-s014.docx]

| **Table S13**. South Turning Basin 2 heavy metal concentrations (µg/g) by sediment core depth (cm), minimum (min), maximum (max), median, arithmetic mean (mean), and geometric mean (geomean). | | | | | | | | | | | | | | | | | | |
| --- | --- | --- | --- | --- | --- | --- | --- | --- | --- | --- | --- | --- | --- | --- | --- | --- | --- | --- |
| cm | Mo | Cd | | Hg | Pb | V | Cr | Mn | Co | Ni | Zn | Cu | Sn | | As | Se |  |  |
| 5 | 8.67 | 0.264 | | 0.116 | 22.6 | 30.5 | 17.2 | 38.8 | 0.667 | 12.7 | 133 | 115 | 2.80 | | 15.5 | 1.33 |  |  |
| 10 | 9.75 | 0.336 | | **0.189** | 27.4 | 37.5 | 20.2 | **49.3** | 0.756 | 15.9 | 156 | 135 | 3.71 | | 15.1 | 1.61 |  |  |
| 15 | 9.49 | **0.350** | | 0.160 | **28.3** | **39.4** | **20.3** | 44.7 | **0.781** | **16.4** | **157** | **137** | 2.95 | | 13.9 | **1.67** |  |  |
| 20 | 14.5 | 0.083 | n/d | | 5.07 | 16.7 | 5.68 | 17.7 | 0.518 | 5.11 | 22.3 | 35.0 | 8.14 | **15.6** | | 0.300 | |  |
| 25 | 8.97 | 0.040 | | n/d | 0.639 | 6.85 | 5.82 | 4.07 | 0.457 | 2.49 | 11.6 | 1.29 | 4.52 | | 9.11 | 0.461 |  |  |
| 30 | 3.05 | 0.051 | | n/d | 5.09 | 6.81 | 3.45 | 8.55 | 0.160 | 2.59 | 27.7 | 22.8 | 1.03 | | 4.73 | 0.249 |  |  |
| 35 | 9.84 | 0.041 | | n/d | 0.480 | 8.40 | 4.57 | 3.39 | 0.619 | 2.00 | 5.00 | 1.20 | 8.60 | | 12.6 | 0.500 |  |  |
| 40 | 8.57 | n/d | | n/d | 0.687 | 5.06 | 2.99 | 8.33 | 0.621 | 1.76 | 8.32 | 2.63 | 7.19 | | 14.5 | 0.421 |  |  |
| 45 | 14.6 | 0.045 | | n/d | 0.832 | 7.49 | 2.64 | 4.13 | 0.712 | 1.33 | 23.7 | 1.67 | 10.3 | | 14.5 | 0.630 |  |  |
| 50 | **14.8** | 0.062 | | n/d | 0.264 | 6.76 | 2.80 | 5.31 | 0.663 | 1.46 | 4.47 | 0.359 | **11.1** | | 13.3 | 0.408 |  |  |
| 55 | n/a | n/a | | n/a | n/a | n/a | n/a | n/a | n/a | n/a | n/a | n/a | n/a | | n/a | n/a |  |  |
| 60 | n/a | n/a | | n/a | n/a | n/a | n/a | n/a | n/a | n/a | n/a | n/a | n/a | | n/a | n/a |  |  |
| 65 | n/a | n/a | | n/a | n/a | n/a | n/a | n/a | n/a | n/a | n/a | n/a | n/a | | n/a | n/a |  |  |
| 70 | n/a | n/a | | n/a | n/a | n/a | n/a | n/a | n/a | n/a | n/a | n/a | n/a | | n/a | n/a |  |  |
| 75 | n/a | n/a | | n/a | n/a | n/a | n/a | n/a | n/a | n/a | n/a | n/a | n/a | | n/a | n/a |  |  |
| 80 | n/a | n/a | | n/a | n/a | n/a | n/a | n/a | n/a | n/a | n/a | n/a | n/a | | n/a | n/a |  |  |
| 85 | n/a | n/a | | n/a | n/a | n/a | n/a | n/a | n/a | n/a | n/a | n/a | n/a | | n/a | n/a |  |  |
| 90 | n/a | n/a | | n/a | n/a | n/a | n/a | n/a | n/a | n/a | n/a | n/a | n/a | | n/a | n/a |  |  |
| 95 | n/a | n/a | | n/a | n/a | n/a | n/a | n/a | n/a | n/a | n/a | n/a | n/a | | n/a | n/a |  |  |
| 100 | n/a | n/a | | n/a | n/a | n/a | n/a | n/a | n/a | n/a | n/a | n/a | n/a | | n/a | n/a |  |  |
| 105 | n/a | n/a | | n/a | n/a | n/a | n/a | n/a | n/a | n/a | n/a | n/a | n/a | | n/a | n/a |  |  |
| 110 | n/a | n/a | | n/a | n/a | n/a | n/a | n/a | n/a | n/a | n/a | n/a | n/a | | n/a | n/a |  |  |
| 115 | n/a | n/a | | n/a | n/a | n/a | n/a | n/a | n/a | n/a | n/a | n/a | n/a | | n/a | n/a |  |  |
| 120 | n/a | n/a | | n/a | n/a | n/a | n/a | n/a | n/a | n/a | n/a | n/a | n/a | | n/a | n/a |  |  |
| 125 | n/a | n/a | | n/a | n/a | n/a | n/a | n/a | n/a | n/a | n/a | n/a | n/a | | n/a | n/a |  |  |
| 130 | n/a | n/a | | n/a | n/a | n/a | n/a | n/a | n/a | n/a | n/a | n/a | n/a | | n/a | n/a |  |  |
| 135 | n/a | n/a | | n/a | n/a | n/a | n/a | n/a | n/a | n/a | n/a | n/a | n/a | | n/a | n/a |  |  |
| 140 | n/a | n/a | | n/a | n/a | n/a | n/a | n/a | n/a | n/a | n/a | n/a | n/a | | n/a | n/a |  |  |
| 145 | n/a | n/a | | n/a | n/a | n/a | n/a | n/a | n/a | n/a | n/a | n/a | n/a | | n/a | n/a |  |  |
| 150 | n/a | n/a | | n/a | n/a | n/a | n/a | n/a | n/a | n/a | n/a | n/a | n/a | | n/a | n/a |  |  |
| 155 | n/a | n/a | | n/a | n/a | n/a | n/a | n/a | n/a | n/a | n/a | n/a | n/a | | n/a | n/a |  |  |
| 160 | n/a | n/a | | n/a | n/a | n/a | n/a | n/a | n/a | n/a | n/a | n/a | n/a | | n/a | n/a |  |  |
| 165 | n/a | n/a | | n/a | n/a | n/a | n/a | n/a | n/a | n/a | n/a | n/a | n/a | | n/a | n/a |  |  |
| 170 | n/a | n/a | | n/a | n/a | n/a | n/a | n/a | n/a | n/a | n/a | n/a | n/a | | n/a | n/a |  |  |
| 175 | n/a | n/a | | n/a | n/a | n/a | n/a | n/a | n/a | n/a | n/a | n/a | n/a | | n/a | n/a |  |  |
| 180 | n/a | n/a | | n/a | n/a | n/a | n/a | n/a | n/a | n/a | n/a | n/a | n/a | | n/a | n/a |  |  |
| 185 | n/a | n/a | | n/a | n/a | n/a | n/a | n/a | n/a | n/a | n/a | n/a | n/a | | n/a | n/a |  |  |
| 190 | n/a | n/a | | n/a | n/a | n/a | n/a | n/a | n/a | n/a | n/a | n/a | n/a | | n/a | n/a |  |  |
| 195 | n/a | n/a | | n/a | n/a | n/a | n/a | n/a | n/a | n/a | n/a | n/a | n/a | | n/a | n/a |  |  |
| 200 | n/a | n/a | | n/a | n/a | n/a | n/a | n/a | n/a | n/a | n/a | n/a | n/a | | n/a | n/a |  |  |
| min | 3.05 | n/d | | n/d | 0.264 | 5.06 | 2.64 | 3.39 | 0.160 | 1.33 | 4.47 | 0.359 | 1.03 | | 4.73 | 0.249 |  |  |
| max | 14.8 | 0.350 | | 0.189 | 28.3 | 39.4 | 20.3 | 49.3 | 0.781 | 16.4 | 157 | 137 | 11.1 | | 15.6 | 1.67 |  |  |
| median | 9.62 | 0.062 | | 0.160 | 2.95 | 7.94 | 5.13 | 8.44 | 0.642 | 2.54 | 23.0 | 12.7 | 5.85 | | 14.2 | 0.480 |  |  |
| mean | 10.2 | 0.141 | | 0.155 | 9.15 | 16.5 | 8.57 | 18.4 | 0.595 | 6.17 | 54.8 | 45.2 | 6.03 | | 12.9 | 0.758 |  |  |
| geomean | 9.47 | 0.0945 | | 0.152 | 2.70 | 12.2 | 6.18 | 11.3 | 0.554 | 3.87 | 25.2 | 9.16 | 4.91 | | 12.3 | 0.606 |  |  |

N/a = end of sediment core. N/d = Not detected. For statistical purposes half of the limit of detection was used for n/d samples. Bold indicate maximum concentration values.
