# Supplementary material for: Assessing the ecological risk of heavy metal sediment contamination from Port Everglades Florida USA
Source: PeerJ. 2023 Nov 14;11:e16152. doi: 10.7717/peerj.16152 (PMC10655720; doi:10.7717/peerj.16152)
Supplement: Supplemental Information 15 — N/a = end of sediment core. N/d = Not detected. For statistical purposes half of the limit of detection was used for n/d samples. Bold indicate maximum concentration values. [file peerj-11-16152-s015.docx]

| **Table S14**. West Lake 1 heavy metal concentrations (µg/g) by sediment core depth (cm), minimum (min), maximum (max), median, arithmetic mean (mean), and geometric mean (geomean). | | | | | | | | | | | | | | |
| --- | --- | --- | --- | --- | --- | --- | --- | --- | --- | --- | --- | --- | --- | --- |
| cm | Mo | Cd | Hg | Pb | V | Cr | Mn | Co | Ni | Zn | Cu | Sn | As | Se |
| 5 | n/d | 0.040 | 0.013 | 5.77 | 3.44 | 5.35 | 28.4 | 0.119 | 1.49 | **49.8** | **30.4** | 0.580 | 8.12 | 0.386 |
| 10 | 0.048 | 0.065 | n/d | 7.52 | 4.14 | 5.47 | 28.4 | 0.142 | 1.72 | 47.2 | 25.3 | 0.466 | 9.04 | 0.366 |
| 15 | 0.581 | 0.089 | 0.044 | 8.47 | 4.98 | 5.82 | 27.8 | 0.165 | 1.84 | 38.6 | 17.9 | 0.687 | 8.38 | 0.321 |
| 20 | 0.833 | 0.134 | 0.088 | 11.0 | **9.20** | **8.75** | 35.9 | 0.349 | 2.87 | 19.2 | 10.1 | **1.21** | 6.22 | 0.425 |
| 25 | 0.222 | 0.088 | n/d | 6.53 | 4.30 | 6.39 | 24.0 | 0.211 | 1.60 | 11.5 | 5.55 | 0.502 | 5.28 | 0.253 |
| 30 | 0.244 | 0.050 | n/d | 3.28 | 4.76 | 6.01 | 18.1 | 0.281 | 1.52 | 4.07 | 1.95 | 0.586 | 4.63 | 0.250 |
| 35 | 0.336 | 0.087 | 0.056 | 6.40 | 5.37 | 7.33 | 26.0 | 0.261 | 1.82 | 19.4 | 10.3 | 0.837 | 4.21 | 0.291 |
| 40 | 0.228 | 0.072 | n/d | 4.70 | 5.17 | 7.46 | 43.5 | 0.343 | 1.78 | 5.82 | 3.05 | 0.454 | 2.96 | 0.264 |
| 45 | 0.576 | **0.282** | 0.197 | **16.4** | 8.30 | 8.37 | 55.9 | **0.367** | **3.07** | 30.5 | 15.6 | 0.904 | 10.4 | 0.445 |
| 50 | 0.789 | 0.113 | 0.145 | 11.9 | 7.43 | 6.66 | 72.6 | 0.349 | 2.76 | 23.3 | 11.7 | 1.18 | 12.5 | 0.525 |
| 55 | 0.511 | 0.097 | **0.212** | 10.0 | 6.47 | 5.18 | 63.2 | 0.300 | 2.28 | 20.4 | 9.26 | 0.488 | 10.8 | 0.572 |
| 60 | 0.856 | 0.045 | 0.003 | 7.10 | 5.32 | 4.13 | 70.6 | 0.289 | 1.55 | 12.8 | 4.10 | 0.827 | 10.3 | 0.567 |
| 65 | 0.339 | 0.023 | n/d | 4.87 | 4.04 | 3.30 | 55.4 | 0.212 | 1.35 | 7.80 | 3.15 | 0.378 | 11.7 | 0.487 |
| 70 | 0.583 | 0.044 | n/d | 5.00 | 4.86 | 3.39 | 72.3 | 0.247 | 1.31 | 10.9 | 2.96 | 0.285 | **14.0** | **0.713** |
| 75 | **1.39** | 0.038 | n/d | 3.36 | 4.09 | 2.96 | 70.1 | 0.279 | 1.23 | 6.60 | 1.63 | 0.585 | 12.1 | 0.593 |
| 80 | 0.270 | 0.010 | n/d | 0.642 | 1.21 | 0.973 | **80.1** | 0.183 | 0.438 | 1.07 | 0.430 | 0.312 | 4.85 | 0.179 |
| 85 | n/d | 0.004 | n/d | 1.07 | 1.34 | 2.80 | 79.0 | 0.223 | 0.649 | 0.916 | 0.287 | 0.190 | 2.60 | 0.115 |
| 90 | n/d | 0.008 | n/d | 1.64 | 2.04 | 3.39 | 71.0 | 0.251 | 0.716 | 4.94 | 1.53 | 0.302 | 3.18 | 0.178 |
| 95 | n/a | n/a | n/a | n/a | n/a | n/a | n/a | n/a | n/a | n/a | n/a | n/a | n/a | n/a |
| 100 | n/a | n/a | n/a | n/a | n/a | n/a | n/a | n/a | n/a | n/a | n/a | n/a | n/a | n/a |
| 105 | n/a | n/a | n/a | n/a | n/a | n/a | n/a | n/a | n/a | n/a | n/a | n/a | n/a | n/a |
| 110 | n/a | n/a | n/a | n/a | n/a | n/a | n/a | n/a | n/a | n/a | n/a | n/a | n/a | n/a |
| 115 | n/a | n/a | n/a | n/a | n/a | n/a | n/a | n/a | n/a | n/a | n/a | n/a | n/a | n/a |
| 120 | n/a | n/a | n/a | n/a | n/a | n/a | n/a | n/a | n/a | n/a | n/a | n/a | n/a | n/a |
| 125 | n/a | n/a | n/a | n/a | n/a | n/a | n/a | n/a | n/a | n/a | n/a | n/a | n/a | n/a |
| 130 | n/a | n/a | n/a | n/a | n/a | n/a | n/a | n/a | n/a | n/a | n/a | n/a | n/a | n/a |
| 135 | n/a | n/a | n/a | n/a | n/a | n/a | n/a | n/a | n/a | n/a | n/a | n/a | n/a | n/a |
| 140 | n/a | n/a | n/a | n/a | n/a | n/a | n/a | n/a | n/a | n/a | n/a | n/a | n/a | n/a |
| 145 | n/a | n/a | n/a | n/a | n/a | n/a | n/a | n/a | n/a | n/a | n/a | n/a | n/a | n/a |
| 150 | n/a | n/a | n/a | n/a | n/a | n/a | n/a | n/a | n/a | n/a | n/a | n/a | n/a | n/a |
| 155 | n/a | n/a | n/a | n/a | n/a | n/a | n/a | n/a | n/a | n/a | n/a | n/a | n/a | n/a |
| 160 | n/a | n/a | n/a | n/a | n/a | n/a | n/a | n/a | n/a | n/a | n/a | n/a | n/a | n/a |
| 165 | n/a | n/a | n/a | n/a | n/a | n/a | n/a | n/a | n/a | n/a | n/a | n/a | n/a | n/a |
| 170 | n/a | n/a | n/a | n/a | n/a | n/a | n/a | n/a | n/a | n/a | n/a | n/a | n/a | n/a |
| 175 | n/a | n/a | n/a | n/a | n/a | n/a | n/a | n/a | n/a | n/a | n/a | n/a | n/a | n/a |
| 180 | n/a | n/a | n/a | n/a | n/a | n/a | n/a | n/a | n/a | n/a | n/a | n/a | n/a | n/a |
| 185 | n/a | n/a | n/a | n/a | n/a | n/a | n/a | n/a | n/a | n/a | n/a | n/a | n/a | n/a |
| 190 | n/a | n/a | n/a | n/a | n/a | n/a | n/a | n/a | n/a | n/a | n/a | n/a | n/a | n/a |
| 195 | n/a | n/a | n/a | n/a | n/a | n/a | n/a | n/a | n/a | n/a | n/a | n/a | n/a | n/a |
| 200 | n/a | n/a | n/a | n/a | n/a | n/a | n/a | n/a | n/a | n/a | n/a | n/a | n/a | n/a |
| min | n/d | 0.004 | n/d | 0.642 | 1.21 | 0.973 | 18.1 | 0.119 | 0.438 | 0.916 | 0.287 | 0.190 | 2.60 | 0.115 |
| max | 1.39 | 0.282 | 0.212 | 16.4 | 9.20 | 8.75 | 80.1 | 0.367 | 3.07 | 49.8 | 30.4 | 1.21 | 14.0 | 0.713 |
| median | 0.511 | 0.057 | 0.072 | 6.08 | 4.81 | 5.41 | 55.6 | 0.256 | 1.58 | 12.2 | 4.82 | 0.541 | 8.25 | 0.376 |
| mean | 0.521 | 0.072 | 0.095 | 6.43 | 4.80 | 5.21 | 51.2 | 0.254 | 1.66 | 17.5 | 8.62 | 0.599 | 7.85 | 0.385 |
| geomean | 0.408 | 0.0474 | 0.0517 | 4.96 | 4.26 | 4.68 | 46.2 | 0.243 | 1.50 | 10.9 | 4.63 | 0.533 | 6.92 | 0.347 |

N/a = end of sediment core. N/d = Not detected. For statistical purposes half of the limit of detection was used for n/d samples. Bold indicate maximum concentration values.
