# Supplementary material for: Assessing the ecological risk of heavy metal sediment contamination from Port Everglades Florida USA
Source: PeerJ. 2023 Nov 14;11:e16152. doi: 10.7717/peerj.16152 (PMC10655720; doi:10.7717/peerj.16152)
Supplement: Supplemental Information 16 — N/a = end of sediment core. N/d = Not detected. For statistical purposes half of the limit of detection was used for n/d samples. Bold indicate maximum concentration values. [file peerj-11-16152-s016.docx]

| Table S15. West Lake 2 heavy metal concentrations (µg/g) by sediment core depth (cm), minimum (min), maximum (max), median, arithmetic mean (mean), and geometric mean (geomean). | | | | | | | | | | | | | | |
| --- | --- | --- | --- | --- | --- | --- | --- | --- | --- | --- | --- | --- | --- | --- |
| cm | Mo | Cd | Hg | Pb | V | Cr | Mn | Co | Ni | Zn | Cu | Sn | As | Se |
| 5 | 0.307 | 0.100 | n/d | 11.4 | 4.94 | 6.80 | 34.6 | 0.173 | 1.98 | **37.7** | **21.1** | 0.502 | 6.42 | 0.267 |
| 10 | 0.074 | 0.070 | n/d | 7.24 | 4.08 | 6.35 | 26.8 | 0.178 | 1.63 | 28.3 | 14.0 | 0.606 | 5.23 | 0.288 |
| 15 | n/d | 0.032 | n/d | 2.04 | 2.36 | 5.17 | 15.2 | 0.169 | 1.08 | 8.48 | 1.03 | 0.344 | 1.68 | 0.170 |
| 20 | 0.195 | 0.045 | n/d | 4.23 | 4.36 | 6.24 | 17.8 | 0.266 | 1.65 | 2.44 | 6.70 | 0.314 | 3.54 | 0.263 |
| 25 | 0.155 | 0.074 | 0.006 | 4.54 | 5.45 | 7.29 | 22.6 | 0.294 | 1.85 | 7.15 | 4.02 | 0.676 | 2.99 | 0.251 |
| 30 | 0.207 | 0.072 | n/d | 5.01 | 5.14 | 7.53 | 54.8 | 0.386 | 2.01 | 8.96 | 4.81 | 0.064 | 3.25 | 0.255 |
| 35 | 0.090 | 0.179 | 0.060 | 7.83 | 5.92 | 4.57 | 46.3 | 0.208 | 2.24 | 16.6 | 8.95 | 0.624 | 11.0 | 0.563 |
| 40 | 0.458 | **0.209** | **0.262** | **13.1** | 6.50 | 6.67 | 70.1 | 0.334 | 2.64 | 21.1 | 11.7 | 0.478 | 12.3 | 0.604 |
| 45 | 0.551 | 0.091 | 0.125 | 10.2 | 5.58 | 5.64 | 75.3 | 0.318 | 2.21 | 16.0 | 9.12 | 0.528 | 11.2 | 0.647 |
| 50 | 0.648 | 0.119 | 0.112 | 11.6 | 7.85 | 7.21 | 78.7 | 0.414 | 2.88 | 21.2 | 11.4 | 0.493 | 12.6 | 0.546 |
| 55 | 0.414 | 0.055 | 0.044 | 8.96 | 5.12 | 4.56 | 66.1 | 0.295 | 1.92 | 16.8 | 7.89 | 0.496 | 10.6 | 0.528 |
| 60 | 0.726 | 0.037 | n/d | 6.51 | 5.78 | 4.29 | 77.2 | 0.309 | 1.61 | 8.65 | 2.99 | 0.452 | 10.5 | 0.507 |
| 65 | 0.956 | 0.053 | n/d | 5.68 | 5.36 | 4.10 | 66.6 | 0.311 | 1.59 | 9.12 | 3.52 | 0.387 | 13.8 | 0.433 |
| 70 | 0.975 | 0.055 | n/d | 4.54 | 6.15 | 4.13 | 66.5 | 0.293 | 1.65 | 10.9 | 2.43 | 0.583 | 11.0 | 0.561 |
| 75 | **3.61** | 0.016 | n/d | 0.586 | **59.6** | **11.2** | 9.38 | **0.520** | **16.8** | 27.7 | 1.20 | **7.70** | 12.9 | **0.885** |
| 80 | 2.04 | 0.012 | n/d | 0.965 | 2.24 | 1.94 | 57.1 | 0.277 | 0.975 | 2.45 | 0.513 | 0.675 | **21.7** | 0.704 |
| 85 | 0.813 | 0.033 | n/d | 2.19 | 2.78 | 2.53 | 70.8 | 0.216 | 1.17 | 7.41 | 0.927 | 0.957 | 8.75 | 0.460 |
| 90 | 0.038 | n/d | n/d | 0.145 | 0.432 | 0.337 | **93.8** | 0.190 | 0.597 | 0.409 | 0.260 | 0.601 | 4.62 | 0.215 |
| 95 | n/a | n/a | n/a | n/a | n/a | n/a | n/a | n/a | n/a | n/a | n/a | n/a | n/a | n/a |
| 100 | n/a | n/a | n/a | n/a | n/a | n/a | n/a | n/a | n/a | n/a | n/a | n/a | n/a | n/a |
| 105 | n/a | n/a | n/a | n/a | n/a | n/a | n/a | n/a | n/a | n/a | n/a | n/a | n/a | n/a |
| 110 | n/a | n/a | n/a | n/a | n/a | n/a | n/a | n/a | n/a | n/a | n/a | n/a | n/a | n/a |
| 115 | n/a | n/a | n/a | n/a | n/a | n/a | n/a | n/a | n/a | n/a | n/a | n/a | n/a | n/a |
| 120 | n/a | n/a | n/a | n/a | n/a | n/a | n/a | n/a | n/a | n/a | n/a | n/a | n/a | n/a |
| 125 | n/a | n/a | n/a | n/a | n/a | n/a | n/a | n/a | n/a | n/a | n/a | n/a | n/a | n/a |
| 130 | n/a | n/a | n/a | n/a | n/a | n/a | n/a | n/a | n/a | n/a | n/a | n/a | n/a | n/a |
| 135 | n/a | n/a | n/a | n/a | n/a | n/a | n/a | n/a | n/a | n/a | n/a | n/a | n/a | n/a |
| 140 | n/a | n/a | n/a | n/a | n/a | n/a | n/a | n/a | n/a | n/a | n/a | n/a | n/a | n/a |
| 145 | n/a | n/a | n/a | n/a | n/a | n/a | n/a | n/a | n/a | n/a | n/a | n/a | n/a | n/a |
| 150 | n/a | n/a | n/a | n/a | n/a | n/a | n/a | n/a | n/a | n/a | n/a | n/a | n/a | n/a |
| 155 | n/a | n/a | n/a | n/a | n/a | n/a | n/a | n/a | n/a | n/a | n/a | n/a | n/a | n/a |
| 160 | n/a | n/a | n/a | n/a | n/a | n/a | n/a | n/a | n/a | n/a | n/a | n/a | n/a | n/a |
| 165 | n/a | n/a | n/a | n/a | n/a | n/a | n/a | n/a | n/a | n/a | n/a | n/a | n/a | n/a |
| 170 | n/a | n/a | n/a | n/a | n/a | n/a | n/a | n/a | n/a | n/a | n/a | n/a | n/a | n/a |
| 175 | n/a | n/a | n/a | n/a | n/a | n/a | n/a | n/a | n/a | n/a | n/a | n/a | n/a | n/a |
| 180 | n/a | n/a | n/a | n/a | n/a | n/a | n/a | n/a | n/a | n/a | n/a | n/a | n/a | n/a |
| 185 | n/a | n/a | n/a | n/a | n/a | n/a | n/a | n/a | n/a | n/a | n/a | n/a | n/a | n/a |
| 190 | n/a | n/a | n/a | n/a | n/a | n/a | n/a | n/a | n/a | n/a | n/a | n/a | n/a | n/a |
| 195 | n/a | n/a | n/a | n/a | n/a | n/a | n/a | n/a | n/a | n/a | n/a | n/a | n/a | n/a |
| 200 | n/a | n/a | n/a | n/a | n/a | n/a | n/a | n/a | n/a | n/a | n/a | n/a | n/a | n/a |
| min | n/d | n/d | n/d | 0.145 | 0.432 | 0.337 | 9.38 | 0.169 | 0.597 | 0.409 | 0.260 | 0.064 | 1.68 | 0.170 |
| max | 3.61 | 0.209 | 0.262 | 13.1 | 59.6 | 11.2 | 93.8 | 0.520 | 16.8 | 37.7 | 21.1 | 7.70 | 21.7 | 0.885 |
| median | 0.458 | 0.055 | 0.086 | 5.35 | 5.25 | 5.41 | 61.6 | 0.293 | 1.75 | 10.0 | 4.42 | 0.515 | 10.6 | 0.484 |
| mean | 0.721 | 0.074 | 0.102 | 5.93 | 7.76 | 5.36 | 52.8 | 0.286 | 2.58 | 14.0 | 6.25 | 0.915 | 9.12 | 0.453 |
| geomean | 0.394 | 0.058 | 0.062 | 3.94 | 4.75 | 4.50 | 44.6 | 0.273 | 1.87 | 9.62 | 3.64 | 0.543 | 7.60 | 0.410 |

N/a = end of sediment core. N/d = Not detected. For statistical purposes half of the limit of detection was used for n/d samples. Bold indicate maximum concentration values.
