# Supplementary material for: Assessing the ecological risk of heavy metal sediment contamination from Port Everglades Florida USA
Source: PeerJ. 2023 Nov 14;11:e16152. doi: 10.7717/peerj.16152 (PMC10655720; doi:10.7717/peerj.16152)
Supplement: Supplemental Information 17 — N/d = Not detected. For statistical purposes half of the limit of detection was used for n/d samples. [file peerj-11-16152-s017.docx]

| Table S16. North Reef (NR) heavy metal concentrations (µg/g) of surface sediment samples (5cm) with minimum (min), maximum (max), median, arithmetic mean (mean), and geometric mean (geomean). | | | | | | | | | | | | | | |
| --- | --- | --- | --- | --- | --- | --- | --- | --- | --- | --- | --- | --- | --- | --- |
|  | Mo | Cd | Hg | Pb | V | Cr | Mn | Co | Ni | Zn | Cu | Sn | As | Se |
| NR 1 | n/d | 0.0100 | n/d | 0.900 | 5.02 | 4.70 | 10.1 | 0.0400 | 0.410 | 2.76 | 0.670 | 0.990 | 6.89 | 0.110 |
| NR 2 | n/d | 0.0300 | n/d | 1.40 | 6.80 | 6.81 | 13.9 | 0.100 | 0.72 | 4.52 | 1.02 | 1.84 | 6.37 | 0.0400 |
| NR 3 | 0.01 | 0.0400 | n/d | 1.50 | 9.12 | 7.55 | 15.6 | 0.0400 | 0.600 | 3.24 | 0.65 | 1.60 | 8.55 | 0.0800 |
| min | 0.00005 | 0.0100 | 0.00005 | 0.90 | 5.02 | 4.70 | 10.1 | 0.0400 | 0.410 | 2.76 | 0.650 | 0.990 | 6.37 | 0.0400 |
| max | 0.010 | 0.0400 | 0.00005 | 1.50 | 9.12 | 7.55 | 15.6 | 0.1 | 0.72 | 4.52 | 1.02 | 1.84 | 8.55 | 0.110 |
| median | 0.00005 | 0.0300 | 0.00005 | 1.40 | 6.80 | 6.81 | 13.9 | 0.04 | 0.6 | 3.24 | 0.67 | 1.60 | 6.89 | 0.080 |
| mean | 0.00337 | 0.0267 | 0.00005 | 1.27 | 6.98 | 6.35 | 13.2 | 0.06 | 0.58 | 3.51 | 0.78 | 1.48 | 7.27 | 0.077 |
| geomean | 0.000292 | 0.0229 | 0.00005 | 1.24 | 6.78 | 6.23 | 13.0 | 0.0543 | 0.562 | 3.43 | 0.763 | 1.43 | 7.21 | 0.0706 |

N/d = Not detected. For statistical purposes half of the limit of detection was used for n/d samples.
