# Supplementary material for: Assessing the ecological risk of heavy metal sediment contamination from Port Everglades Florida USA
Source: PeerJ. 2023 Nov 14;11:e16152. doi: 10.7717/peerj.16152 (PMC10655720; doi:10.7717/peerj.16152)
Supplement: Supplemental Information 18 — N/d = Not detected. For statistical purposes half of the limit of detection was used for n/d samples. [file peerj-11-16152-s018.docx]

| Table S17. South Reef (SR) heavy metal concentrations (µg/g) of surface sediment samples (5cm) with minimum (min), maximum (max), median, arithmetic mean (mean), and geometric mean (geomean). | | | | | | | | | | | | | | |
| --- | --- | --- | --- | --- | --- | --- | --- | --- | --- | --- | --- | --- | --- | --- |
|  | Mo | Cd | Hg | Pb | V | Cr | Mn | Co | Ni | Zn | Cu | Sn | As | Se |
| SR 1 | 0.00900 | 0.0390 | 0.00500 | 1.25 | 3.80 | 5.32 | 24.10 | 0.0310 | 0.616 | 91.1 | 28.6 | 1.85 | 2.41 | 0.0710 |
| SR 2 | 0.00005 | 0.0300 | 0.00001 | 1.50 | 4.19 | 6.35 | 13.60 | 0.0300 | 0.750 | 3.85 | 0.59 | 2.07 | 3.82 | 0.120 |
| SR 3 | 0.00005 | 0.0400 | 0.00001 | 1.80 | 3.91 | 6.44 | 14.60 | 0.0500 | 0.860 | 6.97 | 0.510 | 1.34 | 3.32 | 0.0800 |
| min | 0.00005 | 0.0300 | 0.00001 | 1.25 | 3.80 | 5.32 | 13.6 | 0.0300 | 0.616 | 3.85 | 0.510 | 1.34 | 2.41 | 0.0710 |
| max | 0.00900 | 0.0400 | 0.00500 | 1.80 | 4.19 | 6.44 | 24.1 | 0.0500 | 0.860 | 91.1 | 28.6 | 2.07 | 3.82 | 0.120 |
| median | 0.000050 | 0.0390 | 0.000005 | 1.50 | 3.91 | 6.35 | 14.6 | 0.0310 | 0.750 | 6.97 | 0.590 | 1.85 | 3.32 | 0.0800 |
| mean | 0.00303 | 0.0363 | 0.00167 | 1.52 | 3.97 | 6.04 | 17.4 | 0.0370 | 0.742 | 34.0 | 9.90 | 1.75 | 3.18 | 0.0903 |
| geomean | 0.000282 | 0.0360 | 0.00005 | 1.50 | 3.96 | 6.01 | 16.9 | 0.0360 | 0.735 | 13.5 | 2.05 | 1.72 | 3.13 | 0.0880 |

N/d = Not detected. For statistical purposes half of the limit of detection was used for n/d samples.
