# Supplementary material for: Assessing the ecological risk of heavy metal sediment contamination from Port Everglades Florida USA
Source: PeerJ. 2023 Nov 14;11:e16152. doi: 10.7717/peerj.16152 (PMC10655720; doi:10.7717/peerj.16152)
Supplement: Supplemental Information 19 [file peerj-11-16152-s019.docx]

**Table S18**. Metal concentration significant variations across depths for all cores per location. X denotes significant Kruskal-Wallace test results. No Wilcoxon pairwise with Holm correction follow-ups were significant.

|  |  | | | | | |
| --- | --- | --- | --- | --- | --- | --- |
|  | **Dania Cutoff Canal** | **Park Education Center** | **Park Headquarters** | **South Turning Basin** | **West Lake** |  |
| **Mo** | X | X | X |  |  |  |
| **Cd** | X |  |  |  |  |  |
| **Hg** |  |  |  |  |  |  |
| **Pb** | X |  | X |  |  |  |
| **V** | X |  | X |  |  |  |
| **Cr** | X |  |  |  |  |  |
| **Mn** | X |  |  |  |  |  |
| **Co** |  |  | X |  | X |  |
| **Ni** | X |  | X |  |  |  |
| **Zn** | X |  |  |  |  |  |
| **Cu** |  |  | X |  | X |  |
| **Sn** |  |  | X |  |  |  |
| **As** | X |  | X |  |  |  |
| **Se** |  |  | X |  |  |  |
