# Supplementary material for: Assessing the ecological risk of heavy metal sediment contamination from Port Everglades Florida USA
Source: PeerJ. 2023 Nov 14;11:e16152. doi: 10.7717/peerj.16152 (PMC10655720; doi:10.7717/peerj.16152)
Supplement: Supplemental Information 20 — Bolded numbers indicate some degree of contamination. Avg = average; StErr = standard error; CI LB = confidence interval lower bound; 4 - 5: strongly to extremely contaminated; 3 - 4: strongly contaminated; 2 - 3: moderately to strongly contaminated; 1 - 2: moderately contaminated; 0 - 1: uncontaminated to moderately contaminated; < 0: uncontaminated. [file peerj-11-16152-s020.docx]

**Table S19**. Geo-accumulation indices and statistical analyses for Mo and As of all the cores per location and depth.

| **Dania Cut-off Canal (DCC)** | | | | | | | | | | | | |
| --- | --- | --- | --- | --- | --- | --- | --- | --- | --- | --- | --- | --- |
| cm | Core 1 Mo | Core 2 Mo | Core 3 Mo | Avg | StErr | CI LB | Core 1 As | Core 2 As | Core 3 As | Avg | StErr | CI LB |
| 5 | **2.84** | -2.24 | 1.88 | **0.83** | 1.56 | -3.72 | 4.02 | -1.49 | 3.01 | 1.85 | 1.69 | **-3.09** |
| 10 | **0.40** | **4.35** | **2.39** | **2.38** | 1.14 | -0.95 | 2.35 | 4.30 | 3.23 | 3.29 | 0.56 | **1.64** |
| 15 | **3.16** | **4.29** | **5.93** | **4.46** | 0.80 | **2.11** | 4.25 | 4.20 | 4.54 | 4.33 | 0.10 | **4.02** |
| 20 | **4.01** | **4.33** | **3.02** | **3.79** | 0.39 | **2.63** | 4.88 | 4.64 | 2.23 | 3.92 | 0.85 | **1.45** |
| 25 | **4.01** | **4.69** | **4.11** | **4.27** | 0.21 | **3.65** | 5.02 | 4.37 | 3.29 | 4.23 | 0.50 | **2.75** |
| 30 | **3.47** | **4.81** | **5.84** | **4.71** | 0.69 | **2.70** | 4.56 | 5.01 | 4.51 | 4.69 | 0.16 | **4.23** |
| 35 | **3.06** | **4.54** | **6.77** | **4.79** | 1.08 | **1.64** | 4.01 | 4.55 | 5.66 | 4.74 | 0.49 | **3.32** |
| 40 | **4.35** | **4.76** | **3.54** | **4.21** | 0.36 | **3.17** | 4.77 | 5.16 | 4.59 | 4.84 | 0.17 | **4.35** |
| 45 | **2.99** | **3.57** | **7.42** | **4.66** | 1.39 | **0.60** | 3.57 | 4.95 | 6.63 | 5.05 | 0.89 | **2.47** |
| 50 | **1.65** | **5.30** | **1.86** | **2.94** | 1.18 | -0.52 | 2.48 | 5.81 | 2.99 | 3.76 | 1.03 | **0.74** |
| 55 | **0.73** | **1.90** | **1.33** | **1.32** | 0.34 | **0.33** | 2.59 | 2.96 | 2.85 | 2.80 | 0.11 | **2.48** |
| 60 | **1.04** | **1.29** | -1.82 | **0.17** | 1.00 | -2.74 | 2.63 | 2.51 | 1.49 | 2.21 | 0.36 | **1.16** |
| 65 | -0.23 | **1.63** | -2.71 | -0.44 | 1.26 | -4.11 | 2.45 | 2.86 | 1.85 | 2.39 | 0.29 | **1.53** |
| 70 | -1.36 | **3.11** | -0.58 | **0.39** | 1.38 | -3.63 | 2.99 | 4.61 | 1.95 | 3.19 | 0.77 | **0.92** |
| 75 | **0.38** | -1.98 | -2.22 | -1.27 | 0.83 | -3.70 | 2.73 | 1.79 | 2.64 | 2.39 | 0.30 | **1.51** |
| 80 |  | -0.71 | -3.36 | -2.04 | 1.32 | -10.39 |  | 2.20 | 2.56 | 2.38 | 0.18 | **1.25** |
| 85 |  | -1.53 | -3.20 | -2.37 | 0.83 | -7.62 |  | 2.66 | 2.17 | 2.42 | 0.25 | **0.85** |
| 90 |  | -0.50 | -3.26 | -1.88 | 1.38 | -10.59 |  | 2.62 | 2.45 | 2.53 | 0.08 | **2.00** |
| 95 |  |  | -0.10 |  |  |  |  |  | 2.48 |  |  |  |
| 100 |  |  | **3.72** |  |  |  |  |  | 3.51 |  |  |  |
|  | | | | | | | | | | | | |
| **Park Education Center (PEC)** | | | | | | | | | | | | |
| cm | Core 1 Mo | Core 2 Mo | Core 3 Mo | Avg | StErr | CI LB | Core 1 As | Core 2 As | Core 3 As | Avg | StErr | CI LB |
| 5 | -5.11 | **1.14** | **2.17** | -0.60 | 2.27 | -7.24 | -0.75 | 3.07 | **3.30** | **1.87** | 1.31 | -1.96 |
| 10 | -4.17 | -3.26 | -0.59 | -2.67 | 1.08 | -5.82 | -1.81 | -0.52 | **0.74** | -0.53 | 0.73 | -2.68 |
| 15 | -1.60 | -4.31 | -3.11 | -3.01 | 0.78 | -5.29 | -0.16 | -1.89 | -0.87 | -0.98 | 0.50 | -2.44 |
| 20 | -3.45 | -4.43 | -4.05 | -3.98 | 0.29 | -4.81 | -1.54 | -1.58 | -1.17 | -1.43 | 0.13 | -1.81 |
| 25 | -1.11 | -1.06 | -4.20 | -2.13 | 1.04 | -5.16 | 0.32 | **0.22** | -1.12 | -0.19 | 0.46 | -1.55 |
| 30 | -0.93 | -0.71 | -0.82 | -0.82 | 0.06 | -1.00 | 0.81 | **0.51** | **0.66** | **0.66** | 0.08 | **0.41** |
| 35 | -0.91 | -0.71 | -3.54 | -1.72 | 0.91 | -4.38 | 0.39 | **0.12** | -1.20 | -0.23 | 0.49 | -1.66 |
| 40 | -0.57 | -1.71 | -0.11 | -0.80 | 0.47 | -2.18 | 1.01 | -0.04 | **0.87** | **0.61** | 0.33 | -0.35 |
| 45 | -2.34 | -0.58 | -1.94 | -1.62 | 0.53 | -3.17 | 0.40 | **1.04** | 0.00 | **0.48** | 0.30 | -0.41 |
| 50 | -1.22 | -0.13 | -1.13 | -0.83 | 0.35 | -1.85 | 0.97 | **0.90** | **0.75** | **0.88** | 0.07 | **0.68** |
| 55 | **0.48** | -0.42 | -0.80 | -0.25 | 0.38 | -1.35 | 2.42 | **0.61** | **0.15** | **1.06** | 0.69 | -0.97 |
| 60 | **0.78** | -1.11 | -0.83 | -0.38 | 0.59 | -2.10 | 2.43 | **0.67** | **0.75** | **1.28** | 0.57 | -0.39 |
| 65 | **0.34** | -1.20 | -0.30 | -0.39 | 0.45 | -1.70 | 1.45 | **0.55** | **0.94** | **0.98** | 0.26 | **0.22** |
| 70 | **0.31** | 190 | -0.24 | **0.20** | 0.23 | -0.48 | 1.32 | **1.99** | **0.82** | **1.38** | 0.34 | **0.38** |
| 75 | -1.35 | 195 | -0.16 | -0.07 | 0.76 | -2.30 | -0.10 | **2.21** | **0.46** | **0.85** | 0.70 | -1.18 |
| 80 | -1.48 | 200 | -0.34 | -0.04 | 0.93 | -2.75 | -0.99 | **1.98** | **0.56** | **0.52** | 0.86 | -1.99 |
| 85 | -0.19 | 190 | **0.11** | **0.26** | 0.31 | -0.65 | 0.52 | **1.84** | **0.83** | **1.07** | 0.40 | -0.10 |
| 90 | -2.22 | 195 | **0.74** | **0.16** | 1.24 | -3.46 | -1.41 | **1.87** | **1.54** | **0.67** | 1.04 | -2.37 |
| 95 | **1.19** | -2.04 | **0.91** | **0.02** | 1.03 | -3.00 | 1.36 | -0.04 | **1.55** | **0.96** | 0.50 | -0.51 |
| 100 | **2.03** | -2.14 | **1.00** | **0.30** | 1.25 | -3.36 | 2.30 | -1.05 | **1.89** | **1.04** | 1.05 | -2.03 |
| 105 | -0.77 | -1.91 | **1.67** | -0.34 | 1.06 | -3.43 | 0.32 | -1.48 | **2.68** | **0.50** | 1.20 | -3.01 |
| 110 | **0.81** | **0.89** | **1.56** | **1.09** | 0.24 | **0.39** | 1.64 | **1.18** | **1.67** | **1.49** | 0.16 | **1.04** |
| 115 | **0.96** | **1.49** | -1.58 | **0.29** | 0.95 | -2.48 | 2.36 | **2.36** | -0.59 | **1.38** | 0.98 | -1.49 |
| 120 | **1.86** | **1.56** | **0.91** | **1.44** | 0.28 | **0.62** | 2.78 | **1.85** | **1.39** | **2.00** | 0.41 | **0.81** |
| 125 | **1.99** | **1.88** | **1.89** | **1.92** | 0.03 | **1.82** | 2.59 | **2.24** | **2.41** | **2.41** | 0.10 | **2.12** |
| 130 | **4.28** | **5.32** | -1.50 | **2.70** | 2.12 | -3.50 | 3.83 | **4.79** | -0.90 | **2.58** | 1.76 | -2.56 |
| 135 | **2.65** | **3.75** | -0.14 | **2.08** | 1.16 | -1.29 | 2.81 | **3.29** | **0.57** | **2.22** | 0.84 | -0.22 |
| 140 | **0.37** | **1.64** | **2.18** | **1.39** | 0.54 | -0.17 | 1.70 | **1.88** | **2.28** | **1.95** | 0.17 | **1.45** |
| 145 | **0.27** | **0.48** | **3.74** | **1.50** | 1.12 | -1.78 | 0.76 | **1.58** | **3.63** | **1.99** | 0.85 | -0.50 |
| 150 | -1.51 | 1.95 | **0.08** | **0.17** | 1.00 | -2.75 | -0.59 | **2.44** | **1.23** | **1.03** | 0.88 | -1.54 |
| 155 | -1.54 | -2.24 | -2.30 | -2.03 | 0.24 | -2.74 | -1.35 | -0.85 | -0.67 | -0.96 | 0.20 | -1.56 |
| 160 | **0.02** | -3.22 | **2.79** | -0.13 | 1.74 | -5.20 | 0.83 | -1.49 | **3.07** | **0.80** | 1.32 | -3.04 |
| 165 | -0.59 | -1.55 | **2.61** | **0.16** | 1.26 | -3.52 | 0.35 | -0.63 | **2.77** | **0.83** | 1.01 | -2.12 |
| 170 | -0.89 | **0.24** | **1.80** | **0.38** | 0.78 | -1.90 | -0.14 | **0.58** | **2.22** | **0.89** | 0.70 | -1.15 |
| 175 | -2.40 | -0.37 | **1.60** | -0.39 | 1.16 | -3.76 | -1.02 | **0.78** | **1.72** | **0.49** | 0.80 | -1.85 |
| 180 | -2.74 |  | **1.85** | -0.45 | 2.29 | -14.93 | -1.26 |  | **2.24** | **0.49** | 1.75 | -10.58 |
| 185 |  |  | **1.64** |  |  |  |  |  | **2.11** |  |  |  |
| 190 |  |  | **3.30** |  |  |  |  |  | **3.81** |  |  |  |
| 195 |  |  | **1.89** |  |  |  |  |  | **1.60** |  |  |  |
| 200 |  |  | -2.40 |  |  |  |  |  | -0.98 |  |  |  |
|  | | | | | | | | | | | | |
| **Park Head Quarters (PHQ)** | | | | | | | | | | | | |
| cm | Core 1 Mo | Core 2 Mo |  | Avg | StErr | CI LB | Core 1 As | Core 2 As |  | Avg | StErr | CI LB |
| 5 | -2.51 | -4.80 |  | -3.66 | 1.14 | -10.86 | -1.23 | -1.27 |  | -1.25 | 0.02 | -1.37 |
| 10 | **0.61** | -3.73 |  | -1.56 | 2.17 | -15.23 | **0.34** | -1.10 |  | -0.38 | 0.72 | -4.95 |
| 15 | **4.17** | -11.00 |  | -3.42 | 7.58 | -51.29 | **2.33** | -1.55 |  | **0.39** | 1.94 | -11.84 |
| 20 | **4.32** | **3.89** |  | **4.11** | 0.21 | **2.75** | **2.34** | **2.68** |  | **2.51** | 0.17 | **1.44** |
| 25 | **4.81** | **4.87** |  | **4.84** | 0.03 | **4.64** | **3.27** | **3.44** |  | **3.36** | 0.08 | **2.84** |
| 30 | **6.90** | **4.59** |  | **5.75** | 1.16 | -1.57 | **5.06** | **2.98** |  | **4.02** | 1.04 | -2.54 |
| 35 | **5.89** | **5.42** |  | **5.66** | 0.24 | **4.17** | **4.17** | **3.55** |  | **3.86** | 0.31 | **1.89** |
| 40 | **4.04** | **3.97** |  | **4.00** | 0.04 | **3.78** | **2.26** | **2.30** |  | **2.28** | 0.02 | **2.14** |
| 45 | **4.26** | **5.72** |  | **4.99** | 0.73 | **0.38** | **2.75** | **3.85** |  | **3.30** | 0.55 | -0.18 |
| 50 | **3.20** | **3.47** |  | **3.33** | 0.14 | **2.46** | **2.41** | **2.35** |  | **2.38** | 0.03 | **2.19** |
| 55 | **3.65** | **3.26** |  | **3.45** | 0.19 | **2.22** | **2.77** | **2.29** |  | **2.53** | 0.24 | **1.00** |
| 60 | **1.65** | **2.77** |  | **2.21** | 0.56 | -1.35 | **1.42** | **2.08** |  | **1.75** | 0.33 | -0.32 |
| 65 | **1.25** | **2.69** |  | **1.97** | 0.72 | -2.58 | **1.29** | **2.23** |  | **1.76** | 0.47 | -1.23 |
| 70 | **1.35** | **1.29** |  | **1.32** | 0.03 | **1.12** | **1.80** | **1.32** |  | **1.56** | 0.24 | **0.02** |
| 75 | **1.90** | **1.62** |  | **1.76** | 0.14 | **0.89** | **2.17** | **1.31** |  | **1.74** | 0.43 | -0.98 |
| 80 | **1.66** | **2.54** |  | **2.10** | 0.44 | -0.69 | **2.27** | **2.66** |  | **2.46** | 0.19 | **1.24** |
| 85 | **1.82** | **2.24** |  | **2.03** | 0.21 | **0.70** | **2.35** | **2.49** |  | **2.42** | 0.07 | **1.98** |
| 90 | **1.20** | **2.91** |  | **2.05** | 0.85 | -3.34 | **2.37** | **3.15** |  | **2.76** | 0.39 | **0.30** |
| 95 | **1.09** | **2.00** |  | **1.54** | 0.45 | -1.32 | **2.05** | **2.59** |  | **2.32** | 0.27 | **0.60** |
| 100 | **0.73** | **1.63** |  | **1.18** | 0.45 | -1.65 | **1.63** | **2.37** |  | **2.00** | 0.37 | -0.32 |
| 105 | **2.26** | **1.69** |  | **1.98** | 0.28 | **0.18** | **2.96** | **2.31** |  | **2.63** | 0.33 | 0.57 |
| 110 | **2.80** | **2.55** |  | **2.67** | 0.12 | **1.90** | **2.69** | **3.12** |  | **2.90** | 0.21 | **1.55** |
| 115 | **2.53** | **2.06** |  | **2.29** | 0.23 | **0.82** | **3.09** | **2.63** |  | **2.86** | 0.23 | **1.42** |
| 120 | **2.14** | **4.06** |  | **3.10** | 0.96 | -2.97 | **3.03** | **3.74** |  | **3.38** | 0.36 | **1.13** |
| 125 | **2.02** | **3.48** |  | **2.75** | 0.73 | -1.87 | **2.61** | **3.76** |  | **3.18** | 0.58 | -0.45 |
| 130 | **1.63** | **3.58** |  | **2.61** | 0.98 | -3.56 | **1.88** | **3.33** |  | **2.60** | 0.73 | -1.98 |
| 135 | **3.64** | **3.24** |  | **3.44** | 0.20 | **2.20** | **2.88** | **3.41** |  | **3.15** | 0.26 | **1.48** |
| 140 | **4.44** | **4.16** |  | **4.30** | 0.14 | **3.44** | **3.31** | **3.16** |  | **3.24** | 0.08 | **2.75** |
| 145 | **3.11** | **3.89** |  | **3.50** | 0.39 | **1.04** | **3.01** | **3.20** |  | **3.10** | 0.09 | **2.51** |
| 150 | **2.38** | **1.71** |  | **2.04** | 0.34 | -0.08 | **2.91** | **1.88** |  | **2.39** | 0.52 | -0.87 |
| 155 | **1.62** | **3.38** |  | **2.50** | 0.88 | -3.07 | **1.75** | **2.79** |  | **2.27** | 0.52 | -1.01 |
| 160 |  | **3.17** |  |  |  |  |  | **3.12** |  |  |  |  |
| 165 |  | **3.24** |  |  |  |  |  | **2.51** |  |  |  |  |
| 170 |  | **0.44** |  |  |  |  |  | **1.16** |  |  |  |  |
| 175 |  | -0.34 |  |  |  |  |  | **1.21** |  |  |  |  |
| 180 |  | -0.47 |  |  |  |  |  | **0.81** |  |  |  |  |
| 185 |  | **0.41** |  |  |  |  |  | **1.15** |  |  |  |  |
| 190 |  | **1.01** |  |  |  |  |  | **0.88** |  |  |  |  |
|  | | | | | | | | | | | | |
| **South Turning Basin (STB)** | | | | | | | | | | | | |
| cm | Core 1 Mo | Core 2 Mo |  | Avg | StErr | CI LB | Core 1 As | Core 2 As |  | Avg | StErr | CI LB |
| 5 | **1.87** | **1.95** |  | **1.91** | 0.04 | **1.69** | **3.26** | **2.79** |  | **3.02** | 0.23 | **1.54** |
| 10 | **5.30** | **2.12** |  | **3.71** | 1.59 | -6.34 | **4.74** | **2.75** |  | **3.74** | 0.99 | -2.52 |
| 15 | **2.94** | **2.08** |  | **2.51** | 0.43 | -0.21 | **3.25** | **2.63** |  | **2.94** | 0.31 | **0.97** |
| 20 | **2.73** | **2.69** |  | **2.71** | 0.02 | **2.60** | **3.22** | **2.79** |  | **3.00** | 0.21 | **1.65** |
| 25 | **2.87** | **1.99** |  | **2.43** | 0.44 | -0.33 | **3.34** | **2.02** |  | **2.68** | 0.66 | -1.50 |
| 30 | **3.82** | **0.44** |  | **2.13** | 1.69 | -8.55 | **4.07** | **1.07** |  | **2.57** | 1.50 | -6.89 |
| 35 | **3.50** | **2.13** |  | **2.81** | 0.68 | -1.51 | **3.82** | **2.49** |  | **3.15** | 0.67 | -1.05 |
| 40 | **3.53** | **1.93** |  | **2.73** | 0.80 | -2.32 | **3.24** | **2.69** |  | **2.96** | 0.28 | **1.22** |
| 45 | **3.47** | **2.70** |  | **3.08** | 0.39 | **0.63** | **2.88** | **2.69** |  | **2.78** | 0.10 | **2.17** |
| 50 | **4.32** | **2.72** |  | **3.52** | 0.80 | -1.52 | **3.05** | **2.56** |  | **2.81** | 0.25 | **1.26** |
| 55 | **3.72** |  |  |  |  |  | **2.81** |  |  |  |  |  |
| 60 | **2.97** |  |  |  |  |  | **3.25** |  |  |  |  |  |
| 65 | **1.11** |  |  |  |  |  | **2.70** |  |  |  |  |  |
| 70 | -4.94 |  |  |  |  |  | **0.42** |  |  |  |  |  |
| 75 | -2.31 |  |  |  |  |  | **1.09** |  |  |  |  |  |
|  | | | | | | | | | | | | |
| **West Lake (WL)** | | | | | | | | | | | | |
| cm | Core 1 Mo | Core 2 Mo |  | Avg | StErr | CI LB | Core 1 As | Core 2 As |  | Avg | StErr | CI LB |
| 5 | -11.00 | -2.88 |  | -6.94 | 4.06 | -32.58 | **1.85** | **1.51** |  | **1.68** | 0.17 | **0.61** |
| 10 | -5.54 | -4.92 |  | -5.23 | 0.31 | -7.17 | **2.01** | **1.22** |  | **1.61** | 0.39 | -0.88 |
| 15 | -1.95 | -11.00 |  | -6.48 | 4.52 | -35.03 | **1.90** | **-0.42** |  | **0.74** | 1.16 | -6.58 |
| 20 | -1.43 | -3.53 |  | -2.48 | 1.05 | -9.09 | **1.47** | **0.66** |  | **1.06** | 0.41 | -1.50 |
| 25 | -3.34 | -3.86 |  | -3.60 | 0.26 | -5.23 | **1.23** | **0.41** |  | **0.82** | 0.41 | -1.77 |
| 30 | -3.20 | -3.44 |  | -3.32 | 0.12 | -4.07 | **1.04** | **0.53** |  | **0.79** | 0.25 | -0.82 |
| 35 | -2.74 | -4.64 |  | -3.69 | 0.95 | -9.68 | **0.90** | **2.29** |  | **1.59** | 0.69 | -2.77 |
| 40 | -3.31 | -2.30 |  | -2.80 | 0.51 | -5.99 | **0.40** | **2.46** |  | **1.43** | 1.03 | -5.07 |
| 45 | -1.97 | -2.03 |  | -2.00 | 0.03 | -2.20 | **2.21** | **2.32** |  | **2.27** | 0.05 | **1.93** |
| 50 | -1.51 | -1.80 |  | -1.65 | 0.14 | -2.55 | **2.47** | **2.48** |  | **2.48** | 0.00 | **2.45** |
| 55 | -2.14 | -2.44 |  | -2.29 | 0.15 | -3.25 | **2.26** | **2.23** |  | **2.25** | 0.01 | **2.16** |
| 60 | -1.39 | -1.63 |  | -1.51 | 0.12 | -2.26 | **2.19** | **2.23** |  | **2.21** | 0.02 | **2.11** |
| 65 | -2.73 | -1.23 |  | -1.98 | 0.75 | -6.70 | **2.38** | **2.62** |  | **2.50** | 0.12 | **1.74** |
| 70 | -1.95 | -1.21 |  | -1.58 | 0.37 | -3.92 | **2.64** | **2.29** |  | **2.46** | 0.17 | **1.37** |
| 75 | -0.69 | **0.68** |  | 0.00 | 0.69 | -4.34 | **2.42** | **2.52** |  | **2.47** | 0.05 | **2.17** |
| 80 | -3.06 | -0.14 |  | -1.60 | 1.46 | -10.81 | **1.11** | **3.27** |  | **2.19** | 1.08 | -4.63 |
| 85 | -11.00 | -1.47 |  | -6.23 | 4.76 | -36.32 | **0.21** | **1.96** |  | **1.09** | 0.87 | -4.44 |
| 90 | -11.00 | -5.88 |  | -8.44 | 2.56 | -24.59 | **0.50** | **1.04** |  | **0.77** | 0.27 | -0.94 |
|  | | | | | | | | | | | | |
| **North Reef (NR)** | | | | | | | | | | | | |
| cm | Core 1 Mo | Core 2 Mo | Core 3 Mo | Avg | StErr | CI LB | Core 1 As | Core 2 As | Core 3 Mo | Avg | StErr | CI LB |
| 5 | -11.00 | -11.00 | -7.81 | -9.94 | 1.06 | -13.04 | **1.61** | **1.50** | **1.93** | **1.68** | 0.13 | **1.31** |
|  | | | | | | | | | | | | |
| **South Reef (SR)** | | | | | | | | | | | | |
| cm | Core 1 Mo | Core 2 Mo | Core 3 Mo | Avg | StErr | CI LB | Core 1 As | Core 2 As | Core 3 Mo | Avg | StErr | CI LB |
| 5 | -11.00 | -11.00 | -11.00 | 11.00 | 0.00 | -11.00 | **0.28** | **0.76** | **0.56** | **0.53** | 0.14 | **0.12** |

Bolded numbers indicate some degree of contamination. Avg = average; StErr = standard error; CI LB = confidence interval lower bound; 4 - 5: strongly to extremely contaminated; 3 - 4: strongly contaminated; 2 - 3: moderately to strongly contaminated; 1 - 2: moderately contaminated; 0 - 1: uncontaminated to moderately contaminated; < 0: uncontaminated.
