# Supplementary material for: Assessing the ecological risk of heavy metal sediment contamination from Port Everglades Florida USA
Source: PeerJ. 2023 Nov 14;11:e16152. doi: 10.7717/peerj.16152 (PMC10655720; doi:10.7717/peerj.16152)
Supplement: Supplemental Information 21 — Bolded numbers (PLI < 1) indicate pollution is present. Avg = average; StErr = standard error; CI LB = confidence interval lower bound. [file peerj-11-16152-s021.docx]

**Table S20**. Pollution load indices (PLI) and statistical analyses for all cores and sediments per depth.

| **Dania Cut-off Canal (DCC)** | | | | | | |
| --- | --- | --- | --- | --- | --- | --- |
| cm | Core 1 | Core 2 | Core 3 | Avg | StErr | CI LB |
| 5 | **1.09** | 0.044 | 0.298 | 0.476 | 0.313 | -0.439 |
| 10 | 0.322 | 0.969 | 0.380 | 0.557 | 0.207 | -0.047 |
| 15 | 0.585 | 0.921 | 0.456 | 0.654 | 0.139 | 0.249 |
| 20 | 0.647 | **1.16** | 0.313 | 0.706 | 0.246 | -0.011 |
| 25 | 0.815 | **1.07** | 0.260 | 0.715 | 0.239 | 0.016 |
| 30 | 0.442 | 0.775 | 0.492 | 0.570 | 0.104 | 0.267 |
| 35 | 0.482 | 0.955 | 0.447 | 0.628 | 0.164 | 0.149 |
| 40 | 0.525 | 0.738 | 0.278 | 0.514 | 0.133 | 0.125 |
| 45 | 0.329 | 0.708 | 0.423 | 0.487 | 0.114 | 0.154 |
| 50 | 0.136 | 1.201 | 0.074 | 0.470 | 0.366 | -0.598 |
| 55 | 0.076 | 0.112 | 0.041 | 0.077 | 0.021 | 0.016 |
| 60 | 0.089 | 0.085 | 0.021 | 0.065 | 0.022 | 0.001 |
| 65 | 0.065 | 0.147 | 0.025 | 0.079 | 0.036 | -0.026 |
| 70 | 0.140 | 0.602 | 0.032 | 0.258 | 0.175 | -0.253 |
| 75 | 0.195 | 0.046 | 0.019 | 0.087 | 0.055 | -0.073 |
| 80 |  | 0.065 | 0.036 | 0.050 | 0.014 | -0.040 |
| 85 |  | 0.050 | 0.046 | 0.048 | 0.002 | 0.036 |
| 90 |  | 0.127 | 0.031 | 0.079 | 0.048 | -0.225 |
| 95 |  |  | 0.069 |  |  |  |
| 100 |  |  | 0.189 |  |  |  |
| **Park Education Center (PEC)** | | | | | | |
| cm | Core 1 | Core 2 | Core 3 | Avg | StErr | CI LB |
| 5 | 0.072 | **1.41** | **1.43** | 0.971 | 0.449 | -0.342 |
| 10 | 0.045 | 0.130 | 0.250 | 0.142 | 0.060 | -0.032 |
| 15 | 0.110 | 0.064 | 0.089 | 0.088 | 0.013 | 0.049 |
| 20 | 0.046 | 0.056 | 0.114 | 0.072 | 0.021 | 0.010 |
| 25 | 0.128 | 0.158 | 0.078 | 0.121 | 0.023 | 0.053 |
| 30 | 0.106 | 0.152 | 0.221 | 0.160 | 0.033 | 0.062 |
| 35 | 0.131 | 0.126 | 0.138 | 0.132 | 0.004 | 0.121 |
| 40 | 0.135 | 0.115 | 0.167 | 0.139 | 0.015 | 0.095 |
| 45 | 0.095 | 0.151 | 0.106 | 0.117 | 0.017 | 0.067 |
| 50 | 0.111 | 0.168 | 0.123 | 0.134 | 0.017 | 0.083 |
| 55 | 0.526 | 0.134 | 0.129 | 0.263 | 0.131 | -0.120 |
| 60 | 0.399 | 0.109 | 0.171 | 0.227 | 0.088 | -0.031 |
| 65 | 0.270 | 0.124 | 0.293 | 0.229 | 0.053 | 0.074 |
| 70 | 0.222 | 0.402 | 0.180 | 0.268 | 0.068 | 0.069 |
| 75 | 0.072 | 0.495 | 0.164 | 0.244 | 0.128 | -0.131 |
| 80 | 0.024 | 0.509 | 0.173 | 0.235 | 0.143 | -0.183 |
| 85 | 0.080 | 0.308 | 0.182 | 0.190 | 0.066 | -0.002 |
| 90 | 0.028 | 0.418 | 0.255 | 0.234 | 0.113 | -0.096 |
| 95 | 0.146 | 0.203 | 0.244 | 0.198 | 0.028 | 0.115 |
| 100 | 0.326 | 0.302 | 0.327 | 0.319 | 0.008 | 0.294 |
| 105 | 0.069 | 0.507 | 0.550 | 0.375 | 0.153 | -0.073 |
| 110 | 0.173 | 0.170 | 0.369 | 0.237 | 0.066 | 0.045 |
| 115 | 0.270 | 0.239 | 0.069 | 0.193 | 0.062 | 0.010 |
| 120 | 0.192 | 0.203 | 0.207 | 0.201 | 0.004 | 0.188 |
| 125 | 0.195 | 0.302 | 0.384 | 0.294 | 0.055 | 0.134 |
| 130 | 0.543 | 0.507 | 0.047 | 0.366 | 0.160 | -0.101 |
| 135 | 0.194 | 0.436 | 0.128 | 0.253 | 0.093 | -0.020 |
| 140 | 0.105 | 0.265 | 0.331 | 0.233 | 0.067 | 0.037 |
| 145 | 0.073 | 0.205 | 0.515 | 0.265 | 0.131 | -0.118 |
| 150 | 0.037 | 0.252 | 0.172 | 0.154 | 0.063 | -0.029 |
| 155 | 0.088 | 0.020 | 0.052 | 0.053 | 0.019 | -0.004 |
| 160 | 0.174 | 0.016 | 0.348 | 0.179 | 0.096 | -0.101 |
| 165 | 0.143 | 0.027 | 0.317 | 0.162 | 0.084 | -0.084 |
| 170 | 0.177 | 0.085 | 0.247 | 0.170 | 0.047 | 0.033 |
| 175 | 0.079 | 0.129 | 0.212 | 0.140 | 0.039 | 0.026 |
| 180 | 0.070 |  | 0.254 | 0.162 | 0.092 | -0.420 |
| 185 |  |  | 0.255 |  |  |  |
| 190 |  |  | 0.477 |  |  |  |
| 195 |  |  | 0.159 |  |  |  |
| 200 |  |  | 0.061 |  |  |  |
| **Park Headquarters (PHQ)** | | | | | | |
| cm | Core 1 | Core 2 |  | Avg | StErr | CI LB |
| 5 | 0.043 | 0.051 |  | 0.047 | 0.004 | 0.022 |
| 10 | 0.122 | 0.044 |  | 0.083 | 0.039 | -0.164 |
| 15 | 0.313 | 0.067 |  | 0.190 | 0.123 | -0.587 |
| 20 | 0.272 | 0.393 |  | 0.333 | 0.060 | -0.049 |
| 25 | 0.276 | 0.476 |  | 0.376 | 0.100 | -0.255 |
| 30 | 0.572 | 0.351 |  | 0.462 | 0.111 | -0.236 |
| 35 | 0.522 | 0.522 |  | 0.522 | 0.000 | 0.520 |
| 40 | 0.206 | 0.153 |  | 0.179 | 0.027 | 0.011 |
| 45 | 0.238 | 0.272 |  | 0.255 | 0.017 | 0.148 |
| 50 | 0.162 | 0.163 |  | 0.163 | 0.000 | 0.161 |
| 55 | 0.162 | 0.170 |  | 0.166 | 0.004 | 0.144 |
| 60 | 0.061 | 0.111 |  | 0.086 | 0.025 | -0.071 |
| 65 | 0.061 | 0.168 |  | 0.115 | 0.053 | -0.223 |
| 70 | 0.102 | 0.049 |  | 0.076 | 0.027 | -0.092 |
| 75 | 0.120 | 0.049 |  | 0.085 | 0.036 | -0.141 |
| 80 | 0.150 | 0.114 |  | 0.132 | 0.018 | 0.021 |
| 85 | 0.117 | 0.123 |  | 0.120 | 0.003 | 0.103 |
| 90 | 0.101 | 0.188 |  | 0.144 | 0.043 | -0.130 |
| 95 | 0.067 | 0.085 |  | 0.076 | 0.009 | 0.019 |
| 100 | 0.073 | 0.053 |  | 0.063 | 0.010 | 0.000 |
| 105 | 0.156 | 0.064 |  | 0.110 | 0.046 | -0.183 |
| 110 | 0.100 | 0.187 |  | 0.144 | 0.043 | -0.130 |
| 115 | 0.142 | 0.118 |  | 0.130 | 0.012 | 0.054 |
| 120 | 0.105 | 0.137 |  | 0.121 | 0.016 | 0.018 |
| 125 | 0.085 | 0.148 |  | 0.116 | 0.032 | -0.084 |
| 130 | 0.079 | 0.152 |  | 0.115 | 0.036 | -0.114 |
| 135 | 0.362 | 0.129 |  | 0.246 | 0.117 | -0.490 |
| 140 | 0.251 | 0.224 |  | 0.237 | 0.014 | 0.152 |
| 145 | 0.182 | 0.315 |  | 0.248 | 0.067 | -0.174 |
| 150 | 0.219 | 0.122 |  | 0.170 | 0.049 | -0.137 |
| 155 | 0.101 | 0.248 |  | 0.175 | 0.073 | -0.289 |
| 160 |  | 0.266 |  |  |  |  |
| 165 |  | 0.164 |  |  |  |  |
| 170 |  | 0.055 |  |  |  |  |
| 175 |  | 0.031 |  |  |  |  |
| 180 |  | 0.042 |  |  |  |  |
| 185 |  | 0.082 |  |  |  |  |
| 190 |  | 0.098 |  |  |  |  |
| **South Turning Basin (STB)** | | | | | | |
| cm | Core 1 | Core 2 |  | Avg | StErr | CI LB |
| 5 | 0.641 | 0.749 |  | 0.695 | 0.054 | 0.353 |
| 10 | 0.776 | 0.913 |  | 0.844 | 0.069 | 0.411 |
| 15 | 0.883 | 0.889 |  | 0.886 | 0.003 | 0.869 |
| 20 | 0.852 | 0.875 |  | 0.863 | 0.012 | 0.868 |
| 25 | 0.965 | 0.152 |  | 0.558 | 0.407 | -2.010 |
| 30 | 0.874 | 0.169 |  | 0.522 | 0.353 | -1.704 |
| 35 | 0.656 | 0.150 |  | 0.403 | 0.253 | -1.197 |
| 40 | 0.630 | 0.151 |  | 0.391 | 0.240 | -1.123 |
| 45 | 0.504 | 0.185 |  | 0.344 | 0.160 | -0.664 |
| 50 | 0.390 | 0.133 |  | 0.262 | 0.128 | -0.549 |
| 55 | 0.333 |  |  |  |  |  |
| 60 | 0.601 |  |  |  |  |  |
| 65 | 0.189 |  |  |  |  |  |
| 70 | 0.050 |  |  |  |  |  |
| 75 | 0.090 |  |  |  |  |  |
| **West Lake (WL)** | | | | | | |
| cm | Core 1 | Core 2 |  | Avg | StErr | CI LB |
| 5 | 0.152 | 0.178 |  | 0.165 | 0.013 | 0.083 |
| 10 | 0.145 | 0.135 |  | 0.140 | 0.005 | 0.109 |
| 15 | 0.195 | 0.064 |  | 0.130 | 0.065 | -0.283 |
| 20 | 0.250 | 0.097 |  | 0.174 | 0.076 | -0.308 |
| 25 | 0.127 | 0.111 |  | 0.119 | 0.008 | 0.068 |
| 30 | 0.099 | 0.113 |  | 0.106 | 0.007 | 0.060 |
| 35 | 0.172 | 0.183 |  | 0.178 | 0.006 | 0.142 |
| 40 | 0.119 | 0.276 |  | 0.197 | 0.079 | -0.299 |
| 45 | 0.313 | 0.230 |  | 0.272 | 0.042 | 0.008 |
| 50 | 0.284 | 0.265 |  | 0.274 | 0.009 | 0.215 |
| 55 | 0.236 | 0.185 |  | 0.211 | 0.026 | 0.050 |
| 60 | 0.148 | 0.150 |  | 0.149 | 0.001 | 0.145 |
| 65 | 0.117 | 0.154 |  | 0.136 | 0.019 | 0.018 |
| 70 | 0.141 | 0.157 |  | 0.149 | 0.008 | 0.097 |
| 75 | 0.134 | 0.242 |  | 0.188 | 0.054 | -0.154 |
| 80 | 0.045 | 0.093 |  | 0.069 | 0.024 | -0.083 |
| 85 | 0.038 | 0.110 |  | 0.074 | 0.036 | -0.155 |
| 90 | 0.063 | 0.026 |  | 0.045 | 0.019 | -0.073 |
| **North Reef (NR)** | | | | | | |
| cm | NR 1 | NR 2 | NR 3 | Avg | StErr | CI LB |
| 5 | 0.048 | 0.069 | 0.057 | 0.058 | 0.006 | 0.040 |
| **South Reef (SR)** | | | | | | |
| cm | SR 1 | SR 2 | SR 3 | Avg | StErr | CI LB |
| 5 | 0.130 | 0.060 | 0.063 | 0.085 | 0.023 | 0.017 |

Bolded numbers (PLI > 1) indicate pollution is present. Avg = average; StErr = standard error; CI LB = confidence interval lower bound.
