# Supplementary material for: Assessing the ecological risk of heavy metal sediment contamination from Port Everglades Florida USA
Source: PeerJ. 2023 Nov 14;11:e16152. doi: 10.7717/peerj.16152 (PMC10655720; doi:10.7717/peerj.16152)
Supplement: Supplemental Information 22 — Orange values are above threshold effect level (TEL 7.24 µg/g) and red above probable effect level (PEL 41.6 µg/g). Avg = average; StErr = standard error; CI LB = confidence interval lower bound. [file peerj-11-16152-s022.docx]

**Table S21**. Arsenic concentrations (µg/g) and statistical analyses for all cores and sediments per depth.

| **Dania Cut-off Canal (DCC)** | | | | | | |
| --- | --- | --- | --- | --- | --- | --- |
| cm | DCC 1  As | DCC 2  As | DCC 3  As | Avg | StErr | CI LB |
| 5 | 36.4 | 0.803 | 18.1 | 18.5 | 10.3 | -11.6 |
| 10 | 11.4 | 44.3 | 21.2 | 25.6 | 9.75 | -2.83 |
| 15 | 42.9 | 41.3 | 52.2 | 45.5 | 3.40 | 35.6 |
| 20 | 66.2 | 56.0 | 10.6 | 44.3 | 17.1 | -5.69 |
| 25 | 73.2 | 46.5 | 22.0 | 47.2 | 14.8 | 4.13 |
| 30 | 53.2 | 72.6 | 51.1 | 58.9 | 6.83 | 40.0 |
| 35 | 36.3 | 52.8 | 114 | 67.7 | 23.6 | -1.24 |
| 40 | 61.2 | 80.6 | 54.3 | 65.3 | 7.87 | 42.4 |
| 45 | 26.7 | 69.6 | 223 | 107 | 59.6 | -67.6 |
| 50 | 12.6 | 126. | 17.8 | 52.2 | 37.0 | -55.8 |
| 55 | 13.5 | 17.6 | 16.2 | 15.8 | 1.19 | 12.3 |
| 60 | 13.9 | 12.8 | 6.3 | 11.0 | 2.37 | 4.11 |
| 65 | 12.2 | 16.3 | 8.1 | 12.2 | 2.36 | 5.33 |
| 70 | 17.9 | 55.0 | 8.7 | 27.2 | 14.2 | -14.1 |
| 75 | 15.0 | 7.79 | 14.0 | 12.3 | 2.25 | 5.69 |
| 80 |  | 10.3 | 13.3 | 11.8 | 1.46 | 2.58 |
| 85 |  | 14.3 | 10.1 | 12.2 | 2.07 | -0.880 |
| 90 |  | 13.8 | 12.3 | 13.0 | 0.75 | 8.27 |
| 95 |  |  | 12.5 |  |  |  |
| 100 |  |  | 25.6 |  |  |  |
| **Park Education Center (PEC)** | | | | | | |
| cm | PEC 1  As | PEC 2  As | PEC 3  As | Avg | StErr | CI LB |
| 5 | 1.34 | 18.9 | 22.2 | 14.1 | 6.47 | -4.75 |
| 10 | 0.641 | 1.56 | 3.75 | 1.98 | 0.92 | -0.701 |
| 15 | 2.01 | 0.61 | 1.23 | 1.28 | 0.41 | 0.09 |
| 20 | 0.775 | 0.753 | 1.00 | 0.84 | 0.08 | 0.610 |
| 25 | 2.81 | 2.61 | 1.04 | 2.15 | 0.56 | 0.510 |
| 30 | 3.93 | 3.21 | 3.56 | 3.57 | 0.21 | 2.96 |
| 35 | 2.94 | 2.45 | 0.98 | 2.12 | 0.59 | 0.403 |
| 40 | 4.54 | 2.18 | 4.12 | 3.61 | 0.73 | 1.49 |
| 45 | 2.97 | 4.64 | 2.25 | 3.28 | 0.71 | 1.21 |
| 50 | 4.42 | 4.21 | 3.78 | 4.14 | 0.19 | 3.59 |
| 55 | 12.1 | 3.43 | 2.49 | 6.00 | 3.05 | -2.91 |
| 60 | 12.1 | 3.59 | 3.78 | 6.49 | 2.81 | -1.71 |
| 65 | 6.16 | 3.29 | 4.33 | 4.59 | 0.84 | 2.14 |
| 70 | 5.62 | 8.94 | 3.97 | 6.18 | 1.46 | 1.91 |
| 75 | 2.10 | 10.4 | 3.09 | 5.19 | 2.62 | -2.44 |
| 80 | 1.13 | 8.85 | 3.32 | 4.43 | 2.30 | -2.27 |
| 85 | 3.24 | 8.08 | 4.01 | 5.11 | 1.50 | 0.723 |
| 90 | 0.85 | 8.24 | 6.55 | 5.21 | 2.24 | -1.31 |
| 95 | 5.76 | 2.19 | 6.60 | 4.85 | 1.35 | 0.90 |
| 100 | 11.1 | 1.09 | 8.32 | 6.82 | 2.98 | -1.87 |
| 105 | 2.80 | 0.80 | 14.4 | 5.99 | 4.23 | -6.36 |
| 110 | 6.99 | 5.10 | 7.14 | 6.41 | 0.66 | 4.49 |
| 115 | 11.6 | 11.5 | 1.50 | 8.20 | 3.35 | -1.59 |
| 120 | 15.4 | 8.09 | 5.88 | 9.80 | 2.88 | 1.38 |
| 125 | 13.5 | 10.6 | 12.0 | 12.1 | 0.84 | 9.59 |
| 130 | 32.1 | 62.4 | 1.21 | 31.9 | 17.67 | -19.7 |
| 135 | 15.8 | 22.0 | 3.34 | 13.7 | 5.49 | -2.32 |
| 140 | 7.32 | 8.27 | 10.9 | 8.84 | 1.08 | 5.69 |
| 145 | 3.82 | 6.75 | 27.8 | 12.8 | 7.56 | -9.28 |
| 150 | 1.49 | 12.22 | 5.29 | 6.33 | 3.14 | -2.83 |
| 155 | 0.88 | 1.25 | 1.41 | 1.18 | 0.16 | 0.723 |
| 160 | 4.01 | 0.80 | 18.9 | 7.89 | 5.56 | -8.35 |
| 165 | 2.87 | 1.45 | 15.4 | 6.56 | 4.42 | -6.34 |
| 170 | 2.05 | 3.35 | 10.5 | 5.30 | 2.63 | -2.38 |
| 175 | 1.11 | 3.86 | 7.43 | 4.13 | 1.83 | -1.21 |
| 180 | 0.94 |  | 10.7 | 5.80 | 4.86 | -24.9 |
| 185 |  |  | 9.72 |  |  |  |
| 190 |  |  | 31.5 |  |  |  |
| 195 |  |  | 6.81 |  |  |  |
| 200 |  |  | 1.14 |  |  |  |
| **Park Headquarters (PHQ)** | | | | | | |
| cm | PHQ 1  As | PHQ 2  As |  | Avg | StErr | CI LB |
| 5 | 0.96 | 0.93 |  | 0.95 | 0.01 | 0.870 |
| 10 | 2.86 | 1.05 |  | 1.95 | 0.90 | -3.76 |
| 15 | 11.3 | 0.77 |  | 6.03 | 5.26 | -27.2 |
| 20 | 11.4 | 14.4 |  | 12.9 | 1.50 | 3.41 |
| 25 | 21.8 | 24.4 |  | 23.1 | 1.32 | 14.8 |
| 30 | 75.0 | 17.8 |  | 46.4 | 28.61 | -134 |
| 35 | 40.6 | 26.4 |  | 33.5 | 7.14 | -11.6 |
| 40 | 10.8 | 11.1 |  | 10.9 | 0.17 | 9.89 |
| 45 | 15.1 | 32.5 |  | 23.8 | 8.67 | -31.0 |
| 50 | 11.9 | 11.5 |  | 11.7 | 0.24 | 10.2 |
| 55 | 15.4 | 11.0 |  | 13.2 | 2.19 | -0.63 |
| 60 | 6.04 | 9.53 |  | 7.78 | 1.74 | -3.23 |
| 65 | 5.48 | 10.6 |  | 8.02 | 2.54 | -8.00 |
| 70 | 7.85 | 5.60 |  | 6.73 | 1.13 | -0.38 |
| 75 | 10.1 | 5.58 |  | 7.86 | 2.28 | -6.52 |
| 80 | 10.9 | 14.2 |  | 12.5 | 1.68 | 1.95 |
| 85 | 11.51 | 12.7 |  | 12.0 | 0.58 | 8.41 |
| 90 | 11.6 | 20.0 |  | 15.8 | 4.16 | -10.5 |
| 95 | 9.29 | 13.5 |  | 11.4 | 2.13 | -2.02 |
| 100 | 6.98 | 11.6 |  | 9.30 | 2.32 | -5.33 |
| 105 | 17.5 | 11.1 |  | 14.3 | 3.20 | -5.86 |
| 110 | 14.5 | 19.5 |  | 17.0 | 2.50 | 1.21 |
| 115 | 19.1 | 14.0 |  | 16.5 | 2.58 | 0.21 |
| 120 | 18.4 | 30.1 |  | 24.2 | 5.86 | -12.8 |
| 125 | 13.7 | 30.4 |  | 22.1 | 8.37 | -30.8 |
| 130 | 8.26 | 22.6 |  | 15.4 | 7.17 | -29.9 |
| 135 | 16.6 | 23.9 |  | 20.3 | 3.65 | -2.82 |
| 140 | 22.4 | 20.1 |  | 21.3 | 1.14 | 14.0 |
| 145 | 18.1 | 20.6 |  | 19.4 | 1.26 | 11.4 |
| 150 | 16.9 | 8.27 |  | 12.6 | 4.33 | -14.7 |
| 155 | 7.58 | 15.6 |  | 11.6 | 4.00 | -13.7 |
| 160 |  | 19.5 |  |  |  |  |
| 165 |  | 12.8 |  |  |  |  |
| 170 |  | 5.01 |  |  |  |  |
| 175 |  | 5.22 |  |  |  |  |
| 180 |  | 3.96 |  |  |  |  |
| 185 |  | 4.99 |  |  |  |  |
| 190 |  | 4.14 |  |  |  |  |
| **South Turning Basin (STB)** | | | | | | |
| cm | STB 1  As | STB 2  As |  | Avg | StErr | CI LB |
| 5 | 21.5 | 15.5 |  | 18.5 | 2.98 | -0.331 |
| 10 | 59.9 | 15.2 |  | 37.5 | 22.4 | -104 |
| 15 | 21.5 | 13.9 |  | 17.7 | 3.77 | -6.10 |
| 20 | 21.0 | 15.6 |  | 18.3 | 2.69 | 1.25 |
| 25 | 22.9 | 9.11 |  | 16.0 | 6.87 | -27.4 |
| 30 | 37.7 | 4.73 |  | 21.2 | 16.5 | -82.8 |
| 35 | 31.7 | 12.6 |  | 22.1 | 9.54 | -38.1 |
| 40 | 21.3 | 14.5 |  | 17.9 | 3.39 | -3.51 |
| 45 | 16.6 | 14.5 |  | 15.5 | 1.04 | 8.94 |
| 50 | 18.7 | 13.3 |  | 16.0 | 2.70 | -1.04 |
| 55 | 15.7 |  |  |  |  |  |
| 60 | 21.3 |  |  |  |  |  |
| 65 | 14.6 |  |  |  |  |  |
| 70 | 3.01 |  |  |  |  |  |
| 75 | 4.78 |  |  |  |  |  |
| **West Lake (WL)** | | | | | | |
| cm | WL 1  As | WL 2  As |  | Avg | StErr | CI LB |
| 5 | 8.12 | 6.42 |  | 7.27 | 0.85 | 1.91 |
| 10 | 9.04 | 5.23 |  | 7.13 | 1.90 | -4.89 |
| 15 | 8.38 | 1.68 |  | 5.03 | 3.35 | -16.1 |
| 20 | 6.22 | 3.54 |  | 4.88 | 1.34 | -3.56 |
| 25 | 5.28 | 2.99 |  | 4.14 | 1.14 | -3.09 |
| 30 | 4.63 | 3.25 |  | 3.94 | 0.69 | -0.39 |
| 35 | 4.21 | 11.0 |  | 7.59 | 3.38 | -13.8 |
| 40 | 2.96 | 12.3 |  | 7.65 | 4.69 | -22.0 |
| 45 | 10.4 | 11.2 |  | 10.8 | 0.40 | 8.32 |
| 50 | 12.5 | 12.6 |  | 12.5 | 0.04 | 12.3 |
| 55 | 10.8 | 10.6 |  | 10.7 | 0.11 | 10.0 |
| 60 | 10.3 | 10.5 |  | 10.4 | 0.12 | 9.67 |
| 65 | 11.7 | 13.8 |  | 12.8 | 1.06 | 6.04 |
| 70 | 14.0 | 11.0 |  | 12.5 | 1.49 | 3.08 |
| 75 | 12.1 | 12.9 |  | 12.5 | 0.42 | 9.85 |
| 80 | 4.85 | 21.7 |  | 13.3 | 8.42 | -39.9 |
| 85 | 2.60 | 8.75 |  | 5.68 | 3.07 | -13.7 |
| 90 | 3.18 | 4.62 |  | 3.90 | 0.72 | -0.65 |
| **North Reef (NR)** | | | | | | |
| cm | NRF 1  As | NRF 2  As | NRF 2  As | Avg | StErr | CI LB |
| 5 | 6.89 | 6.37 | 8.55 | 7.27 | 0.66 | 5.35 |
| **South Reef (SR)** | | | | | | |
| cm | SRF 1  As | SRF 2  As | SRF 2  As | Avg | StErr | CI LB |
| 5 | 2.73 | 3.82 | 3.32 | 3.29 | 0.32 | 2.37 |

Orange values are above threshold effect level (TEL 7.24 µg/g) and red above probable effect level (PEL 41.6 µg/g). Avg = average; StErr = standard error; CI LB = confidence interval lower bound.
