# Supplementary material for: Assessing the ecological risk of heavy metal sediment contamination from Port Everglades Florida USA
Source: PeerJ. 2023 Nov 14;11:e16152. doi: 10.7717/peerj.16152 (PMC10655720; doi:10.7717/peerj.16152)
Supplement: Supplemental Information 23 — PER > 320 significantly high ecological risk (dark red), 160 < PER < 320 high ecological risk (red), 80 < PER < 160 considerable ecological risk (orange), 40 < PER < 80 moderate ecological risk (yellow), PER < 40 low ecological risk. Avg = average; StErr = standard error; CI LB = confidence interval lower bound. [file peerj-11-16152-s023.docx]

**Table S22**. Potential ecological risk (PER) and statistical analyses for all cores and sediments per depth.

| **Dania Cut-off Canal (DCC)** | | | | | | |
| --- | --- | --- | --- | --- | --- | --- |
| cm | Core 1 | Core 2 | Core 3 | Avg | StErr | CI LB |
| 5 | 364 | 9.9 | 160 | 178 | 103 | -122 |
| 10 | 125 | 449 | 204 | 259 | 97.4 | -25.1 |
| 15 | 346 | 406 | 360 | 371 | 18.0 | 318 |
| 20 | 527 | 543 | 147 | 406 | 129 | 28.0 |
| 25 | 590 | 439 | 176 | 402 | 121 | 48.6 |
| 30 | 417 | 642 | 392 | 484 | 79.4 | 252 |
| 35 | 293 | 466 | 772 | 510 | 140 | 102 |
| 40 | 487 | 669 | 386 | 514 | 82.9 | 272 |
| 45 | 205 | 556 | 1503 | 755 | 387 | -377 |
| 50 | 92.9 | 1006 | 128 | 409 | 299 | -463 |
| 55 | 94.2 | 127 | 113 | 111 | 9.5 | 83.7 |
| 60 | 97.3 | 89.0 | 43.3 | 76.5 | 16.8 | 27.6 |
| 65 | 85.4 | 119 | 55.1 | 86.5 | 18.4 | 32.6 |
| 70 | 136 | 446 | 61.2 | 214 | 118. | -130 |
| 75 | 107 | 55.4 | 93.9 | 85.5 | 15.5 | 40.1 |
| 80 |  | 72.5 | 89.5 | 81.0 | 8.5 | 27.1 |
| 85 |  | 95.6 | 70.9 | 83.2 | 12.4 | 5.2 |
| 90 |  | 96.3 | 82.5 | 89.4 | 6.9 | 45.9 |
| 95 |  |  | 86.1 |  |  |  |
| 100 |  |  | 180 |  |  |  |
| **Park Education Center (PEC)** | | | | | | |
| cm | Core 1 | Core 2 | Core 3 | Avg | StErr | CI LB |
| 5 | 21.3 | 441 | 332 | 265 | 126. | -103 |
| 10 | 14.8 | 33.8 | 54.3 | 34.3 | 11.4 | 1.0 |
| 15 | 34.6 | 15.4 | 21.3 | 23.8 | 5.7 | 7.1 |
| 20 | 16.7 | 18.9 | 18.1 | 17.9 | 0.6 | 16.1 |
| 25 | 38.7 | 41.3 | 19.8 | 33.3 | 6.8 | 13.5 |
| 30 | 38.8 | 47.6 | 50.0 | 45.5 | 3.4 | 35.5 |
| 35 | 36.6 | 36.3 | 27.3 | 33.4 | 3.0 | 24.5 |
| 40 | 50.2 | 34.3 | 45.6 | 43.3 | 4.7 | 29.6 |
| 45 | 29.8 | 50.6 | 26.7 | 35.7 | 7.5 | 13.9 |
| 50 | 48.1 | 54.5 | 36.2 | 46.3 | 5.4 | 30.6 |
| 55 | 203. | 40.0 | 33.6 | 92.1 | 55.4 | -69.5 |
| 60 | 195 | 36.4 | 42.5 | 91.4 | 51.9 | -60.3 |
| 65 | 116 | 41.5 | 57.8 | 71.8 | 22.6 | 5.7 |
| 70 | 117 | 131 | 50.7 | 99.6 | 24.8 | 27.3 |
| 75 | 45.0 | 185 | 38.5 | 89.4 | 47.7 | -49.8 |
| 80 | 16.5 | 172 | 43.3 | 77.4 | 48.1 | -63.1 |
| 85 | 32.4 | 146 | 46.0 | 74.7 | 35.7 | -29.5 |
| 90 | 9.8 | 198 | 67.2 | 91.5 | 55.5 | -70.6 |
| 95 | 53.5 | 41.1 | 66.7 | 53.8 | 7.4 | 32.2 |
| 100 | 122 | 19.2 | 93.5 | 78.2 | 30.6 | -11.1 |
| 105 | 27.6 | 13.8 | 183 | 74.8 | 54.2 | -83.5 |
| 110 | 67.5 | 50.8 | 143 | 86.8 | 28.1 | 4.7 |
| 115 | 108 | 102. | 25.5 | 78.3 | 26.4 | 1.1 |
| 120 | 125 | 75.6 | 79.3 | 93.4 | 16.0 | 46.7 |
| 125 | 118 | 99.8 | 123 | 114 | 7.1 | 92.9 |
| 130 | 501 | 492 | 15.2 | 336 | 160.4 | -133 |
| 135 | 140 | 182 | 35.9 | 119 | 43.4 | -7.5 |
| 140 | 60.1 | 78.9 | 108 | 82.4 | 14.0 | 41.6 |
| 145 | 34.7 | 68.1 | 240 | 114 | 63.5 | -71.2 |
| 150 | 15.3 | 111. | 53.0 | 59.7 | 27.7 | -21.3 |
| 155 | 12.6 | 11.8 | 13.0 | 12.5 | 0.3 | 11.5 |
| 160 | 42.3 | 7.0 | 154 | 67.9 | 44.4 | -61.8 |
| 165 | 33.2 | 14.1 | 136 | 61.0 | 37.7 | -49.2 |
| 170 | 25.0 | 31.1 | 106 | 54.1 | 26.1 | -22.1 |
| 175 | 19.4 | 41.0 | 80.8 | 47.0 | 18.0 | -5.5 |
| 180 | 15.4 |  | 99.6 | 57.5 | 42.1 | -208 |
| 185 |  |  | 99.2 |  |  |  |
| 190 |  |  | 288 |  |  |  |
| 195 |  |  | 62.7 |  |  |  |
| 200 |  |  | 11.1 |  |  |  |
| **Park Headquarters (PHQ)** | | | | | | |
| cm | Core 1 | Core 2 |  | Avg | StErr | CI LB |
| 5 | 12.3 | 11.8 |  | 12.1 | 0.2 | 10.5 |
| 10 | 33.6 | 10.6 |  | 22.1 | 11.5 | -50.3 |
| 15 | 111 | 11.2 |  | 60.9 | 49.7 | -253. |
| 20 | 119 | 151 |  | 135 | 16.1 | 33.0 |
| 25 | 179 | 319 |  | 249 | 70.1 | -194 |
| 30 | 562 | 292 |  | 427 | 135. | -426 |
| 35 | 339 | 398 |  | 369 | 29.7 | 181 |
| 40 | 93.6 | 128. |  | 111 | 17.0 | 3.5 |
| 45 | 123 | 242 |  | 182 | 59.6 | -194. |
| 50 | 88.6 | 125 |  | 107 | 18.1 | -7.4 |
| 55 | 115 | 129 |  | 122 | 6.8 | 79.1 |
| 60 | 45.0 | 84.9 |  | 65.0 | 19.9 | -60.8 |
| 65 | 42.6 | 114. |  | 78.4 | 35.8 | -148. |
| 70 | 71.2 | 41.3 |  | 56.2 | 15.0 | -38.2 |
| 75 | 90.7 | 38.9 |  | 64.8 | 25.9 | -98.6 |
| 80 | 107 | 102 |  | 105 | 2.6 | 88.2 |
| 85 | 113 | 95.5 |  | 104 | 8.7 | 49.0 |
| 90 | 99.5 | 148. |  | 124 | 24.0 | -28.2 |
| 95 | 76.7 | 94.6 |  | 85.7 | 9.0 | 29.1 |
| 100 | 60.1 | 79.7 |  | 69.9 | 9.8 | 8.1 |
| 105 | 152 | 77.3 |  | 114 | 37.1 | -120 |
| 110 | 114 | 190 |  | 152 | 38.1 | -88.6 |
| 115 | 164 | 116 |  | 140 | 24.1 | -12.1 |
| 120 | 140 | 214 |  | 177 | 37.4 | -59.4 |
| 125 | 108 | 221 |  | 165 | 56.6 | -193 |
| 130 | 66.7 | 176 |  | 121 | 54.4 | -222 |
| 135 | 117 | 182 |  | 150 | 32.4 | -55.3 |
| 140 | 171 | 146 |  | 159 | 12.5 | 79.5 |
| 145 | 131 | 156 |  | 143 | 12.3 | 65.8 |
| 150 | 122 | 69.1 |  | 95.8 | 26.7 | -72.6 |
| 155 | 54.7 | 118 |  | 86.5 | 31.8 | -115. |
| 160 |  | 15.0 |  |  |  |  |
| 165 |  | 94.7 |  |  |  |  |
| 170 |  | 35.7 |  |  |  |  |
| 175 |  | 35.8 |  |  |  |  |
| 180 |  | 28.1 |  |  |  |  |
| 185 |  | 38.5 |  |  |  |  |
| 190 |  | 31.6 |  |  |  |  |
| **South Turning Basin (STB)** | | | | | | |
| cm | Core 1 | Core 2 |  | Avg | StErr | CI LB |
| 5 | 228 | 220 |  | 224 | 4.4 | 196 |
| 10 | 491 | 246 |  | 368 | 122. | -404 |
| 15 | 284 | 243 |  | 263 | 20.9 | 132 |
| 20 | 283 | 140 |  | 212 | 71.5 | 133 |
| 25 | 278 | 74.6 |  | 176 | 102. | -465 |
| 30 | 362 | 54.4 |  | 208 | 154. | -763 |
| 35 | 272 | 97.8 |  | 185 | 87.1 | -365 |
| 40 | 207 | 98.1 |  | 153 | 54.4 | -191 |
| 45 | 142 | 112 |  | 127 | 15.0 | 32.0 |
| 50 | 167 | 108 |  | 138 | 29.5 | -48.6 |
| 55 | 129 |  |  |  |  |  |
| 60 | 209 |  |  |  |  |  |
| 65 | 113 |  |  |  |  |  |
| 70 | 22.4 |  |  |  |  |  |
| 75 | 37.6 |  |  |  |  |  |
| **West Lake (WL)** | | | | | | |
| cm | Core 1 | Core 2 |  | Avg | StErr | CI LB |
| 5 | 75.5 | 82.6 |  | 79.0 | 3.5 | 56.8 |
| 10 | 88.6 | 62.6 |  | 75.6 | 13.0 | -6.6 |
| 15 | 90.2 | 22.5 |  | 56.3 | 33.8 | -157 |
| 20 | 88.8 | 40.7 |  | 64.8 | 24.1 | -87.2 |
| 25 | 65.8 | 45.9 |  | 55.8 | 10.0 | -7.2 |
| 30 | 48.2 | 47.3 |  | 47.7 | 0.5 | 44.8 |
| 35 | 59.8 | 133 |  | 96.4 | 36.7 | -135 |
| 40 | 44.7 | 154 |  | 99.2 | 54.5 | -245 |
| 45 | 165 | 109 |  | 137 | 28.1 | -40.9 |
| 50 | 125 | 128 |  | 126 | 1.2 | 119 |
| 55 | 108 | 92.7 |  | 100 | 7.4 | 53.3 |
| 60 | 86.1 | 84.7 |  | 85.4 | 0.7 | 81.1 |
| 65 | 87.5 | 112 |  | 99.5 | 12.0 | 23.7 |
| 70 | 110. | 93.2 |  | 101 | 8.2 | 49.4 |
| 75 | 94.1 | 97.7 |  | 95.9 | 1.8 | 84.5 |
| 80 | 35.9 | 149. |  | 92.5 | 56.6 | -265 |
| 85 | 19.5 | 70.1 |  | 44.8 | 25.3 | -115 |
| 90 | 25.0 | 31.3 |  | 28.2 | 3.1 | 8.4 |
| **North Reef (NR)** | | | | | | |
| cm | NR 1 | NR 2 | NR 3 | Avg | StErr | CI LB |
| 5 | 49.8 | 52.9 | 70.4 | 57.7 | 6.4 | 39.0 |
| **South Reef (SR)** | | | | | | |
| cm | SR 1 | SR 2 | SR 3 | Avg | StErr | CI LB |
| 5 | 62.2 | 35.8 | 35.7 | 44.6 | 8.8 | 18.8 |

PER > 320 significantly high ecological risk (dark red), 160 < PER < 320 high ecological risk (red), 80 < PER < 160 considerable ecological risk (orange), 40 < PER < 80 moderate ecological risk (yellow), PER < 40 low ecological risk. Avg = average; StErr = standard error; CI LB = confidence interval lower bound.
