# Supplementary material for: Assessing the ecological risk of heavy metal sediment contamination from Port Everglades Florida USA
Source: PeerJ. 2023 Nov 14;11:e16152. doi: 10.7717/peerj.16152 (PMC10655720; doi:10.7717/peerj.16152)
Supplement: Supplemental Information 24 — Sites that only had significant differences are shown [file peerj-11-16152-s024.docx]

**Table S23**. P-Value, Matched-Pairs T-Test with B-H Correction statistics depicting differences between duplicate cores per location when comparing to geo-accumulation and pollution ecological risk indices.

| **Dania Cut-off Canal (DCC)**  **Geo-accumulation Index** | | | | | |
| --- | --- | --- | --- | --- | --- |
|  | Core 1 vs Core 2 | Core 2 vs Core 3 | Core 1 vs Core 3 | Sig @ Site? |  |
| **Cd** | 0.0354 | 0.0131 | 0.0125 | Yes |  |
|  | | | | | |
| **South Turning Basin (STB)**  **Geo-accumulation Index** | | | | | |
|  | Core 1 vs Core 2 |  | | Sig @ Site? |  |
| **Mo** | 0.0049 |  |  | Yes |  |
| **Zn** | 0.0437 |  |  | Yes |  |
| **Cu** | 0.0170 |  |  | Yes |  |
| **As** | 0.0047 |  |  | Yes |  |
|  | | | | | |
| **South Turning Basin (STB)**  **Potential Ecological Risk** | | | | | |
|  | Core 1 vs Core 2 |  | | Sig @ Site? |  |
|  | 0.0060 |  |  | Yes |  |

Sites that only had significant differences are shown.
