# Supplementary material for: Assessing the ecological risk of heavy metal sediment contamination from Port Everglades Florida USA
Source: PeerJ. 2023 Nov 14;11:e16152. doi: 10.7717/peerj.16152 (PMC10655720; doi:10.7717/peerj.16152)
Supplement: Supplemental Information 25 — * port; # location with contaminated acid mine waste; n/d = non-detected. [file peerj-11-16152-s025.docx]

**Table S24**: Marine sediment heavy metal concentration ranges in Port Everglades, FL, worldwide ports and estuaries.

| Heavy Metals | Concentrations  µg/g | Location | References |
| --- | --- | --- | --- |
| As | 4 – 29 | New South Wales, Australia* | (Jahan and Strezov, 2018) |
|  | 6.7 – 19.9 | Koper, Slovenia* | (Šmuc et al., 2018) |
|  | 8.0 – 21.0 | Naples, Italy* | (Adamo et al., 2005) |
|  | 107 - 220 | Estaque, France* | (Mamindy-Pajany et al., 2013) |
|  | 10 - 12.1 | Saint Mandrier, France* | (Mamindy-Pajany et al., 2013) |
|  | 4.8 – 1740^#^ | 19 England estuaries sites | (Kennish, 1997) |
|  | **0.607 – 223** | **Port Everglades, Florida, USA*** | **This Study** |
|  | **1.70 – 21.7** | **West Lake, FL, USA** | **This Study** |
|  | **2.41 – 8.55** | **Reef Sites Fort Lauderdale, FL** | **This Study** |
| Cd | 0.1 – 0.4 | Koper, Slovenia* | (Šmuc, 2018) |
|  | 0.09 – 0.47 | Laizhou Bay and Zhangzi Island, China | (Zhuang and Gao, 2014) |
|  | 0.2 – 2.5 | Naples, Italy* | (Adamo et al., 2005) |
|  | 0.3-0.4 | Estaque, France* | (Mamindy-Pajany et al., 2013) |
|  | 0.08 – 2.17 | 19 England estuaries sites | (Kennish, 1997) |
|  | 0.04 - 998 | 52 coastal world sites | (Qian et al., 2015) |
|  | **n/d – 0.916** | **Fort Lauderdale, Florida, USA*** | **This Study** |
|  | **n/d – 0.282** | **West Lake, FL, USA** | **This Study** |
|  | **0.010 – 0.039** | **Reef Sites Fort Lauderdale, FL** | **This Study** |
| Cr | 1 – 31 | New South Wales, Australia* | (Jahan and Strezov, 2018) |
|  | 8.4 – 90.4 | Laizhou Bay and Zhangzi Island, China | (Zhuang and Gao, 2014) |
|  | 10.3 – 162 | Naples, Italy* | (Adamo et al., 2005) |
|  | 24 – 207 | 19 England estuaries sites | (Kennish, 1997) |
|  | 1.0 – 463 | 52 coastal world sites | (Qian et al., 2015) |
|  | 23. – 1467 | 40 U.S. estuaries sites | (Kennish, 1997) |
|  | **0.155 – 56.8** | **Fort Lauderdale, Florida, USA*** | **This Study** |
|  | **0.337 – 11.2** | **West Lake, FL, USA** | **This Study** |
|  | **4.70 – 7.55** | **Reef Sites Fort Lauderdale, FL** | **This Study** |
| Co | 1 – 12 | New South Wales, Australia* | (Jahan and Strezov, 2018) |
|  | 1.9 – 7.2 | Naples, Italy* | (Adamo et al., 2005) |
|  | 6 - 26 | 19 England estuaries sites | (Kennish, 1997) |
|  | **0.024 – 7.40** | **Fort Lauderdale, Florida, USA*** | **This Study** |
|  | **0.119 – 0.520** | **West Lake, FL, USA** | **This Study** |
|  | **0.030 – 0.100** | **Reef Sites Fort Lauderdale, FL** | **This Study** |
| Cu | 2 – 1195 | New South Wales, Australia* | (Jahan and Strezov, 2018) |
|  | 17.6 – 37.8 | Koper, Slovenia* | (Šmuc et al., 2018) |
|  | 2.9 – 28.7 | Laizhou Bay and Zhangzi Island, China | (Zhuang and Gao, 2014) |
|  | 40 – 415 | Naples, Italy* | (Adamo et al., 2005) |
|  | 273 - 278 | Estaque, France* | (Mamindy-Pajany et al., 2013) |
|  | 194 - 220 | Saint Mandrier, France* | (Mamindy-Pajany et al., 2013) |
|  | 7 – 2398^#^ | 19 England estuaries sites | (Kennish, 1997) |
|  | 0.5 - 604 | 52 coastal world sites | (Qian et al., 2015) |
|  | 0.06 – 21 | 40 U.S. estuaries sites | (Kennish, 1997) |
|  | **0.004 – 215** | **Fort Lauderdale, Florida, USA*** | **This Study** |
|  | **0.260 – 30.4** | **West Lake, FL, USA** | **This Study** |
|  | **0.510 – 28.6** | **Reef Sites Fort Lauderdale, FL** | **This Study** |
| Pb | 2 – 165 | New South Wales, Australia* | (Jahan and Strezov, 2018) |
|  | 10.7 – 30.2 | Koper, Slovenia* | (Šmuc et al., 2018) |
|  | 6.7 – 34.0 | Laizhou Bay and Zhangzi Island, China | (Zhuang and Gao, 2014) |
|  | 37 – 314 | Naples, Italy* | (Adamo et al., 2005) |
|  | 329 - 412 | Estaque, France* | (Mamindy-Pajany et al., 2013) |
|  | 108 - 110 | Saint Mandrier, France* | (Mamindy-Pajany et al., 2013) |
|  | 20 - 2753 | 19 England estuaries sites | (Kennish, 1997) |
|  | 3 - 2360 | 52 coastal world sites | (Qian et al., 2015) |
|  | 2.17 – 186 | 40 U.S. estuaries sites | (Kennish, 1997) |
|  | **0.0169 – 73.8** | **Fort Lauderdale, Florida, USA*** | **This Study** |
|  | **0.14 – 16.4** | **West Lake, FL, USA** | **This Study** |
|  | **0.900 – 1.80** | **Reef Sites Fort Lauderdale, FL** | **This Study** |
| Mn | 6 – 201 | New South Wales, Australia* | (Jahan and Strezov, 2018) |
|  | 95 – 535 | Naples, Italy* | (Adamo et al., 2005) |
|  | 316.6 – 326 | Persian Gulf, Iran* | {Abdollahi et al., 2013) |
|  | 185 - 1169 | 19 England estuaries sites | (Kennish, 1997) |
|  | 0.4 - 4643 | 52 coastal world sites | (Qian et al., 2015) |
|  | **1.61 – 204** | **Fort Lauderdale, Florida, USA*** | **This Study** |
|  | **9.38 - 93.8** | **West Lake, FL, USA** | **This Study** |
|  | **10.1 – 24.1** | **Reef Sites Fort Lauderdale, FL** | **This Study** |
| Hg | 0.5 – 2.73 | Persian Gulf, Iran* | (Abdollahi et al., 2013) |
|  | 0.005 – 0.31 | District of Klang, Malaysia | (Tavakoly et al., 2012) |
|  | 0.018 – 0.536 | South Korea* | (Choi et al., 2011) |
|  | 0.03 – 3.01 | 19 England estuaries sites | (Kennish, 1997) |
|  | 0.01-1.8 | 52 selected coastal world sites | (Qian et al., 2015) |
|  | 0.01 – 2.34 | 40 U.S. estuaries sites | (Kennish, 1997) |
|  | **n/d – 0.736** | **Fort Lauderdale, Florida, USA*** | **This Study** |
|  | **n/d – 0.262** | **West Lake, FL, USA** | **This Study** |
|  | **n/d -0.046** | **Reef Sites Fort Lauderdale, FL** | **This Study** |
| Mo | 40 | New South Wales, Australia* | (Jahan and Strezov, 2018) |
|  | 0.7 – 1.8 | Koper, Slovenia* | (Šmuc et al., 2018) |
|  | 0.5 – 5.3 | Naples, Italy* | (Adamo et al., 2005) |
|  | **n/d – 385** | **Fort Lauderdale, Florida, USA*** | **This Study** |
|  | **n/d – 3.61** | **West Lake, FL, USA** | **This Study** |
|  | **n/d – 0.040** | **Reef Sites Fort Lauderdale, FL** | **This Study** |
| Ni | 3 – 20 | New South Wales, Australia* | (Jahan and Strezov, 2018) |
|  | 61.3 – 109.4 | Koper, Slovenia* | (Šmuc et al., 2018) |
|  | 3.2 – 47.1 | Laizhou Bay and Zhangzi Island, China | (Zhuang and Gao, 2014) |
|  | 20-24 | Estaque, France* | (Mamindy-Pajany et al., 2013) |
|  | 15-19 | Saint Mandrier, France* | (Mamindy-Pajany et al., 2013) |
|  | 14 - 58 | 19 England estuaries sites | (Kennish, 1997) |
|  | 2-240 | 52 coastal world sites | (Qian et al., 2015) |
|  | **0.232 – 29.3** | **Fort Lauderdale, Florida, USA*** | **This Study** |
|  | **0.438 – 16.8** | **West Lake, FL, USA** | **This Study** |
|  | **0.410 – 0.860** | **Reef Sites Fort Lauderdale, FL** | **This Study** |
| Se | 0.4 – 8.8 | New South Wales, Australia | (Peters et al., 1999) |
|  | 0.2 – 1.7 | Solomon River, Kansas, USA | (May et al., 2007) |
|  | 0.2 – 0.3 | Kuskokwim River, Alaska, USA | (Belkin and Sparck, 1993) |
|  | n/d – 1.51 | 19 England estuaries sites | (Kennish, 1997) |
|  | **n/d – 4.79** | **Fort Lauderdale, Florida, USA*** | **This Study** |
|  | **0.115 – 0.885** | **West Lake, FL, USA** | **This Study** |
|  | **0.040 – 0.120** | **Reef Sites Fort Lauderdale, FL** | **This Study** |
| Sn | 3 – 37 | New South Wales, Australia* | (Jahan and Strezov, 2018) |
|  | 0.40 - 161 | 19 England estuaries sites | (Kennish, 1997) |
|  | **n/d – 140** | **Fort Lauderdale, Florida, USA*** | **This Study** |
|  | **0.064 – 7.70** | **West Lake, FL, USA** | **This Study** |
|  | **0.990 – 2.07** | **Reef Sites Fort Lauderdale, FL** | **This Study** |
| V | 2.5 – 13.5 | New South Wales, Australia* | (Jahan and Strezov, 2018) |
|  | 18 – 94 | Naples, Italy* | (Adamo, 2005) |
|  | 30.6 – 32.5 | Persian Gulf, Iran* | (Abdollahi, 2013) |
|  | **0.160 – 176** | **Fort Lauderdale, Florida, USA*** | **This Study** |
|  | **0.432 – 59.6** | **West Lake, FL, USA** | **This Study** |
|  | **3.80 – 9.12** | **Reef Sites Fort Lauderdale, FL** | **This Study** |
| Zn | 7 – 2345 | New South Wales, Australia* | (Jahan and Strezov, 2018) |
|  | 54.0 – 99.0 | Koper, Slovenia* | (Šmuc et al., 2018) |
|  | 12.8 – 88.6 | Laizhou Bay and Zhangzi Island, China | (Zhuang and Gao, 2014) |
|  | 41 – 1196 | Naples, Italy* | (Adamo, 2005) |
|  | 329 - 538 | Estaque, France* | (Mamindy-Pajany, 2013) |
|  | 209 - 221 | Saint Mandrier, France* | (Mamindy-Pajany, 2013) |
|  | 46 – 2821^#^ | 19 England estuaries sites | (Kennish, 1997) |
|  | 7 - 4430 | 52 coastal world sites | (Qian et al., 2015) |
|  | 32.00 – 433.75 | 40 U.S. estuaries sites | (Kennish, 1997) |
|  | **0.112 – 603** | **Fort Lauderdale, Florida, USA*** | **This Study** |
|  | **0.409 – 49.8** | **West Lake, FL, USA** | **This Study** |
|  | **2.76 – 91.1** | **Reef Sites Fort Lauderdale, FL** | **This Study** |

* port; ^#^ location with contaminated acid mine waste; n/d = non-detected.

**References**

Abdollahi, S., Raoufi, Z., Faghiri, I., Savari, A., Nikpour, Y., Mansouri, A. (2013). Contamination levels and spatial distributions of heavy metals and pahs in surface sediment of imam khomeini port, Persian Gulf, Iran. *Mar. Poll. Bull., 71*(1-2), 336–345. doi:<https://doi.org/10.1016/j.marpolbul.2013.01.025>

Adamo, P., Arienzo, M., Imperato, M., Naimo, D., Nardi, G., Stanzione, D. (2005). Distribution and partition of heavy metals in surface and sub-surface sediments of Naples City Port. *Chemosphere, 61*(6), 800–809. doi:<https://doi.org/10.1016/j.chemosphere.2005.04.001>

Choi, K. Y., Kim, S. H., Hong, G. H., and Chon, H. T. . (2011). Distributions of heavy metals in the sediments of South Korean Harbors. *Environ. Geochem. Health, 34*(1), 71–82. doi:<https://doi.org/10.1007/s10653-011-9413-3>

Jahan, S., Strezov, V. (2018). Comparison of pollution indices for the assessment of heavy metals in the sediments of seaports of NSW, Australia. *Mar. Pollut. Bull.*, 295–306. doi:<https://doi.org/10.1016/j.marpolbul.2018.01.036>

Kennish, M. J. (1997). *Practical Handbook of Estuarine and Marine Pollution* (1st ed.). Boca Raton: CRC Press.

Mamindy-Pajany, Y. H., C., Géret, F., Galgani, F., Battaglia-Brunet, F., Marmier, N., Roméo, M. (2013). Arsenic in marine sediments from French Mediterranean ports: Geochemical partitioning, bioavailability and ecotoxicology. *Chemosphere, 90*(11), 2730-2736. doi:<https://doi.org/10.1016/j.chemosphere.2012.11.056>

Peters, G. M., Maher, W. A., Krikowa, F., Roach, A. C., Jeswani, H. K., Barford, J. P., Gomes, V. G., Reible, D. D. (1999). Selenium in sediments, pore waters and benthic infauna of Lake Macquarie, New South Wales, Australia. *Mar. Environ. Res., 47*(5), 491–508. doi:<https://doi.org/10.1016/s0141-1136(99)00027-6>

Rogan Šmuc N, Dolenec M, Kramar S, Mladenović A. Heavy Metal Signature and Environmental Assessment of Nearshore Sediments: Port of Koper (Northern Adriatic Sea). Geosciences. 2018; 8(11):398. <https://doi.org/10.3390/geosciences8110398>

Tavakoly Sany, S. B., Salleh, A., Halim Sulaiman, A., Sasekumar, A., Tehrani, G., Rezayi, M. . (2012). Distribution characteristics and ecological risk of heavy metals in surface sediments of West Port, Malaysia. *Environ. Prot. Eng., 38*(4), 139-155. doi:<https://doi.org/10.37190/epe120412>

Qian, Y., Zhang, W., Yu, L. Feng, H. (2015). Metal Pollution in Coastal Sediments. *Curr. Poll. Rep. 1*, 203–219. doi:<https://doi.org/10.1007/s40726-015-0018-9>

Zhuang W., Gao. X. (2014). Integrated assessment of heavy metal pollution in the surface sediments of the Laizhou Bay and the coastal waters of the Zhangzi Island, China: comparison among typical marine sediment quality indices. *PLoS One, 9*(4), 1-17. doi:10.1371/journal.pone.0094145
